# Relative effectiveness of antidepressant treatments in treatment-resistant depression: a systematic review and network meta-analysis of randomized controlled trials

**DOI:** 10.1038/s41386-024-02044-5

**Published:** 2024-12-30

**Authors:** Johan Saelens, Anna Gramser, Victoria Watzal, Carlos A. Zarate, Rupert Lanzenberger, Christoph Kraus

**Affiliations:** 1 Department of Psychiatry and Psychotherapy, Medical University of Vienna, Vienna, Austria; 2 Comprehensive Center for Clinical Neurosciences and Mental Health, Medical University of Vienna, Vienna, Austria; 3 Experimental Therapeutics and Pathophysiology Branch, National Institute of Mental Health, National Institutes of Health, Bethesda, MD USA

**Keywords:** Depression, Outcomes research

## Abstract

This systematic review and network meta-analysis (NMA) sought to compare different antidepressant treatments for treatment-resistant depression (TRD) in order to facilitate evidence-based choices. A literature search of PubMed, Cochrane Library, and Embase from inception until April 13th, 2023 identified randomized, controlled trials (RCTs) of adults with depression who had not responded to at least two antidepressant trials; all RCTs had ≥10 participants per study arm, and participants with bipolar or psychotic depression were excluded. The Cochrane Risk of Bias Tool-2 was used to assess study quality. Response rate was the primary outcome measure. Odds ratios (ORs) using a random effects NMA are reported. From 8234 records, 69 RCTs were included in this analysis, encompassing 10,285 participants (5662 F/4623 M) and 25 separate treatments. Six of the 25 treatments demonstrated a higher response rate versus placebo or sham treatment: electroconvulsive therapy (ECT), minocycline, theta-burst stimulation (TBS), repetitive transcranial magnetic stimulation (rTMS), ketamine, and aripiprazole. ORs ranged from 1.9 (95%CI = [1.25; 2.91]) for aripiprazole to 12.86 (95%CI = [4.07; 40.63]) for ECT. Moderate heterogeneity of the model was observed (I^2^ = 47.3% (95%CI [26.8–62%]). Of the included studies, 12.5% were rated as having high risk of bias, 28.13% as having low risk, and 59.38% as showing some concerns. Several effective treatments for TRD showed robust treatment effects across outcomes (ECT, TBS, rTMS, and ketamine), and others showed promising results for some, but not all, outcomes (minocycline, aripiprazole). These findings may help guide evidence-based treatment choices for TRD. Study Registration: PROSPERO (#CRD42023420584).

## Introduction

Despite the availability of numerous treatments for major depressive disorder (MDD), nearly half of individuals do not respond to at least two antidepressants, thereby meeting criteria for treatment resistance [1, 2]. Strategies to address treatment-resistant depression (TRD) include optimizing dosages, switching antidepressants, or augmenting treatment with a nonstandard agent. Another treatment approach involves augmenting monoaminergic drugs like selective serotonin reuptake inhibitors (SSRIs) with mood stabilizers or second-generation antipsychotics. In addition, administration of subanesthetic-dose ketamine, which has been shown to alleviate depressive symptoms within hours, led to the approval of its enantiomer, (*S*)-ketamine, for the treatment of TRD in 2019 [3]. There has also been a resurgence of clinical trials investigating serotonergic psychedelics (SPs) such as psilocybin and N,N-dimethyltryptamine (DMT) for various psychiatric conditions, including depression. Another potential treatment for TRD is neuromodulatory procedures, which can be either invasive (e.g., deep brain stimulation (DBS)) or non-invasive (e.g., repetitive transcranial magnetic stimulation (rTMS) or electroconvulsive therapy (ECT)). Despite considerable research evidence surrounding these treatment modalities—some novel, some in use for decades— it is unclear how their efficacies compare.

While previous NMAs have investigated treatment strategies for TRD [4–6], to date none have also compared recent evidence drawn from ketamine or SP studies or compared pharmacological augmentation to neuromodulatory procedures such as ECT, DBS, rTMS, or transcranial direct current stimulation (tDCS). This NMA compared the effectiveness of a variety of available antidepressant treatments for TRD, examining both individual antidepressants and classes of antidepressants as well as other medicines with antidepressant effects. In addition, while previous NMAs used a more lenient definition of TRD (lack of response to at least one treatment trial [7, 8]), this study used the definition endorsed by regulatory authorities such as the US Food and Drug Administration (FDA) and the European Medicines Agency (EMA), which requires lack of response to two or more treatment trials [1, 2].

## Materials and methods

### Search strategy and selection criteria

The study adhered to the Preferred Reporting Items for Systematic Reviews and Meta-Analyses (PRISMA) and its extension statement for NMAs [9]. The study protocol was registered with the international prospective register of systematic reviews (PROSPERO) (ID#: CRD42022324095).

A literature search of PubMed, Embase, and the Cochrane Central Register of Controlled Trials was conducted with English language restrictions from inception until April 13th, 2023 (Supplementary Table S1.1–S1.3). Inclusion of all relevant clinical trials was confirmed by consulting clinicaltrials.gov, the International Clinical Trials Registry Platform, and existing relevant meta-analyses. In cases of missing, unpublished, or conflicting data, the original study authors were contacted for clarification.

Inclusion criteria included randomized, controlled trials comparing medicines with antidepressant effects in adults ( ≥ 18 years old) with MDD who did not respond to ≥2 antidepressant treatments. Included studies had to have a minimum of 10 participants per study arm and not be limited to a particular patient population (e.g., only patients with a particular comorbid disorder). Studies that included participants with bipolar depression or depression with psychotic features were excluded. Because it was expected that most treatments would be used adjunctively, additional antidepressant treatments were permitted as long as they remained constant during the trial. Trials where treatment resistance was measured prospectively before initiating further antidepressant treatment were also included. In cases where patients could choose between different broad treatment options and were then randomized to a specific treatment, the treatment arms were treated as separate comparisons within our model.

Twenty-five identified treatments were allocated into six groups according to their primary clinical use or mechanism of action. These included: mood stabilizers (lithium, sodium valproate, and lamotrigine); antipsychotics (aripiprazole, brexpiprazole, olanzapine, quetiapine, quetiapine monotherapy, risperidone); N-methyl-D-aspartate receptor (NMDAR)-targeting agents (ketamine, D-cyloserine, lanicemine, minocycline, and nitrous oxide); SPs (ayahuasca and psilocybin); neuromodulatory treatments (DBS, ECT, rTMS, theta-burst stimulation (TBS), and tDCS); and other pharmacological treatments (buspirone, fluoxetine, olanzapine/fluoxetine combination, and thyroid hormone).

Two investigators (JS and CK) independently screened abstracts for relevant studies and resolved discrepancies via discussion. The data were then collected by one researcher (JS) and checked by another (AG). One researcher (JS) assessed study risk of bias using the Cochrane Collaboration’s Tool 2 (see below); this rating was then compared to the ratings of two other researchers (AG and VW).

### Data analysis

The primary efficacy outcome was response rate (defined as ≥50% reduction in depressive symptoms measured by standardized depression scales that varied across studies). A validated imputation method was used to calculate response rate when it was not provided (number of participants at endpoint*normal standard distribution corresponding with (50% of the baseline score minus endpoint score)/standard deviations (SD)). If more than one time point was measured, the nearest time point after the last treatment dose given to participants was used. When possible, intention-to-treat data were used. In a conservative approach, participants excluded from their respective analyses without providing data were treated as having a negative outcome in our analysis. To compare the severity of depressive symptoms, a mapping formula was used to convert Hamilton Depression Rating Scale-17 (HAM-D) scores and respective SDs to Montgomery-Asberg Depression Rating Scale (MADRS) scores (MADRS = 1.04×HAM-D17 + 10.13) [10]. Secondary outcomes included remission (MADRS score of ≤10 or a HAM-D score of ≤7), endpoint depression scores, and tolerability (defined as the proportion of participants who withdrew from a study due to adverse events). If SDs were not provided, the RevMan Calculator was used to impute them based on the provided data [11]. Log odds ratios (ORs) were used for all dichotomous outcomes, and Hedge’s g was used for interval-scaled outcomes with a 95% confidence interval.

Statistical analyses were conducted using R (version 4.0.5) and the Netmeta package (version 2.0-1). The NMA was done within a frequentist framework. The heterogeneity of treatment effects was assessed using I^2^ statistics and computed total inconsistency based on a full design-by-treatment interaction random-effects model.

The NMA of the primary outcome was duplicated within a Bayesian framework using the gemtc package, using a noninformative prior. Posterior distributions of parameters were estimated using Markov Chain Monte Carlo sampling, using 500,000 iterations and 5000 burn-in iterations (Supplementary Figs. S9.1–S9.3).

The group analysis examined six different therapeutic categories: antipsychotics, mood stabilizers, NMDAR-targeting agents, neuromodulatory treatments, SPs, and a group of other pharmacological treatments. The robustness of the analyses was examined via several sensitivity analyses that included the following data subsets: non-sponsored trials, studies with low and moderate risk of bias, placebo-controlled trials, trials where participants were blinded to treatment, and trials where response rate was provided. Meta-regression was used to explore a possible effect of baseline severity of depression, age, sex, and year of publication on the primary outcome.

With regard to ketamine in particular, different routes of administration as well as racemic ketamine versus the intranasally-administered enantiomer (*S*)-ketamine were investigated via subgroup analyses that combined intravenous (IV) and intranasal ketamine. A meta-regression was conducted to see if the use of an active placebo reduced the effect of ketamine. Response rates were compared first, and a separate analysis was then conducted to assess the comparability of drug placebo and sham conditions. Other forms of placebo were also grouped together.

The distribution of several variables was examined to check for potential violations of the transitivity assumption; these included severity of baseline symptoms, duration of the current depressive episode, comorbidities, concurrent medications, age, and sex. Publication bias was checked using comparison-adjusted funnel plots to visually evaluate asymmetry and Egger’s test to statistically evaluate asymmetry.

## Results

### Study characteristics

**Fig. 1:**
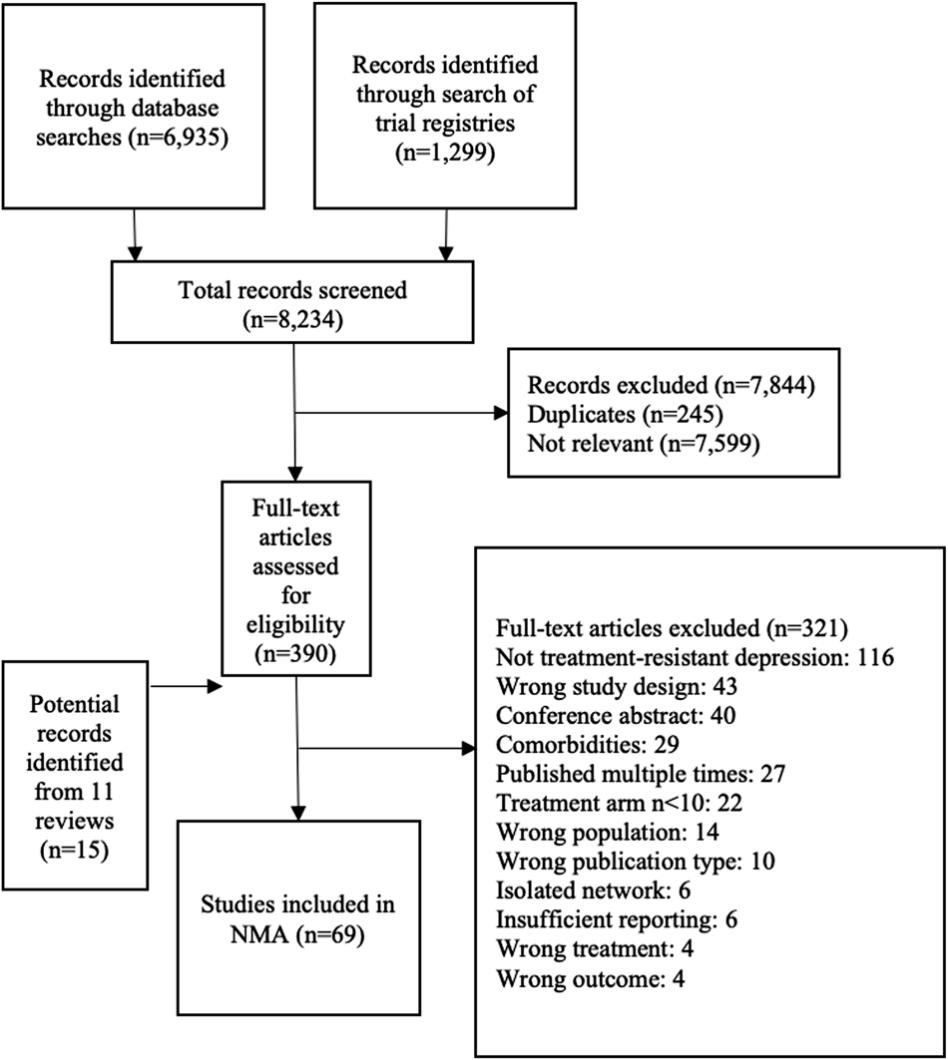
Study selection process. NMA=network meta-analysis.

A total of 8234 abstracts were screened, and 390 full-texts were retrieved for further inspection (Fig. 1). The final analysis included 69 studies with a total of 10,285 participants and 25 different treatments grouped into six categories based on mechanism of action (Supplementary Table S2, Figs. S2.1–2.2). The mean age was 43.73 years (SD = 11.29), and 5662 (55.05%) participants were female. The mean duration of the included studies was 5.07 weeks. Most trials were placebo-controlled (*n* = 59, 85.51%) and blinded (*n* = 62, 89.86%). Twenty-seven (39.13%) studies were funded by private companies. Response rates were imputed for 105 participants (1.02%).

Regarding clinical characteristics, participants had, on average, not responded to 4.33 antidepressant trials (SD 1.96), although only 17 studies (24.64%) reported the exact number of trials. The mean baseline MADRS score of participants was 33 (SD = 6), corresponding to a moderate level of depression. The mean duration of the current depressive episode was 33 months (SD = 38.64). Eighteen studies (26.09%) included patients with a comorbid Axis I disorder. Most studies (*n* = 62, 89.86%) included participants taking additional antidepressants.

### Network meta-analysis

**Fig. 2:**
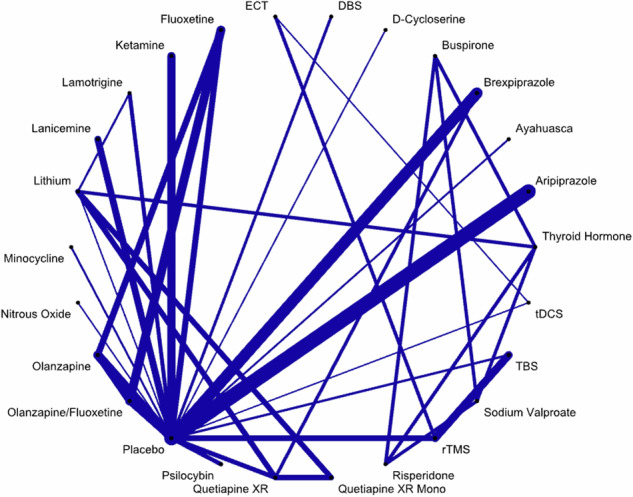
Network graph for response rate. The width of the lines is proportional to the number of participants compared between two treatments. Lines indicate trials with direct comparisons. DBS=deep brain stimulation; ECT=electroconvulsive therapy; tDCS=transcranial direct current stimulation; TBS=theta burst stimulation; rTMS=repetitive transcranial magnetic therapy; XR=extended release.

The network for the outcome response rate consisted of 67 randomized, controlled trials with 9354 participants (two studies did not provide enough information to calculate response rates and where thus excluded from the primary analysis). Figure 2 shows the network for all compared treatments. There was at least one placebo-controlled trial for each treatment except for ECT, thyroid hormone, quetiapine XR monotherapy, risperidone, sodium valproate, and buspirone.

**Fig. 3:**
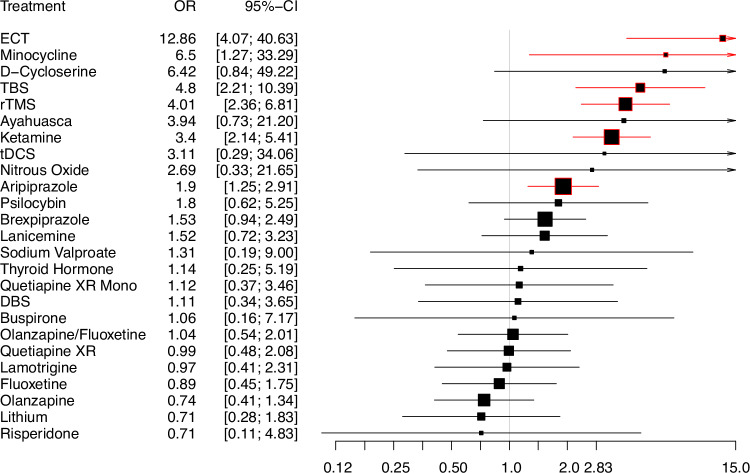
Forest plot for response rate to antidepressant treatments for treatment-resistant depression compared to placebo or sham. Significant results are displayed in red. Size of squares is proportional to number of participants included for each treatment. DBS=deep brain stimulation; tDCS=transcranial direct current stimulation; rTMS=repetitive transcranial magnetic stimulation; TBS=theta-burst stimulation; ECT=electroconvulsive therapy; OR=odds ratio; CI=confidence interval.

Figure 3 shows the efficacy results for the primary outcome. Six of the 25 included treatments were effective compared to placebo: ECT, minocycline, TBS, rTMS, ketamine, and aripiprazole. All had ORs ranging from 1.9 (95%CI = [1.25; 2.91]) for aripiprazole to 12.86 (95%CI = [4.07; 40.63]) for ECT.

**Fig. 4:**
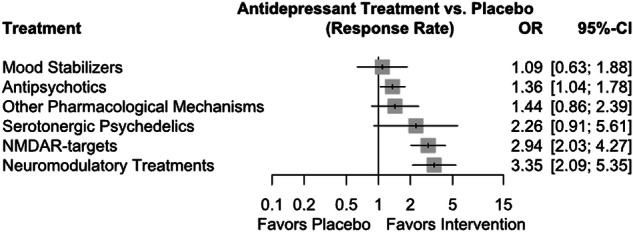
Forest plot for response rate of antidepressant treatment groups compared to placebo. NMDAR = N-methyl-D-aspartate receptor.

Figure 4 displays the results of the analysis of different groups of antidepressants (Supplementary Figs. S6.1-6.2, Table S6). Three of the six groups of antidepressant treatments demonstrated a higher response rate than placebo: neuromodulatory treatments (OR = 3.35 (95%CI = [2.09; 5.35])), NMDAR targets (OR = 2.94 (95%CI = [2.03; 4.27])), and antipsychotics (OR = 1.36 (95%CI = [1.04; 1.78])).

Regarding secondary outcomes, only ECT, TBS, rTMS, and ketamine had significantly lower scores at endpoint than placebo, with ORs ranging from −1.43 to −0.73 (Supplementary Figs. S5.2–5.3). Remission was significantly different from placebo for ECT, TBS, rTMS, ketamine, aripiprazole, and the olanzapine/fluoxetine combination (Supplementary Figs. S5.5–5.6). Finally, regarding tolerance, the use of quetiapine, olanzapine, fluoxetine, olanzapine/fluoxetine combination, aripiprazole, and brexpiprazole significantly increased drop-out rate due to adverse effects, with ORs ranging from 2.23 to 5.63 (Supplementary Figs. S5.8–5.9). Results of the pairwise meta-analysis for all outcomes are shown in Supplementary Figs. S8.1–S8.4. Results of direct comparisons between treatments are shown in Supplementary Tables S5.1*–*S5.4.

### Ranking probabilities

P-Score was used to rank the probability that an included treatment would be the most effective (Supplementary Figs. S5.1, S5.4, S5.7, S5.10). ECT ranked first for response rate (P-Score = .96) and endpoint depression score (P-Score = 0.82) and second for remission (P-Score = 0.9). TBS ranked first for remission (P-Score = 0.9). Lamotrigine was the best tolerated treatment (P-Score = 0.16), and olanzapine the least tolerated (P-Score = 0.75).

### Analysis of heterogeneity

The response rate heterogeneity of the model was moderate, with an I^2^ of 47.3% (95%CI = [26.8%-62%]). Both significant between-design (Q = 18.53, *p* = 0.018) and within-design heterogeneity (Q = 76.27, *p* = 0.001) were observed. When assuming a full design-by-treatment random-effects model, between-design inconsistency was no longer significant (Q = 6.79, *p* = 0.56). Between-design inconsistency was further examined using the netheat function (Supplementary Figs. S7.1–S7.4).

### Sensitivity and subgroup analyses

The results of scenario analyses demonstrated the robustness of most of the main findings (Supplementary Figs. S10.1–10.7). However, ECT was no longer significant compared to sham when imputed studies, those with a high risk of bias, unblinded studies, or studies without a placebo condition were excluded. Minocycline also demonstrated no significant effect when only non-commercially sponsored studies were included; this resulted in removing about half of the total studies, which increased general uncertainty in this network. It should also be noted that there was no significant effect for aripiprazole in this analysis because no non-commercially funded studies exist for this treatment. Meta-regression analysis, which evaluated the effects of baseline depression score, year of publication, age, and sex on the primary outcome found that none of these factors had a significant impact (Supplementary Table S12).

Furthermore, subgroup analyses found no significant differences between response rate to racemic ketamine and (*S*)-ketamine when pooling routes of administration (Supplementary Table S11.1, Fig. S11.1). However, only racemic ketamine had a significantly higher response rate compared to placebo. A more detailed subgroup analysis, separated further by both form (esketamine versus racemic ketamine) and route of administration (IV, intranasal, and oral), similarly demonstrated a significant effect for IV-administered racemic ketamine versus placebo (Supplementary Table S11.2, Fig. S11.2). In direct comparisons, no significant differences were observed between different forms of ketamine delivered via various routes of administration. A subgroup analysis including the TRANSFORM 1-3 studies, which were pivotal for the approval of (*S*)-ketamine for TRD, tested (*S*)-ketamine in combination with a newly initiated oral antidepressant and found that this treatment combination had a significant effect (Supplementary Figs. S11.3–S11-5). However, both racemic ketamine overall and IV-administered racemic ketamine specifically were more effective than (*S*)-ketamine, with or without an oral antidepressant (Supplementary Tables S11.3–S11.4). These studies were excluded from the main analysis due to transitivity concerns arising from their design, which added a newly initiated oral antidepressant to both the treatment and placebo conditions.

Finally, to evaluate whether pooling sham and placebo arms was appropriate, response rates for both conditions were compared. Placebo arms had an average response rate of 22.48% (range = 0–49.15%) compared to sham conditions, demonstrating a response rate of 14.07% (range = 0–72.72%). In this context, an additional analysis was conducted that separated sham versus placebo conditions (Supplementary Figs. S10.1–S10.2); similar results to the main analysis were observed with the exception that brexpiprazole reached significance (OR = 1.53 (95%CI = [1.03; 2.27])).

### Quality assessment and publication bias

Most studies were associated with moderate risk of bias (*n* = 42; 60.9%). Nine studies (13%) were rated as having high risk of bias, and 18 studies (26.1%) were rated as having low risk (Supplementary Table S3). No indication of publication bias was noted (Supplementary Figs. S4.1–S4.4).

## Discussion

This NMA of 69 randomized, controlled trials comprising 10,285 participants and 25 different therapies for TRD found that six of the 25 included treatments were associated with significantly higher response rates than placebo: ECT, minocycline, TBS, rTMS, ketamine, and aripiprazole. The strongest effects were found for neuromodulatory treatments followed by antipsychotics and agents that target the NMDAR. Notably, this study is the first to incorporate neuromodulatory treatments alongside both established antidepressants and novel, rapid-acting antidepressants [6]. Previous analyses either did not incorporate recent evidence from ketamine and SPs [4] or excluded neuromodulatory treatments [5, 7, 8] and other pharmacological agents [6, 12]. Previous studies also used a broader definition of TRD that allowed for lack of response to just one rather than two antidepressant trials; the latter is the accepted definition of treatment-resistance adopted by the EMA and FDA [1, 2, 7, 8, 12].

In addition to the six treatments associated with significantly higher response rates than placebo, ECT, TBS, rTMS, and ketamine were also effective when considering both total endpoint depression score and remission. Aripiprazole and the olanzapine/fluoxetine combination treatment were effective when considering remission only. The study also found that antipsychotics (quetiapine, olanzapine, aripiprazole, and brexpiprazole) as well as fluoxetine and olanzapine/fluoxetine combination treatment were significantly less well tolerated than placebo. While these results are based on a substantial amount of evidence for most treatments (e.g., aripiprazole, *n* = 1933; rTMS, *n* = 1810; ketamine, *n* = 1109; TBS, *n* = 683; olanzapine/fluoxetine combination, *n* = 477), for other treatments fewer studies meeting relevant inclusion criteria were available (e.g., minocycline, *n* = 41; ECT, *n* = 208).

Interestingly, this analysis found that ECT was most likely to be the most efficacious treatment, though it should be noted that few randomized, controlled trials exist for ECT. Previous NMAs drawing from head-to-head studies found mixed results regarding the efficacy of ECT for TRD [6, 8, 12]. Our analysis relied on three head-to-head studies that compared ECT to rTMS or tDCS. Consistent with our findings, a recent head-to-head study suggested that ECT may be more effective than ketamine in participants with MDD, though treatment resistance was not explicitly defined [13]. However, the largest comparative effectiveness trial to date, which included mostly outpatients with TRD, found that ketamine was noninferior to ECT [14]. Nevertheless, that study also found a considerable preference for ketamine (31 participants receiving ECT dropped out of the study versus four receiving ketamine), and typical responders to ECT may have been underrepresented (eg, inpatients or those with severe or catatonic depression). Broadly, however, the evidence is limited; there has been no sham-controlled study of ECT for either TRD or non-TRD since 1985.

Crucially, convincing antidepressant effects were also observed for other neuromodulatory treatments such as rTMS and TBS, consistent with previous evidence [4, 6, 12]. Robust effects were noted across all efficacy outcomes, and these were sustained in sensitivity analyses; good tolerability was also observed. Both rTMS and TBS were significantly more effective than aripiprazole but showed only a numerical advantage compared to ketamine. However, similar to previous studies, ECT was found to be superior to rTMS [15]. It should be noted that TBS was noninferior to rTMS in the present analysis, which may justify use of this more convenient modality.

Echoing previous analyses, this study found promising outcomes for ketamine and, to a lesser degree, other NMDAR-targeting medications like minocycline [5, 6, 16]. This finding is corroborated by recent evidence demonstrating the superior efficacy of ketamine compared to the traditional augmentative agent quetiapine [17]. Interestingly, the only differences observed between different forms of ketamine were found when including the TRANSFORM studies, specifically between intranasal (*S*)-ketamine, with or without an additional oral antidepressant, and IV-administered ketamine. This finding aligns with the results of Bahji and colleagues, who reported a tendency for IV racemic ketamine to be more efficacious than intranasal (*S*)-ketamine [18]. However, racemic IV ketamine studies were generally of shorter duration, highlighting the need for research on the long-term effects of this treatment.

Interestingly, other NMDAR antagonists such as nitrous oxide and lanicemine exhibited no significant antidepressant effects in our analysis. In this context, it should be noted that ketamine may exert its antidepressant effects via several different mechanisms of action [19]. For example, anti-inflammatory processes appear to play a role in the antidepressant effects of both ketamine and minocycline [19, 20]. Nevertheless, a recent systematic review [21] that also included a large randomized controlled trial with 173 patients who had not responded to only one previous antidepressant [22] found inconclusive results. It is also important to note that this analysis included only a single study on minocycline, which also stood out as an outlier in terms of severity of depressive symptoms (mean HAM-D score = 34.5). Broadly, however, the existing evidence suggests that individuals with more severe depression tend to exhibit a better response to antidepressants but a poorer response to placebo [23], which may have inflated the effect of minocycline.

Antipsychotic augmentation with quetiapine or brexpiprazole, despite being FDA-approved, was generally not effective for treating TRD in our analysis. Similar to a prior analysis [16], aripiprazole was an exception and displayed modest antidepressant effects. Consequently, the significant group-level effect of antipsychotics was largely attributable to the evidence supporting the efficacy of aripiprazole. This particularity might be attributable to aripiprazole’s distinct receptor profile compared to other atypical antipsychotics; specifically, it acts as a partial agonist at the 5-HT_1A, D2_, and _D3_ receptors and as a 5-HT_2A_ receptor antagonist. In support of this hypothesis, brexpiprazole—considered the successor to aripiprazole and also displaying activation at the 5-HT_1A, D2, D3_, and 5-HT_2A_ receptors—had the second largest effect size among antipsychotics after aripiprazole. Brexpiprazole’s lack of efficacy in the present analysis may be explained by our use of response rate—a more conservative measure—whereas the original studies used change in depressive symptom score as their primary outcome. Notably, all six aripiprazole and all five brexpiprazole trials included in our analysis were sponsored by pharmaceutical companies, whereas the evidence for all other effective treatments was at least partially drawn from trials not sponsored by industry. Regarding quetiapine, it should be noted that FDA approval was based on studies that adopted the less stringent definition of treatment resistance as lack of response to a single antidepressant and which were thus not included here [24]. Moreover, when analyzing antidepressant classes, both neuromodulatory treatments and NMDAR-targeting agents were more efficacious than antipsychotic augmentation. Antipsychotics were also the least tolerated drug class in our analysis. Specifically, antipsychotic treatments have been associated with a range of side effects, including metabolic dysfunction, weight gain, tardive dyskinesia, and possibly increased mortality compared to other antidepressants [25, 26]. These results require the cautious use of these compounds as augmentative strategies for TRD.

Interestingly, no significant effect was seen for lithium in treating TRD, in contrast to previous analyses [27, 28]. However, this discrepancy may have been due to the fact that prior analyses defined TRD as lack of response to one antidepressant rather than two as well as the inclusion of individuals with bipolar depression. Another point of interest is that this NMA is the first to also include SPs, specifically psilocybin and ayahuasca. Despite moderate ORs, these agents displayed no significant antidepressant effects. Subsequent studies are needed to ascertain whether these observed trends can be substantiated.

Despite these valuable findings, the analysis is also associated with several limitations. First, most of the included studies were conducted over a relatively brief period that rarely exceeded 6-8 weeks. As a salient example of the implications of this limitation, fluoxetine studies found that 31-41% of patients who showed no improvement after 6 weeks nevertheless achieved remission by week 12 [29]. Second, time points at which outcomes were assessed varied across studies, with some outcomes measured within days (e.g., ketamine) and others over months (e.g., DBS). While most studies had a duration of several weeks, this discrepancy still limits the comparability of treatments based on a uniform timeline. Third, our analysis could not determine the longevity of the observed antidepressant effects. Addressing these questions requires more time-consuming and ethically complex research comparing treatments over several months to years. Fourth, our study provides no evidence regarding individual or group-level factors that might influence the effect of a given treatment, such as comorbidities or combination with another antidepressant treatment. Relatedly, the included trials were conducted over a timespan of several decades and, thus, methodological quality and the characteristics of included patients might vary. However, the sensitivity analyses largely demonstrated the robustness of our results across various factors such as severity of depressive symptoms, sex, and age. The lack of significant effects for some treatments (e.g., ECT, minocycline) but not others (e.g., rTMS, ketamine) in specific sensitivity analyses underscores the scarcity of the evidence for these treatments. Finally, alternative treatment approaches (e.g., psychotherapy, light therapy, sleep deprivation, aerobic exercise) were excluded from our analysis. The challenge of employing a placebo for these treatments complicates their comparison with the included therapies. Nevertheless, there is evidence for the efficacy of psychotherapy plus antidepressant treatment compared to antidepressant treatment alone for TRD [30].

In conclusion, the findings of this NMA underscore the efficacy of several available treatments for TRD, including ECT, rTMS, TBS, ketamine, aripiprazole, minocycline, and the combination of olanzapine/fluoxetine; the first four treatments demonstrated efficacy across all outcomes. The analysis also found no effects for mood stabilizers and antipsychotics other than aripiprazole and a low tolerance for all included antipsychotics. These results provide valuable information for researchers and clinicians, and the novel evidence presented herein may help guide the selection of antidepressant treatments for individuals with TRD.

## Supplementary information

Supplemental Material

PRISMA Checklist für NMA

## Data Availability

Data not included in the article or supplementary materials can be obtained from the corresponding author upon request.
