# Supplementary material for: Relative effectiveness of antidepressant treatments in treatment-resistant depression: a systematic review and network meta-analysis of randomized controlled trials
Source: Neuropsychopharmacology. 2024 Dec 30;50(6):913–9. doi: 10.1038/s41386-024-02044-5 (PMC12032262; doi:10.1038/s41386-024-02044-5)
Supplement: Supplementary file 1 — Supplemental Material [file 41386_2024_2044_MOESM1_ESM.docx]

*Saelens et al — Relative Effectiveness of Antidepressant Treatments in Treatment-Resistant Depression: A Systematic Review and Network Meta-Analysis of*

*Randomized Controlled Trials*

**Supplementary Material**

Table of Contents

[1. Search strategy 2](#_Toc167794403)

[2. Demographics 4](#_Toc167794404)

[3. Risk of bias assessment 14](#_Toc167794405)

[4. Assessment of publication bias 17](#_Toc167794406)

[5. Analysis of efficacy 20](#_Toc167794407)

[6. Group-level analysis 40](#_Toc167794408)

[7. Analysis of inconsistency 43](#_Toc167794409)

[8. Pairwise Meta-Analysis 47](#_Toc167794410)

[9. Bayesian Network Meta-Analysis 56](#_Toc167794411)

[10. Scenario Analyses 59](#_Toc167794412)

[11. Subanalyses 62](#_Toc167794413)

[12. Meta-regression 65](#_Toc167794414)

[13. Supplementary references 66](#_Toc167794415)

# 1. Search strategy

A literature search was conducted from inception until January 30^th^, 2021 and subsequently updated to include literature from inception until April 13^th^, 2023.

## **Supplementary Table S1.1. PubMed**

| **SEARCH** | **Number** |
| --- | --- |
| **Disease terms** |  |
| "Depressive Disorder, Treatment-Resistant"[Mh] | #1 |
| (treatment-resistan* [tiab] OR therapy-resistan* [tiab] OR refract* [tiab] OR resistan* [tiab] OR nonrespon* [tiab] OR unrespon* [tiab] OR fail* [tiab] OR “incomplete response” [tiab] OR “no respon*” [tiab] OR “inadequate response” [tiab] OR “sub-optimal respon*” [tiab] OR “poor response” [tiab] OR “residual symptoms” [tiab] OR “drug resistan*” [tiab] OR “treatment failure” [tiab]) AND depress* [tiab]) | #2 |
| #1 OR #2 | #3 |
| **Randomized controlled trial filter** |  |
| randomized controlled trial [pt] | #4 |
| controlled clinical trial [pt] | #5 |
| randomized [tiab] | #6 |
| placebo [tiab] | #7 |
| drug therapy [sh] | #8 |
| randomly [tiab] | #9 |
| trial [tiab] | #10 |
| groups [tiab] | #11 |
| #4 OR #5 OR #6 OR #7 OR #8 OR #9 OR #10 OR #11 | #12 |
| animals [mh] not (humans [mh] and animals [mh] and animals [mh]) | #13 |
| #12 NOT #13 | #14 |
| **Interventions** |  |
| 1. **As active component** |  |
| Ketamine OR (S)-ketamine OR Citalopram OR Escitalopram OR Fluoxetine OR Fluvoxamine OR Paroxetine OR Sertraline OR Venlafaxine OR Desvenlafaxine OR Duloxetine OR Milnacipran OR Levomilnacipran OR Sibutramine OR Bicifadine OR Amitriptyline OR Amitriptylinoxide OR Butriptyline OR Clomipramine OR Desipramine OR Dibenzepin OR Dimetacrine OR Dothiepin OR Imipramine OR Lofepramine OR Melitracen OR Metapramine OR Nortriptyline OR Noxiptiline OR Pipofezine OR Propizepine OR Protriptyline OR Quinupramine OR Amineptine OR Iprindole OR Opipramol OR Tianeptine OR Trimipramine OR Bupropion OR Trazodone OR Amoxapine OR Maprotiline OR Mazindol OR Mianserin OR Setiptiline OR Isocarboxazid OR Moclobemide OR Phenelzine OR Pirlindole OR Selegiline OR Tranylcypromine OR Risperidone OR Aripiprazole OR Asenapine OR Clozapine OR Iloperidone OR Lurasidone OR Olanzapine OR Paliperidone OR Quetiapine OR Ziprasidone OR Zotepine OR Symbyax OR Mirtazapin OR Vortioxetine OR Agomelatine OR Doxepin OR Reboxetine OR Brexpiprazole OR lithium OR lamotrigine OR T3 OR triiodothyronine OR “electroconvulsive therapy” OR ECT OR tms OR transcranial magnetic stimulation OR “theta burst” OR theta-burst OR “vagus nerve stimulation” OR VNS OR “deep brain stimulation” OR DBS OR Ayahuasca OR DMT OR N,N-Dimethyltryptamine OR d-cycloserine OR Minocycline OR psilocybin OR tDCS OR “Transcranial direct current stimulation” OR “Nitrous Oxide” | #15 |
| 1. **As brand name** |  |
| Ketanest OR Ketaset OR Ketalar OR Celexa OR Cipramil OR Cipram OR Dalsan OR Recital Seropram OR Citox OR Cital OR Lexapro OR Cipralex OR Seroplex OR Depex OR Prozac OR Fontex OR Sarafem OR Ladose OR Fluctin OR Fluox OR Lovan OR Prodep OR Luvox OR Fevarin OR Faverin OR Floxyfral OR Paxil OR Seroxat OR Sereupin OR Aropax OR Deroxat OR Rexetin OR Xetanor OR Zoloft OR Lustral OR Serlain OR Tresleen OR Effexor OR Efexor OR Cymbalta OR Xeristar OR Yentreve OR Dulane OR Wellbutrin OR Budeprion OR Elontril OR Aplenzin OR Risperdal OR Parnate OR Jatrosom OR Tofranil OR Tofranil-PM OR Elavil OR Endep OR Anafranil OR Pamelor OR Aventyl Hydrochloride OR Desyrel OR Oleptro OR Beneficat OR Deprax OR Desirel OR Molipaxin OR Thombran OR Trittico OR Meresa OR Dogmatil OR Dolmatil OR Eglonyl OR Modal OR Abilify OR Saphris OR Sycrest OR Leponex OR Fanapt OR Zomaril OR Latuda OR Zyprexa OR Zalasta OR Invega OR Seroquel OR Geodon OR Zeldox OR Pristiq OR Dalcipran OR Ixel OR Savella OR Fetzima OR Meridia OR Reductil OR Elavil OR Endep OR Equilibrin OR Evadyne OR Norpramin OR Noveril OR Istonil OR Miroistonil OR Prothiaden OR Adapin OR Sinequan OR Tofranil OR Janimine OR Imiprex OR Lomont OR Gamanil OR Deanxit OR Dixeran OR Trausabun OR Timaxel OR Pamelor OR Aventyl OR Agedal OR Elronon OR Azafen OR Azaphen OR Vagran OR Vivactil OR Survector OR Prondol OR Tetran OR Insidon OR Pramolan OR Ensidon OR Stablon OR Coaxil OR Surmontil OR Asendin OR Deprilept OR Ludiomil OR Psymion OR Mazanor OR Sanorex OR Tecipul OR Marplan OR Aurorix OR Manerix OR Nardil OR Eldepryl OR Zelapar OR Emsam OR Solian OR Clozaril OR Nipolept OR Remergil OR Remeron OR Zispin OR Rexer OR Norset OR Remeron SolTab OR 6-Azamianserin OR Mepirzepine OR ORG-3770 OR Brintellix OR Valdoxan OR Melitor OR Thymanax OR Deptran OR Sinequan OR Edronax OR Prolift OR OPC-34712 OR Spravato OR Seromycin OR Minocin | #16 |
| #15 OR #16 | #17 |
| (child [mh] OR infant [mh] OR adolescent [mh] OR Review [pt] OR Systematic Review [pt] OR Meta-analysis [pt]) | #18 |
| #3 AND #17 AND #14 NOT #18 | #19 |
| Limit #19 to english language | #20 |

## **Supplementary Table S1.2. EMBASE**

| **SEARCH** | **Number** |
| --- | --- |
| **Disease terms** |  |
| 'treatment resistant depression'/exp | #1 |
| (('treatment resistan*':ab,ti OR 'therapy resistan*':ab,ti OR refract*:ab,ti OR resistan*:ab,ti OR nonrespon*:ab,ti OR unrespon*:ab,ti OR fail*:ab,ti OR 'incomplete response':ab,ti OR 'no respon*':ab,ti OR 'inadequate response':ab,ti OR 'sub-optimal respon*':ab,ti OR 'poor response':ab,ti OR 'residual symptoms':ab,ti OR 'drug resistan*':ab,ti OR 'treatment failure':ab,ti OR 'medication resistan*':ab,ti) AND depress*:ab,ti)) | #2 |
| #1 OR #2 | #3 |
| **RCT filter** |  |
| ('randomized controlled trial'/de OR ‘controlled clinical trial’/de OR random*:ti,ab,tt OR ‘randomization’/de OR ‘intermethod comparison’/de OR placebo:ti,ab,tt OR (compare:ti,tt OR compared:ti,tt OR comparison:ti,tt) OR ((evaluated:ab OR evaluate:ab OR evaluating:ab OR assessed:ab OR assess:ab) AND (compare:ab OR compared:ab OR comparing:ab OR comparison:ab)) OR (open NEXT/1 label):ti,ab,tt OR ((double OR single OR doubly OR singly) NEXT/1 (blind OR blinded OR blindly)):ti,ab,tt OR ‘double blind procedure’/de OR (parallel NEXT/1 group*):ti,ab,tt OR (parallel NEXT/1 group*):ti,ab,tt OR ((assign* OR match OR matched OR allocation) NEAR/6 (alternate OR group OR groups OR intervention OR interventions OR patient OR patients OR subject OR subjects OR participant OR participants)):ti,ab,tt OR (assigned:ti,ab,tt OR allocated:ti,ab,tt) OR (controlled NEAR/8 (study OR design OR trial)):ti,ab,tt OR (volunteer:ti,ab,tt OR volunteers:ti,ab,tt) OR ‘human experiment’/de OR trial:ti,tt) NOT ((((random* NEXT/1 sampl* NEAR/8 (‘cross section*’ OR questionnaire* OR survey OR surveys OR database or databases)):ti,ab,tt) NOT (‘comparative study’/de OR ‘controlled study’/de OR ‘randomised controlled’:ti,ab,tt OR ‘randomized controlled’:ti,ab,tt OR ‘randomly assigned’:ti,ab,tt)) OR (‘cross‐sectional study’/de NOT (‘randomized controlled trial’/de OR ‘controlled clinical study’/de OR ‘controlled study’/de OR ‘randomised controlled’:ti,ab,tt OR ‘randomized controlled’:ti,ab,tt OR ‘control group’:ti,ab,tt OR ‘control groups’:ti,ab,tt)) OR (‘case control*’:ti,ab,tt AND random*:ti,ab,tt NOT (‘randomised controlled’:ti,ab,tt OR ‘randomized controlled’:ti,ab,tt)) OR (‘systematic review’:ti,tt NOT (trial:ti,tt OR study:ti,tt)) OR (nonrandom*:ti,ab,tt NOT random*:ti,ab,tt) OR ‘random field*’:ti,ab,tt OR (‘random cluster’ NEAR/4 sampl*):ti,ab,tt OR (review:ab AND review:it) NOT trial:ti,tt OR (‘we searched’:ab AND (review:ti,tt OR review:it)) OR ‘update review’:ab OR (databases NEAR/5 searched):ab OR ((rat:ti,tt OR rats:ti,tt OR mouse:ti,tt OR mice:ti,tt OR swine:ti,tt OR porcine:ti,tt OR murine:ti,tt OR sheep:ti,tt OR lambs:ti,tt OR pigs:ti,tt OR piglets:ti,tt OR rabbit:ti,tt OR rabbits:ti,tt OR cat:ti,tt OR cats:ti,tt OR dog:ti,tt OR dogs:ti,tt OR cattle:ti,tt OR bovine:ti,tt OR monkey:ti,tt OR monkeys:ti,tt OR trout:ti,tt OR marmoset*:ti,tt) AND ‘animal experiment’/de) OR (‘animal experiment’/de NOT (‘human experiment’/de OR ‘human’/de))) | #4 |
| **Interventions** |  |
| Same as Pubmed searched as title, abstract, keyword, author | #5 |
| #3 AND #5 AND #4 | #6 |
| NOT [medline]/lim | #7 |
| Limit #7 to english language | #9 |

## **Supplementary Table S1.3. CENTRAL**

| **SEARCH** | **Number** |
| --- | --- |
| **Disease terms** |  |
| MeSH descriptor: [Depressive Disorder, Treatment-Resistant] explode all trees | #1 |
| (treatment-resistan* OR therapy-resistan* OR refract* OR resistan* OR nonrespon* OR unrespon* OR fail* OR “incomplete response” OR “no respon*” OR “inadequate response” OR (sub* NEAR/2 respon*) OR (poor* NEAR/3 respon*) OR “residual symptoms” OR “drug resistan*” OR “treatment failure” OR “medication resistan*” OR medication-resistan*) AND depress* | #2 |
| #1 OR #2 | #3 |
| **Interventions** |  |
| Same as Pubmed search (spelling of 6-Azamianserin to changed to “6 Azamianserin”) | #4 |
| #3 AND #4 | #5 |
| #5 NOT adolescent NOT child NOT infant (as MeSH descriptors) | #6 |

# 2. Demographics

## **Supplementary Table S2. Characteristics of included studies**

| **Study name** | **Treatment** | **Duration** | **N** | **% Female** | **Age (SD)** | **Baseline score (SD)** | **Scale** | **Comorbid axis I disorder** | **Additional medication** | **Previous antidepressants** | **Number previous antidepressants (SD)** | **Duration current episode (SD)** | **Placebo** | **Blinding** | **Sponsorship** |
| --- | --- | --- | --- | --- | --- | --- | --- | --- | --- | --- | --- | --- | --- | --- | --- |
| Berman et al. 2009 (1) | Aripiprazole | 6 weeks | 177 | 0.78 | 45.1 (10.6) | 19.8 (5) | HAMD17 | * | yes | ≥2 | * | 18.8 (*) | yes | yes | commercial |
|  | Placebo |  | 172 | 0.68 | 45.6 (11.3) | 20 (5) |  |  |  |  | * | 17.2 (*) |  |  |  |
| Berman et al. 2007 (2) | Aripiprazole | 6 weeks | 182 | 0.62 | 46.5 (10.6) | 26 (6.1) | MADRS | no | yes | ≥2 | * | 38.6 (59) | yes | yes | commercial |
|  | Placebo |  | 176 | 0.64 | 44.2 (10.9) | 25.9 (6.5) |  |  |  |  | * | 43.6 (53.8) |  |  |  |
| Kamijma et al. 2018 (3) | Aripiprazole | 6 weeks | 208 | 0.38 | 38.3 (11.8) | 24.9 (6.6) | MADRS | no | yes | ≥2 | * | 11.3 (14.3) | yes | yes | commercial |
|  | Placebo |  | 203 | 0.36 | 39.5 (11.8) | 25.2 (6.5) |  |  |  |  | * | 15.8 (35.6) |  |  |  |
| Kamijma et al. 2013 (4) | Aripiprazole | 6 weeks | 194 | 0.48 | 38.1 (9.6) | 25.3 (7.3) | MADRS | no | yes | ≥2 | 2.5 (*) | 17.5 (26.1) | yes | yes | commercial |
|  | Placebo |  | 195 | 0.41 | 38.7 (9.2) | 25.5 (7.4) |  |  |  |  | 2.5 (*) | 15.6 (16.4) |  |  |  |
| Marcus et al. 2008 (5) | Aripiprazole | 6 weeks | 191 | 0.66 | 44.6 (11) | 25.2 (6.2) | MADRS | no | yes | ≥2 | 2.3 (*) | 43.7 (68) | yes | yes | commercial |
|  | Placebo |  | 190 | 0.67 | 44.4 (10.7) | 27 (5.5) |  |  |  |  | 2.4 (*) | 48.5 (88.8) |  |  |  |
| Otsuka Pharmaceutical 2021 (6) | Aripiprazole | 6 weeks | 16 | * | * | * | MADRS | * | yes | ≥2 | * | * | yes | yes | commercial |
|  | Aripiprazole Switching |  | 15 | * | * | * |  |  | no |  | * | * |  |  |  |
|  | Placebo |  | 14 | * | * | * |  |  | no |  | * | * |  |  |  |
| Palhano-Fontes et al. 2019 (7) | Ayahuasca | 1 week | 17 | 0.79 | 39.7 (11.3) | 36.1 (6.1) | MADRS | yes | no | ≥2 | 3.9 (1.4) | 14.7 (18.9) | yes | yes | non-commercial |
|  | Placebo |  | 18 | 0.67 | 44.2 (12) | 30.1 (5.6) |  |  |  |  | 3.8 (1.9) | 10.1 (9.2) |  |  |  |
| Bauer et al. 2018 (8) | Brexpiprazole | 24 weeks | 442 | 0.69 | 47.1 (12.1) | 25.9 (4.1) | MADRS | no | yes | ≥2 | * | 8.6 (15) | yes | yes | commercial |
|  | Placebo |  | 444 | 0.69 | 46.4 (12.1) | 25.8 (4.1) |  |  |  |  | * | 8.9 (13.2) |  |  |  |
| Hobart et al. 2018a (9) | Brexpiprazole | 6 weeks | 192 | 0.77 | 43 (12.7) | 27.1 (5.7) | MADRS | no | yes | ≥2 | * | 13.3 (14.2) | yes | yes | commercial |
|  | Placebo |  | 202 | 0.71 | 42.7 (12.5) | 26.2 (6.2) |  |  |  |  | * | 19.4 (46.8) |  |  |  |
| Thase et al. 2015b (10) | Brexpiprazole | 6 weeks | 188 | 0.69 | 44.1 (11.6) | 26.6 (5.8) | MADRS | * | yes | ≥2 | * | 13.5 (14.2) | yes | yes | commercial |
|  | Placebo |  | 191 | 0.72 | 45.2 (11.3) | 27.1 (5.6) |  |  |  |  | * | 13.7 (17.1) |  |  |  |
| Thase et al. 2015a (11) | Brexpiprazole | 6 weeks | 230 | 0.68 | 44.5 (11.2) | 26.4 (5.2) | MADRS | * | yes | ≥2 | 2.2 (*) | 17.4 (33) | yes | yes | commercial |
|  | Placebo |  | 221 | 0.66 | 46.6 (11) | 26.3 (5.3) |  |  |  |  | 2.2 (*) | 16.9 (35) |  |  |  |
| Hobart et al. 2018b (12) | Brexpiprazole | 6 weeks | 197 | 0.65 | 43.6 (11.5) | 25.4 (5.1) | MADRS | no | yes | ≥2 | 2.2 (0.4) | 14.3 (19.1) | yes | yes | commercial |
|  | Quetiapine XR |  | 100 | 0.66 | 44.6 (11.6) | 25.6 (5.5) |  |  |  |  | 2.2 (0.4) | 12.3 (19) |  |  |  |
|  | Placebo |  | 206 | 0.72 | 41.8 (11.7) | 25.4 (5.2) |  |  |  |  | 2.3 (0.5) | 11.7 (14.6) |  |  |  |
| Heresco-Levy et al. 2013 (13) | D-Cycloserine | 6 weeks | 13 | * | * | 25.1 (5.6) | HAMD21 | * | yes | ≥2 | * | * | yes | yes | non-commercial |
|  | Placebo |  | 13 | * | * | 27.2 (4.9) |  |  |  |  | * | * |  |  |  |
| Dougherty et al. 2015 (14) | DBS | 16 weeks | 16 | 0.50 | 46.6 (14.4) | 37 (5.1) | MADRS | * | yes | ≥4 | * | 133.2 (88.8) | yes | yes | commercial |
|  | Placebo |  | 14 | 0.36 | 48.9 (8.9) | 36.4 (3.3) |  |  |  |  | * | 141.6 (67.2) |  |  |  |
| Holtzheimer et al. 2017 (15) | DBS | 6 months | 60 | 0.50 | 50.5 (9.7) | 33.8 (4.5) | MADRS | no | yes | ≥4 | 7.4 (3.1) | 151.4 (97.8) | yes | yes | commercial |
|  | Placebo |  | 30 | 0.57 | 48.7 (0.6) | 37.3 (3.8) |  |  |  |  | 8.8 (4.4) | 116 (56) |  |  |  |
| Keshtkar et al. 2011 (16) | ECT | 10 days | 40 | 0.80 | 34 (9.9) | 25.8 (6.1) | HAMD21 | * | yes | ≥2 | * | * | no | no | non-commercial |
|  | rTMS | 22 days | 35 | 0.60 | 35.6 (8.1) | 21 (7.5) |  |  |  |  | * | * |  |  |  |
| Rosa et al. 2006 (17) | ECT | 4 weeks | 20 | 0.47 | 46 (10.6) | 32.1 (5) | HAMD17 | * | no | ≥2 | * | 10.5 (3) | no | no | non-commercial |
|  | rTMS |  | 22 | 0.60 | 41.8 (10.2) | 30.1 (4.7) |  |  |  |  | * | 11.5 (5.1) |  |  |  |
| Ramasubramanian et al. 2022 (18) | ECT | 2 weeks | 47 | 0.50 | * | 19 (1.9) | HAMD17 | * | yes | ≥2 | * | * | no | no | non-commercial |
|  | tDCS |  | 44 | 0.43 | * | 19.2 (2.1) |  |  |  |  | * | * |  |  |  |
| Eli Lilly and Company 2017 (19) | Olanzapine/Fluoxetine | 8 weeks | 88 | 0.47 | 38.6 (12.2) | 32.1 (5.3) | MADRS | * | * | ≥2 | * | * | no | yes | non-commercial |
|  | Fluoxetine |  | 88 | 0.41 | 41.5 (12..4) | 31.8 (4.8) |  |  |  |  | * | * |  |  |  |
| Thase et al. 2007 (20) | Olanzapine | 8 weeks | 200 | 0.66 | 44.3 (10.2) | 30.1 (6.7) | MADRS | * | yes | ≥2 | * | 13.7 (18.1) | yes | yes | commercial |
|  | Placebo |  | 206 | 0.62 | 44.6 (10) | 29.9 (6.4) |  |  |  |  | * | 14.1 (19.8) |  |  |  |
| Singh et al. 2016b (21) | Ketamine | 24 hours | 11 | 0.64 | 41.8 (11.6) | 33.7 (5.8) | MADRS | * | * | ≥2 | * | * | yes | yes | commercial |
|  | Placebo |  | 10 | 0.60 | 42.7 (10.9) | 33.9 (4.2) |  |  |  |  | * | * |  |  |  |
| Ahmed et al. 2023 (22) | Ketamine | 3 weeks | 18 | 0.44 | 36.1 (13.8) | 28.3 (4.5) | HAMD17 | yes | yes | ≥2 | 5.1 (1.2) | * | yes | yes | non-commercial |
|  | Placebo |  | 18 | 0.39 | 36.6 (13.7) | 31.2 (5.3) |  |  |  |  | 4.9 (0.9) | * |  |  |  |
| Chen et al. 2018 (23) | Ketamine | 2 weeks | 24 | 0.88 | 48.5 (11) | 23 (4.9) | HAMD17 | * | * | ≥3 | * | * | yes | yes | non-commercial |
|  | Placebo |  | 24 | 0.63 | 48.6 (8.1) | 23.3 (4.1) |  |  |  |  | * | * |  |  |  |
| Fava et al. 2020 (24) | Ketamine | 3 days | 22 | 0.50 | 48.6 (12.9) | 31.6 (3.9) | MADRS | * | yes | ≥2 | * | * | yes | yes | non-commercial |
|  | Ketamine |  | 20 | 0.40 | 47.4 (10.1) | 32.7 (5.9) |  |  |  |  | * | * |  |  |  |
|  | Placebo |  | 18 | 0.58 | 45.6 (13.8) | 33.6 (7.1) |  |  |  |  | * | * |  |  |  |
| Ionescu et al. 2019 (25) | Ketamine | 3weeks | 13 | 0.54 | 45.5 (13.6) | 31.6 (5.2) | HAMD26 | * | yes | ≥3 | 6.6 (2.9) | 132.5 (154.6) | yes | yes | non-commercial |
|  | Placebo |  | 13 | 0.23 | 45.3 (11.7) | 26.3 (4.8) |  |  |  |  | 8.2 (3.1) | 91.6 (126.4) |  |  |  |
| Li et al. 2016 (26) | Ketamine | 4 hours | 16 | 0.69 | 43.4 (11.9) | 22.6 (5.8) | HAMD17 | yes | yes | ≥3 | * | * | yes | yes | non-commercial |
|  | Placebo |  | 16 | 0.81 | 49.9 (8.1) | 22.8 (3.9) |  |  |  |  | * | * |  |  |  |
| Murrough et al. 2013 (27) | Ketamine | 24 hours | 48 | 0.55 | 46.9 (12.8) | 32.6 (6.1) | MADRS | * | no | ≥3 | 5.1 (2) | * | yes | yes | non-commercial |
|  | Placebo |  | 25 | 0.44 | 42.7 (11.6) | 31.1 (5.6) |  |  |  |  | 5 (1.8) | * |  |  |  |
| Shiroma et al. 2020 (28) | Ketamine | 10 days | 28 | 0.12 | 54.4 (13.8) | * | MADRS | yes | yes | ≥2 | 4.6 (1.9) | 18.2 (2) | yes | yes | non-commercial |
|  | Placebo |  | 30 | 0.17 | 51.2 (12.5) | * |  |  |  |  | * | * |  |  |  |
| Singh et al. 2016a (29) | Ketamine | 15 days | 18 | 0.67 | 45.7 (9.6) | 33.3 (4.9) | MADRS | * | yes | ≥2 | * | * | yes | yes | commercial |
|  | Placebo |  | 17 | 0.75 | 40.3 (11.8) | 35.6 (3.8) |  |  |  |  | * | * |  |  |  |
|  | Ketamine |  | 17 | 0.71 | 43.3 (12) | 35.4 (5.3) | MADRS |  |  | ≥2 | * | * |  |  |  |
|  | Placebo |  | 16 | 0.56 | 46.1 (10.5) | 36.8 (5.8) |  |  |  |  | * | * |  |  |  |
| Su et al. 2023 (30) | Ketamine | 24h | 42 | 0.67 | 34.3 (13.3) | 35.8 (4.5) | MADRS | yes | yes | ≥2 | * | * | yes | yes | non-commercial |
|  | Placebo |  | 42 | 0.74 | 36.9 (12.2) | 38.3 (3.8) |  |  |  |  | * | * |  |  |  |
| Daly et al. 2017 (31) | Ketamine | 8 days | 11 | 0.82 | 42.7 (11.2) | 33.2 (6.3) | MADRS | * | yes | ≥2 | * | 9.2 (5) | yes | yes | commercial |
|  | Ketamine |  | 12 | 0.50 | 49.8 (9.3) | 35 (4.2) |  |  |  |  | * | 15.3 (11.9) |  |  |  |
|  | Placebo |  | 33 | 0.55 | 44.4 (9.6) | 35 (5.2) |  |  |  |  | * | 15 (18.4) |  |  |  |
| Takahashi et al. 2021 (32) | Ketamine | 4 weeks | 40 | 0.40 | 42.5 (8.4) | 37.9 (5.4) | MADRS | * | yes | ≥2 | * | 12.8 (10.8) | yes | yes | commercial |
|  | Ketamine |  | 41 | 0.44 | 41.9 (10.3) | 35.9 (5.3) |  |  |  |  | * | 15 (22.8) |  |  |  |
|  | Placebo |  | 80 | 0.49 | 43.3 (11.4) | 37.7 (5.7) |  |  |  |  | * | 14.1 (27.4) |  |  |  |
| Domany et al. 2019 (33) | Ketamine | 3 weeks | 22 | 0.23 | 38.7 (13.3) | 33.4 (5.5) | MADRS | yes | yes | ≥2 | * | * | yes | yes | non-commercial |
|  | Placebo |  | 19 | 0.56 | 37.9 (13.4) | 30 (7.4) |  |  |  |  | * | * |  |  |  |
| Barbee et al. 2011 (34) | Lamotrigine | 10 weeks | 48 | 0.69 | 44.6 (12.2) | 27.4 (6.6) | MADRS | yes | yes | ≥2 | * | 24.6 (33.3) | yes | yes | commercial |
|  | Placebo |  | 48 | 0.69 | 45.8 (11) | 26.6 (4.9) |  |  |  |  | * | 29.3 (40.2) |  |  |  |
| Santos et al. 2008 (35) | Lamotrigine | 8 weeks | 17 | 0.82 | 26 (*) | 32.3 (7.8) | MADRS | * | yes | ≥2 | * | 20 (21) | yes | yes | non-commercial |
|  | Placebo |  | 17 | 0.65 | 29 (*) | 28.4 (7.7) |  |  |  |  | * | 44.5 (66.1) |  |  |  |
| Schindler et al. 2007 (36) | Lamotrigine | 8 weeks | 17 | 0.53 | 45.1 (13.4) | 22.7 (3.9) | HAMD17 | yes | yes | ≥2 | * | 6.9 (2.6) | no | no | * |
|  | Lithium |  | 17 | 0.47 | 50.3 (13.6) | 21.5 (3.8) |  |  |  |  | * | 7.8 (2.6) |  |  |  |
| Sanacora et al. 2014 (37) | Lanicemine | 3 weeks | 102 | 0.71 | 46.3 (9.7) | 33.7 (5.3) | MADRS | yes | yes | ≥2 | * | * | yes | yes | commercial |
|  | Placebo |  | 50 | 0.70 | 44.4 (10.1) | 33.5 (4.5) |  |  |  |  | * | * |  |  |  |
| Sanacora et al. 2017 (38) | Lanicemine | 6 weeks | 201 | 0.65 | 47.6 (11.5) | 36.3 (4.7) | MADRS | yes | yes | ≥3 | * | * | yes | yes | commercial |
|  | Placebo |  | 100 | 0.65 | 49.5 (11.1) | 35.6 (4.8) |  |  |  |  | * | * |  |  |  |
| Nierenberg et al. 2003 (39) | Lithium | 6 weeks | 18 | 0.50 | 37.2 (8.3) | * | HAMD17 | yes | yes | ≥2 | * | 97.3 (111.8) | yes | yes | non-commercial |
|  | Placebo |  | 17 | 0.41 | 39.7 (11.9) | * |  |  |  |  | * | 84.5 (94.9) |  |  |  |
| Nierenberg et al. 2006 (40) | Lithium | 12 weeks | 69 | 0.61 | 40.6 (12.2) | 19 (6.6) | HAMD17 | yes | yes | ≥2 | * | 29.1 (80.9) | no | no | non-commercial |
|  | Thyroid Hormone |  | 73 | 0.56 | 43.2 (11.8) | 17.2 (6.2) |  |  |  |  | * | 29.9 (67.8) |  |  |  |
| Husain et al. 2017 (41) | Minocycline | 12 weeks | 21 | 0.45 | * | 34.5 (10.9) | HAMD17 | * | yes | ≥2 | * | * | yes | yes | non-commercial |
|  | Placebo |  | 20 | 0.55 | * | 32.6 (10.1) |  |  |  |  | * | * |  |  |  |
| Nagele et al. 2015 (42) | Nitrous Oxide | 24 hours | 10 | * | * | 23 (7.4) | HAMD21 | * | yes | ≥3 | * | * | yes | yes | non-commercial |
|  | Placebo |  | 10 | * | * | 25.5 (10.3) |  |  |  |  | * | * |  |  |  |
| Yan et al. 2022 (43) | Nitrous Oxide | 24 hours | 20 | 0.45 | 34 (*) | 21.3 (3.7) | HAMD17 | * | yes | ≥2 | * | 5 (*) | yes | yes | non-commercial |
|  | Placebo |  | 22 | 0.64 | 28 (*) | 21.3 (3.6) |  |  |  |  | * | 4 (*) |  |  |  |
| Corya et al. 2006 (44) | Olanzapine/Fluoxetine | 12 weeks | 243 | * | * | * | MADRS | * | no | ≥2 | * | * | yes | yes | commercial |
|  | Olanzapine |  | 62 | * | * | * |  |  | no |  | * | * |  |  |  |
|  | Fluoxetine |  | 60 | * | * | * |  |  | no |  | * | * |  |  |  |
|  | Placebo |  | 59 | * | * | * |  |  | yes |  | * | * |  |  |  |
| Shelton et al 2005 (45) | Olanzapine/Fluoxetine | 8 weeks | 146 | 0.67 | 42.5 (10.7) | 28.5 (7.5) | MADRS | * | no | ≥2 | * | * | yes | yes | commercial |
|  | Olanzapine |  | 144 | 0.65 | 43.4 (11) | 28.4 (7.3) |  |  | no |  | * | * |  |  |  |
|  | Fluoxetine |  | 142 | 0.73 | 41.7 (11) | 28.4 (7.3) |  |  | no |  | * | * |  |  |  |
|  | Placebo |  | 68 | 0.68 | 41.5 (10.1) | 28.8 (6.5) |  |  | yes |  | * | * |  |  |  |
| Goodwin et al. 2022 (46) | Psilocybin 25mg | 3 weeks | 154 | 0.55 | 40.4 (12.5) | 32.4 (5.9) | MADRS | * | no | 2 - 4 | * | * | yes | yes | commercial |
|  | Placebo |  | 79 | 0.46 | 38.7 (11.7) | 32.7 (6.2) |  |  |  |  | * | * |  |  |  |
| Astellas Pharma Inc. 2014 (47) | Quetiapine XR | 6 weeks | 43 | 0.40 | 37.3 (9.7) | 29.3 (5.4) | MADRS | no | yes | ≥2 | * | 9 (6.3) | yes | yes | commercial |
|  | Placebo |  | 44 | 0.43 | 39.8 (11.2) | 28.5 (5.1) |  |  |  |  | * | 7.6 (6.2) |  |  |  |
| Bauer et al. 2013 (48) | Quetiapine XR Mono | 6 weeks | 112 | * | * | * | MADRS | no | no | ≥2 | * | * | no | no | commercial |
|  | Quetiapine XR |  | 114 | * | * | * |  |  | yes |  | * | * |  |  |  |
|  | Lithium |  | 110 | * | * | * |  |  | yes |  | * | * |  |  |  |
| Akpinar et al. 2022 (49) | rTMS | 2 weeks | 20 | 0.85 | 43.7 (14.2) | 20.2 (3.3) | HAMD17 | yes | yes | ≥2 | * | 5.1 (3.2) | yes | yes | non-commercial |
|  | Placebo |  | 18 | 0.83 | 45.6 (7.8) | 20.5 (3.4) |  |  |  |  | * | 5.3 (2.8) |  |  |  |
| Avery et al. 2006 (50) | rTMS | 4 weeks | 35 | 0.60 | 44.3 (10.3) | 23.5 (3.9) | HAMD17 | * | yes | ≥2 | 3.2 (2.4) | 28.1 (16.4) | yes | yes | non-commercial |
|  | Placebo |  | 33 | 0.52 | 44.2 (9.7) | 23.5 (2.9) |  |  |  |  | 3.3 (1.7) | 26.3 (16.9) |  |  |  |
| Bakim et al. 2012 (51) | rTMS | 6 weeks | 12 | 0.83 | 38.8 (10) | 27.2 (4.9) | MADRS | no | yes | ≥2 | 3.9 (2) | 16.8 (10.8) | yes | yes | non-commercial |
|  | rTMS |  | 11 | 0.91 | 43.1 (8.2) | 27.8 (3.1) |  |  |  | ≥2 | 3.4 (1.2) | 17.7 (10.3) |  |  |  |
|  | Placebo |  | 12 | 0.92 | 44.4 (10.2) | 28.8 (5.6) |  |  |  | ≥2 | 3.3 (1.1) | 17.7 (9.5) |  |  |  |
| Blumberg et al. 2016 (52) | rTMS | 3weeks | 40 | 0.58 | 46.4 (12.5) | 24.1 (3.2) | HAMD17 | yes | yes | ≥2 | * | 51.5 (70.5) | yes | yes | non-commercial |
|  | rTMS |  | 40 | 0.75 | 46.5 (14.1) | 26 (3.4) |  |  |  |  | * | 30.9 (64.1) |  |  |  |
|  | Placebo |  | 41 | 0.59 | 48.1 (12) | 25.5 (3.6) |  |  |  |  | * | 46.9 (110.3) |  |  |  |
| Blumberg et al. 2012a (53) | rTMS | 3 or 6 weeks | 28 | 0.54 | 58 (12.5) | 25.1 (3.8) | HAMD17 | yes | yes | ≥2 | * | * | yes | yes | non-commercial |
|  | rTMS |  | 22 | 0.55 | 48.9 (13.4) | 26 (3.3) |  |  |  |  | * | * |  |  |  |
|  | Placebo |  | 20 | 0.70 | 45.8 (13.4) | 25.2 (2.8) |  |  |  |  | * | * |  |  |  |
| Chen et al. 2013 (54) | rTMS | 6 weeks | 10 | 0.70 | 44.1 (4.4) | 23.5 (1.9) | HAMD17 | * | yes | ≥2 | * | * | yes | yes | non-commercial |
|  | Placebo |  | 11 | 0.40 | 47.3 (3.5) | 24.9 (1.9) |  |  |  |  | * | * |  |  |  |
| Fitzgerald et al. 2012 (55) | rTMS | 3 weeks | 22 | 0.64 | 40.5 (15.5) | 33.6 (6.4) | MADRS | yes | yes | ≥2 | 4.7 (3.1) | * | yes | yes | non-commercial |
|  | rTMS |  | 24 | 0.63 | 43.4 (12.7) | 32 (4.6) |  |  |  |  | 5.5 (3.7) | * |  |  |  |
|  | Placebo |  | 20 | 0.40 | 44.9 (15.7) | 32 (3.5) |  |  |  |  | 4.9 (2.6) | * |  |  |  |
| Garcia-Toro et al. 2006 (56) | rTMS | 2 weeks | 10 | 0.40 | 48.5 (13.3) | 27.3 (5) | HAMD21 | * | yes | ≥2 | * | 35.7 (26.4) | yes | yes | non-commercial |
|  | rTMS |  | 10 | 0.40 | 51.1 (13.8) | 25 (4.1) |  |  |  |  | * | 29.6 (21.4) |  |  |  |
|  | Placebo |  | 10 | 0.60 | 47.2 (11.8) | 25.1 (7.3) |  |  |  |  | * | 32.6 (26.4) |  |  |  |
| Garcia-Toro et al. 2001 (57) | rTMS | 2 weeks | 17 | 0.41 | 51.5 (15.9) | 27.1 (6.7) | HAMD21 | * | yes | ≥2 | * | * | yes | yes | non-commercial |
|  | Placebo |  | 18 | 0.44 | 50 (11) | 25.6 (4.9) |  |  |  |  | * | * |  |  |  |
| Pallanti et al. 2010 (58) | rTMS | 3 weeks | 20 | 0.55 | 47.6 (12.3) | 28.8 (6) | HAMD17 | * | yes | ≥2 | 5.9 (1.5) | 9.2 (2.7) | yes | yes | non-commercial |
|  | rTMS |  | 20 | 0.60 | 51.2 (12.5) | 28 (5.9) |  |  |  |  | 6.5 (1.5) | 9.7 (2.7) |  |  |  |
|  | Placebo |  | 20 | 0.60 | 47.9 (9.1) | 29.1 (3.5) |  |  |  |  | 6 (1.7) | 8.7 (2) |  |  |  |
| Theleritis et al. 2017 (59) | rTMS | 3 weeks | 27 | 0.56 | 39.1 (10.1) | 30.6 (3.2) | HAMD17 | no | yes | ≥2 | * | * | yes | yes | non-commercial |
|  | rTMS |  | 27 | 0.42 | 38.9 (13.9) | 29.7 (4.6) |  |  |  |  | * | * |  |  |  |
|  | Placebo |  | 20 | 0.52 | 38 (9.9) | 29.4 (3.2) |  |  |  |  | * | * |  |  |  |
|  | Placebo |  | 24 | 0.42 | 39.4 (8.9) | 30.3 (3.6) |  |  |  |  | * | * |  |  |  |
| Triggs et al. 2010 (60) | rTMS | 2 weeks | 18 | 0.78 | 46.7 (15.3) | 28.2 (6) | HAMD24 | * | yes | ≥2 | * | * | yes | yes | non-commercial |
|  | Placebo |  | 14 | 0.43 | 44.3 (16.9) | 27.5 (3) |  |  |  |  | * | * |  |  |  |
| van Eijndhoven et al. 2020 (61) | rTMS | 5 weeks | 15 | 0.60 | 47.3 (11.5) | 24.1 (4.2) | HAMD17 | * | yes | ≥2 | * | 54.6 (26.2) | yes | yes | non-commercial |
|  | Placebo |  | 16 | 0.81 | 49.7 (11) | 22.7 (3.8) |  |  |  |  | * | 57.9 (54.8) |  |  |  |
| Zheng et al. 2010 (62) | rTMS | 4 weeks | 19 | 0.37 | 26.9 (6.2) | 24.6 (3) | HAMD17 | no | yes | ≥2 | * | * | yes | yes | non-commercial |
|  | Placebo |  | 15 | 0.33 | 26.7 (4.3) | 24.6 (2.8) |  |  |  |  | * | * |  |  |  |
| Blumberger et al. 2018 (67) | rTMS | 5 weeks | 205 | 0.58 | 43.2 (12.2) | 23.6 (4.4) | HAMD17 | * | yes | ≥2 | * | 23.9 (28.8) | no | no | commercial |
|  | TBS |  | 209 | 0.61 | 41.6 (10.8) | 23.7 (4.4) |  |  |  |  | * | 22.8 (25.7) |  |  |  |
| Bulteau et al. 2022 (68) | rTMS | 4 weeks | 30 | 0.60 | 48.5 (14.7) | 27.3 (3.3) | MADRS | * | yes | ≥2 | 3.4 (1.6) | 20 (*) | no | yes | non-commercial |
|  | TBS |  | 30 | 0.78 | 56.1 (10.9) | 29.8 (6.6) |  |  |  |  | 3.9 (2.2) | 19 (*) |  |  |  |
| Bennabi et al. 2015 (63) | tDCS | 5 days | 12 | 0.83 | 60.4 (12) | 29.2 (4) | MADRS | * | yes | ≥2 | * | * | yes | yes | non-commercial |
|  | Placebo |  | 11 | 0.45 | 59.9 (15.4) | 33.5 (7.5) |  |  |  |  | * | * |  |  |  |
| Blumberger et al. 2012b (64) | tDCS | 3 weeks | 13 | 0.77 | 45.3 (11.6) | 31.5 (5.8) | MADRS | yes | yes | ≥2 | 4.3 (2.4) | 51.6 (67.2) | yes | yes | non-commercial |
|  | Placebo |  | 11 | 0.91 | 49.7 (9.4) | 32 (7) |  |  |  |  | 4.1 (2.2) | 40.8 (36) |  |  |  |
| Cole et al. 2022 (65) | TBS | 5 days | 14 | 0.36 | 49 (15) | 31 (4) | MADRS | no | yes | moderate Maudsley Staging Method | 5 (2) | 96 (168) | yes | yes | non-commercial |
|  | Placebo |  | 15 | 0.33 | 52 (16) | 35 (6) |  |  |  |  | 5 (2) | 120 (156) |  |  |  |
| Li et al. 2014 (66) | TBS | 2 weeks | 15 | 0.67 | 49.2 (*) | 24.3 (5.5) | HAMD17 | yes | yes | moderate Maudsley Staging Method | * | * | yes | yes | non-commercial |
|  | TBS |  | 15 | 0.53 | 42.4 (*) | 23.1 (3.9) |  |  |  |  | * | * |  |  |  |
|  | TBS |  | 15 | 0.73 | 42.5 (*) | 25.4 (5.1) |  |  |  |  | * | * |  |  |  |
|  | Placebo |  | 15 | 0.73 | 46.9 (*) | 23.8 (3.2) |  |  |  |  | * | * |  |  |  |
| Fang et al. 2011 (69) | Risperidone | 8 weeks | 45 | * | * | * | HAMD17 | * | yes | ≥2 | * | * | no | yes | non-commercial |
|  | Sodium Valproate |  | 39 | * | * | * |  |  |  |  | * | * |  |  |  |
|  | Buspirone |  | 46 | * | * | * |  |  |  |  | * | * |  |  |  |
|  | Thyroid Hormone |  | 48 | * | * | * |  |  |  |  | * | * |  |  |  |

*: no information was provided.

Abbreviations: DBS: deep brain stimulation; ECT: electroconvulsive therapy; HAM-D: Hamilton Depression Rating Scale; MADRS: Montgomery-Asberg Depression Rating Scale; rTMS: repetitive transcranial magnetic therapy; TBS: theta burst stimulation; tDCS: transcranial direct current stimulation; SD: standard deviation; XR: extended release.

## **Supplementary Figure S2.1. Depression severity by treatment**


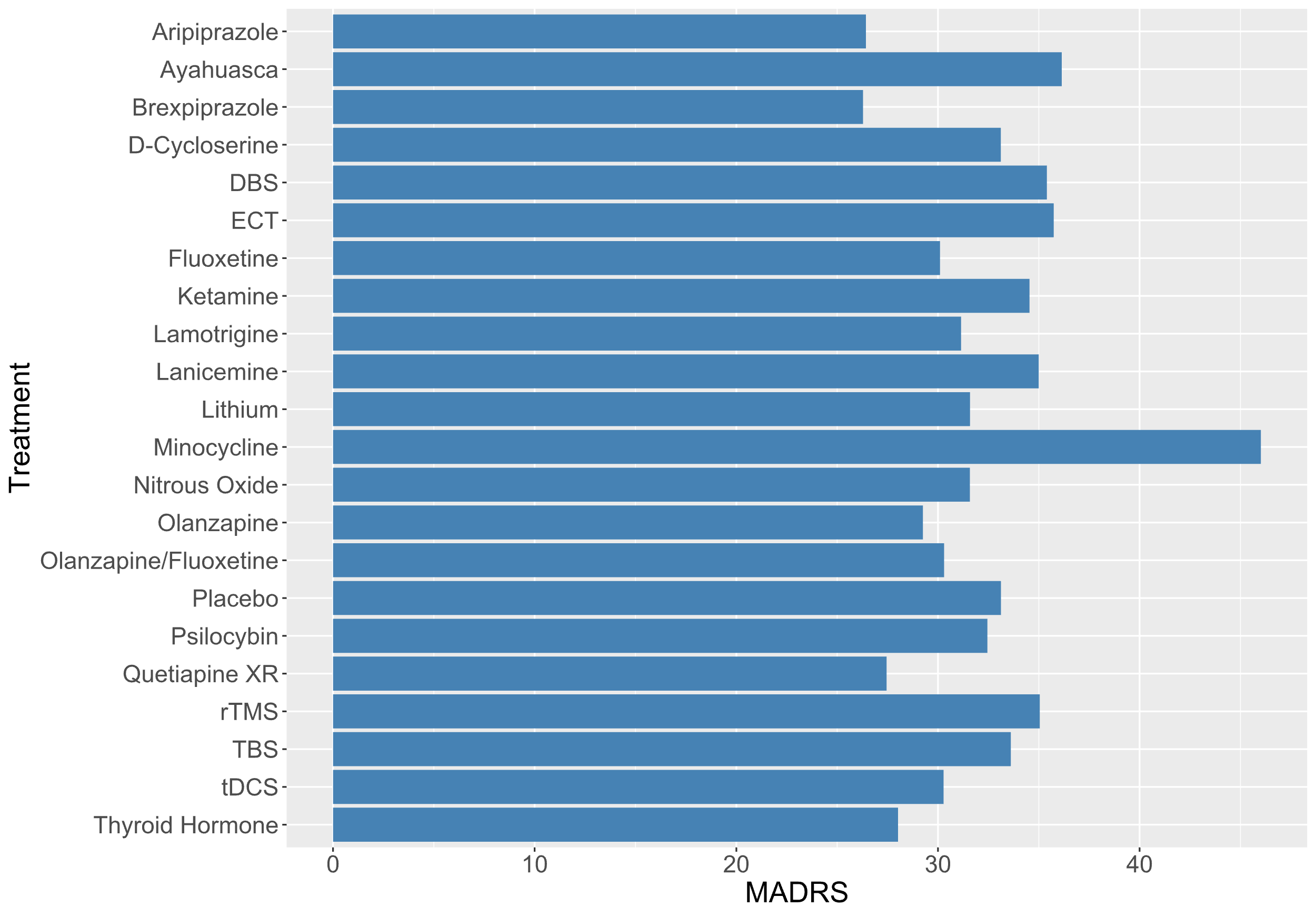


Abbreviations: DBS: deep brain stimulation; ECT: electroconvulsive therapy; rTMS: repetitive transcranial magnetic therapy; TBS: theta burst stimulation; tDCS: transcranial direct current stimulation; XR: extended release

## **Supplementary Figure S2.2. Duration of study (in weeks) by treatment**


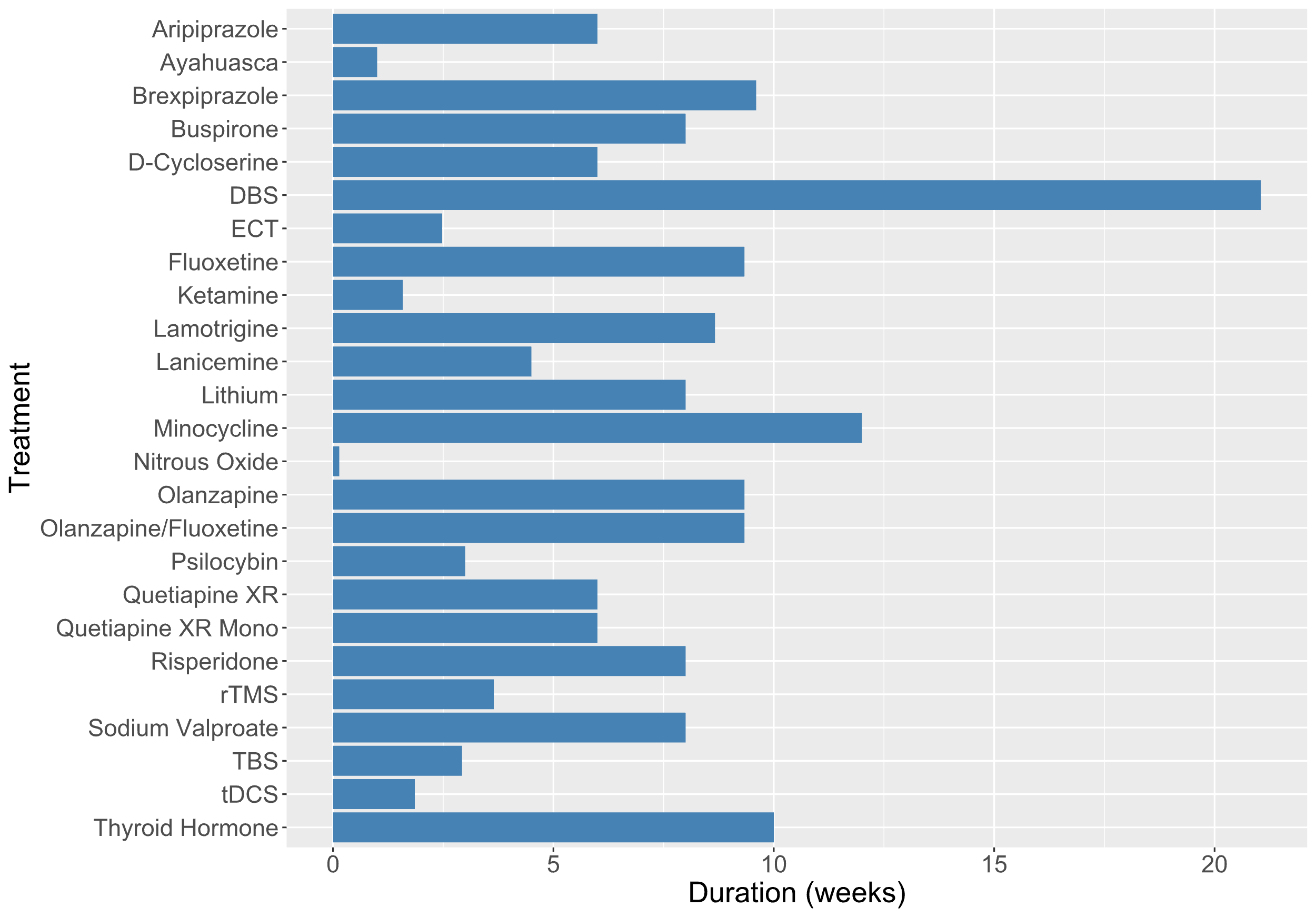


Abbreviations: DBS: deep brain stimulation; ECT: electroconvulsive therapy; rTMS: repetitive transcranial magnetic therapy; TBS: theta burst stimulation; tDCS: transcranial direct current stimulation; XR: extended release

# 3. Risk of bias assessment

## **Supplementary Table S3. Risk of bias of included studies**

| **Study** | **Randomization** | **Adhering to intervention** | **Missing outcome data** | **Measurement of outcome** | **Selection of reported results** | **Overall** |
| --- | --- | --- | --- | --- | --- | --- |
| Astellas Pharma Inc. 2014 (47) | Some concerns | Low Risk | Low Risk | Low Risk | Low Risk | Some concerns |
| Avery et al. 2006 (50) | Low Risk | Low Risk | Low Risk | Low Risk | Some concerns | Some concerns |
| Bakim et al., 2012 (51) | Some concerns | Some concerns | Low Risk | Low Risk | Some concerns | Some concerns |
| Barbee et al. 2011 (34) | Low Risk | Low Risk | Low Risk | Low Risk | Low Risk | Low Risk |
| Bauer et al. 2013 (48) | Low Risk | Some concerns | Low Risk | Low Risk | Low Risk | Some concerns |
| Bauer et al. 2018 (8) | Some concerns | Some concerns | Low Risk | Low Risk | Some concerns | Some concerns |
| Bennabi et al. 2015 (63) | Low Risk | High Risk | High Risk | Low Risk | Low Risk | High Risk |
| Berman et al. 2007 (2) | Some concerns | Some concerns | Low Risk | Low Risk | Low Risk | Some concerns |
| Berman et al. 2009 (1) | Some concerns | Some concerns | Low Risk | Low Risk | Low Risk | Some concerns |
| Blumberger et al. 2012a (53) | Low Risk | Low Risk | Low Risk | Low Risk | Low Risk | Low Risk |
| Blumberger et al. 2012b (64) | Low Risk | Low Risk | Low Risk | Low Risk | Low Risk | Low Risk |
| Blumberger et al. 2016 (52) | Some concerns | Low Risk | Low Risk | Low Risk | Low Risk | Some concerns |
| Blumberger et al. 2018 (67) | Low Risk | Some concerns | Low Risk | Low Risk | Low Risk | Some concerns |
| Chen et al., 2013 (54) | Some concerns | Some concerns | Low Risk | Low Risk | Some concerns | Some concerns |
| Chen et al., 2018 (23) | Some concerns | Low Risk | Low Risk | Low Risk | Some concerns | Some concerns |
| Cole et al. 2022 (65) | Some concerns | Low Risk | Low Risk | Low Risk | Low Risk | Some concerns |
| Corya et al., 2006 (44) | Some concerns | Low Risk | Low Risk | Low Risk | Some concerns | Some concerns |
| Daly et al. 2017 (31) | Low Risk | Low Risk | Low Risk | Low Risk | Low Risk | Low Risk |
| Domany et al. 2019 (33) | Low Risk | Some concerns | Low Risk | Low Risk | High Risk | High Risk |
| Dougherty et al. 2015 (14) | Low Risk | Some concerns | Low Risk | Low Risk | Low Risk | Some concerns |
| Eli Lilly and Company, 2017 (19) | Some concerns | Low Risk | Low Risk | Low Risk | Low Risk | Some concerns |
| Fang et al. 2011 (69) | Low Risk | Low Risk | Low Risk | Low Risk | Some concerns | Some concerns |
| Fava et al. 2020 (24) | Some concerns | Some concerns | Low Risk | High Risk | Low Risk | High Risk |
| Fitzgerald et al. 2012 (55) | Some concerns | High Risk | Some concerns | Low Risk | Some concerns | High Risk |
| Garcia-Toro et al. 2001 (57) | Some concerns | High Risk | High Risk | Low Risk | Some concerns | High Risk |
| Garcia-Toro et al. 2006 (56) | Low Risk | Low Risk | Low Risk | Low Risk | Some concerns | Some concerns |
| Goodwin et al. 2022 (46) | Low Risk | Low Risk | Low Risk | Low Risk | Low Risk | Low Risk |
| Heresco-Levy et al. 2013 (13) | Some concerns | Low Risk | Low Risk | Low Risk | Low Risk | Some concerns |
| Hobart et al. 2018a (9) | Low Risk | Low Risk | Low Risk | Low Risk | Low Risk | Low Risk |
| Hobart et al. 2018b (12) | Low Risk | Low Risk | Low Risk | Low Risk | Low Risk | Low Risk |
| Holtzheimer et al. 2017 (15) | Low Risk | Low Risk | Some concerns | Low Risk | Some concerns | Some concerns |
| Husain et al. 2017 (41) | Low Risk | Low Risk | Low Risk | Low Risk | Low Risk | Low Risk |
| Ionescu et al. 2009 (25) | Low Risk | Low Risk | Low Risk | Low Risk | Some concerns | Some concerns |
| Kamijma et al. 2013 (4) | Some concerns | Low Risk | Low Risk | Low Risk | Low Risk | Some concerns |
| Kamijma et al. 2018 (3) | Low Risk | Low Risk | Low Risk | Low Risk | Low Risk | Low Risk |
| Keshtkar et al. 2011 (16) | High Risk | Some concerns | Some concerns | Some concerns | Some concerns | High Risk |
| Li et al. 2014 (66) | Some concerns | Low Risk | Low Risk | Low Risk | Some concerns | Some concerns |
| Li et al., 2016 (26) | Low Risk | Low Risk | Low Risk | Low Risk | Some concerns | Some concerns |
| Marcus et al., 2008 (5) | Some concerns | Low Risk | Low Risk | Low Risk | Some concerns | Some concerns |
| Murrough et al. 2013 (27) | Low Risk | Low Risk | Low Risk | Low Risk | Low Risk | Low Risk |
| Nagele et al. 2015 (42) | Low Risk | Low Risk | Low Risk | Low Risk | Low Risk | Low Risk |
| Nierenberg et al. 2003 (39) | Some concerns | Low Risk | Low Risk | Low Risk | Some concerns | Some concerns |
| Nierenberg et al. 2006 (40) | Low Risk | Low Risk | Low Risk | Low Risk | Low Risk | Low Risk |
| Otsuka Pharma, 2021 (6) | Some concerns | Low Risk | Low Risk | Low Risk | Low Risk | Some concerns |
| Palhano-Fontes et al. 2019 (7) | Some concerns | Low Risk | Some concerns | Low Risk | Low Risk | Some concerns |
| Pallanti et al. 2010 (58) | Low Risk | Low Risk | Low Risk | Low Risk | Low Risk | Low Risk |
| Rosa et al., 2006 (17) | Some concerns | Low Risk | Some concerns | Low Risk | Some concerns | Some concerns |
| Sanacora et al. 2014 (37) | Some concerns | Low Risk | Low Risk | Low Risk | Low Risk | Some concerns |
| Sanacora et al. 2017 (38) | Low Risk | Some concerns | Low Risk | Low Risk | Low Risk | Some concerns |
| Santos et al., 2008 (35) | Low Risk | Some concerns | Some concerns | Low Risk | Low Risk | Some concerns |
| Schindler et al. 2007 (36) | Some concerns | Low Risk | Low Risk | Some concerns | Some concerns | Some concerns |
| Shelton et al, 2005 (45) | Some concerns | Low Risk | High Risk | Low Risk | Some concerns | High Risk |
| Shiroma et al. 2020 (28) | Some concerns | Low Risk | Some concerns | Low Risk | Some concerns | Some concerns |
| Singh et al., 2016a (29) | Low Risk | Low Risk | Low Risk | Low Risk | Low Risk | Low Risk |
| Singh et al., 2016b (21) | Low Risk | Low Risk | Low Risk | Low Risk | Low Risk | Low Risk |
| Takahashi et al. 2021 (32) | Some concerns | High Risk | High Risk | Low Risk | Low Risk | High Risk |
| Thase et al. 2007 (20) | Some concerns | Low Risk | Low Risk | Low Risk | Some concerns | Some concerns |
| Thase et al. 2015a (11) | Low Risk | Low Risk | Low Risk | Low Risk | Low Risk | Low Risk |
| Thase et al. 2015b (10) | Low Risk | Low Risk | Low Risk | Low Risk | Low Risk | Low Risk |
| Theleretis et al. 2017 (59) | Low Risk | Low Risk | Low Risk | Low Risk | Low Risk | Low Risk |
| Triggs et al. 2010 (60) | Some concerns | Low Risk | Low Risk | Low Risk | Low Risk | Some concerns |
| van Eijndhoven et al. 2020 (61) | Some concerns | Low Risk | Low Risk | Low Risk | Low Risk | Some concerns |
| Yan et al. 2022 (43) | Some concerns | Some concerns | Low Risk | Low Risk | Low Risk | Some concerns |
| Zheng et al. 2010 (62) | Some concerns | Low Risk | Low Risk | Low Risk | Some concerns | Some concerns |
| Ramasubramanian et al. 2022 (18) | Some concerns | Some concerns | Low Risk | Some concerns | Some concerns | Some concerns |
| Ahmed et al. 2023 (22) | Low Risk | Low Risk | Low Risk | Low Risk | Some concerns | Some concerns |
| Su et al. 2023 (30) | Some concerns | Low Risk | Low Risk | Low Risk | Some concerns | Some concerns |
| Akpinar et al. 2022 (49) | Some concerns | High Risk | High Risk | Low Risk | Some concerns | High Risk |
| Bulteau et al. 2022 (68) | Low Risk | Low Risk | Low Risk | Low Risk | Some concerns | Some concerns |

# 4. Assessment of publication bias

Funnel plots were used to visually assess publication bias. Asymmetry of the funnel plot suggests possible bias, for example showing a lack of small studies with non-significant results. Funnel plots are shown for primary and secondary outcomes.

## **Supplementary Figure S4.1. Funnel plot for the response rate outcome**


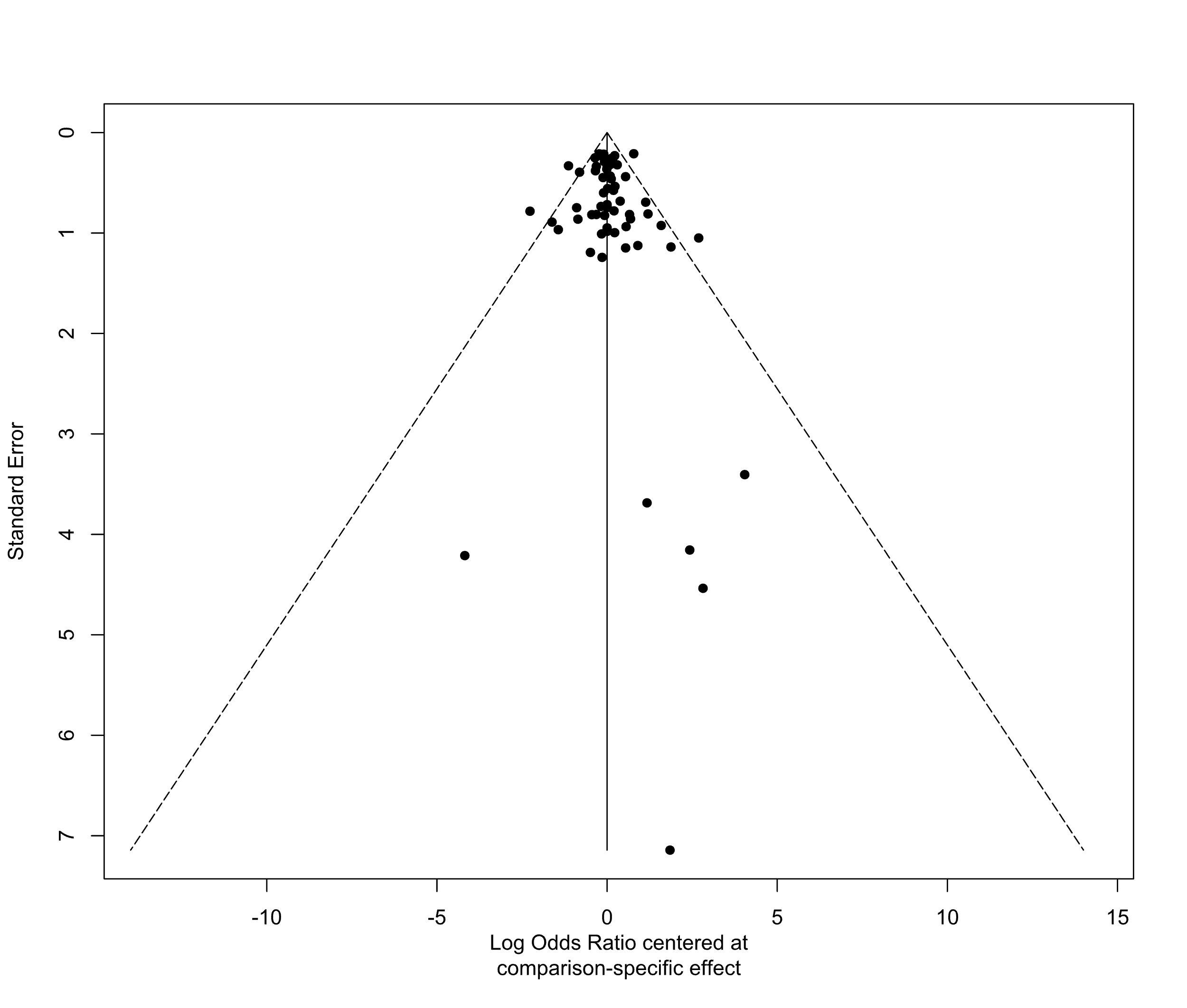


## **Supplementary Figure S4.2. Funnel plot for the standardized mean difference outcome**

**
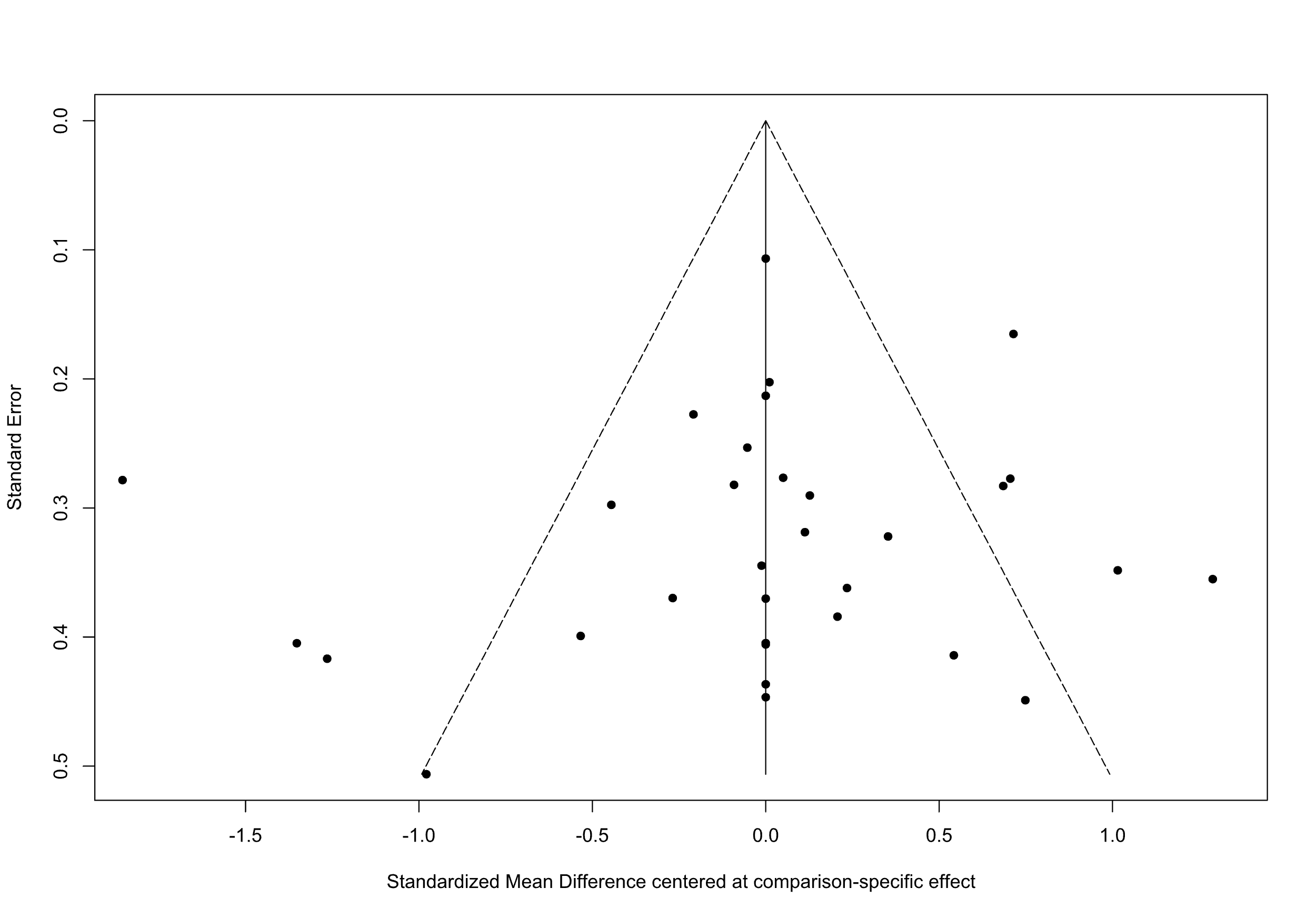
**

## **Supplementary Figure S4.3. Funnel plot for the remission outcome**

**
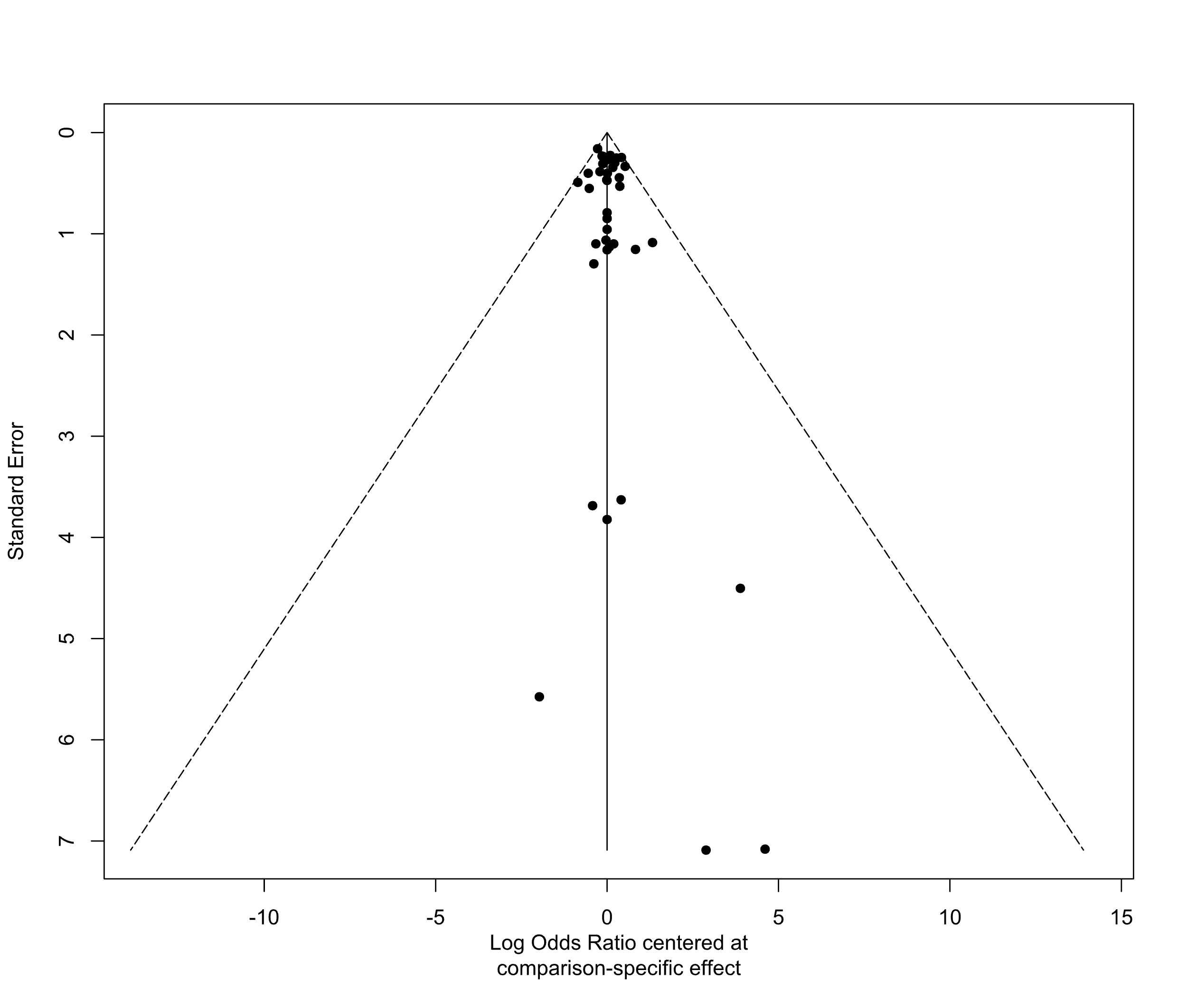
**

## **Supplementary Figure S4.4. Funnel plot for the tolerance outcome**

**
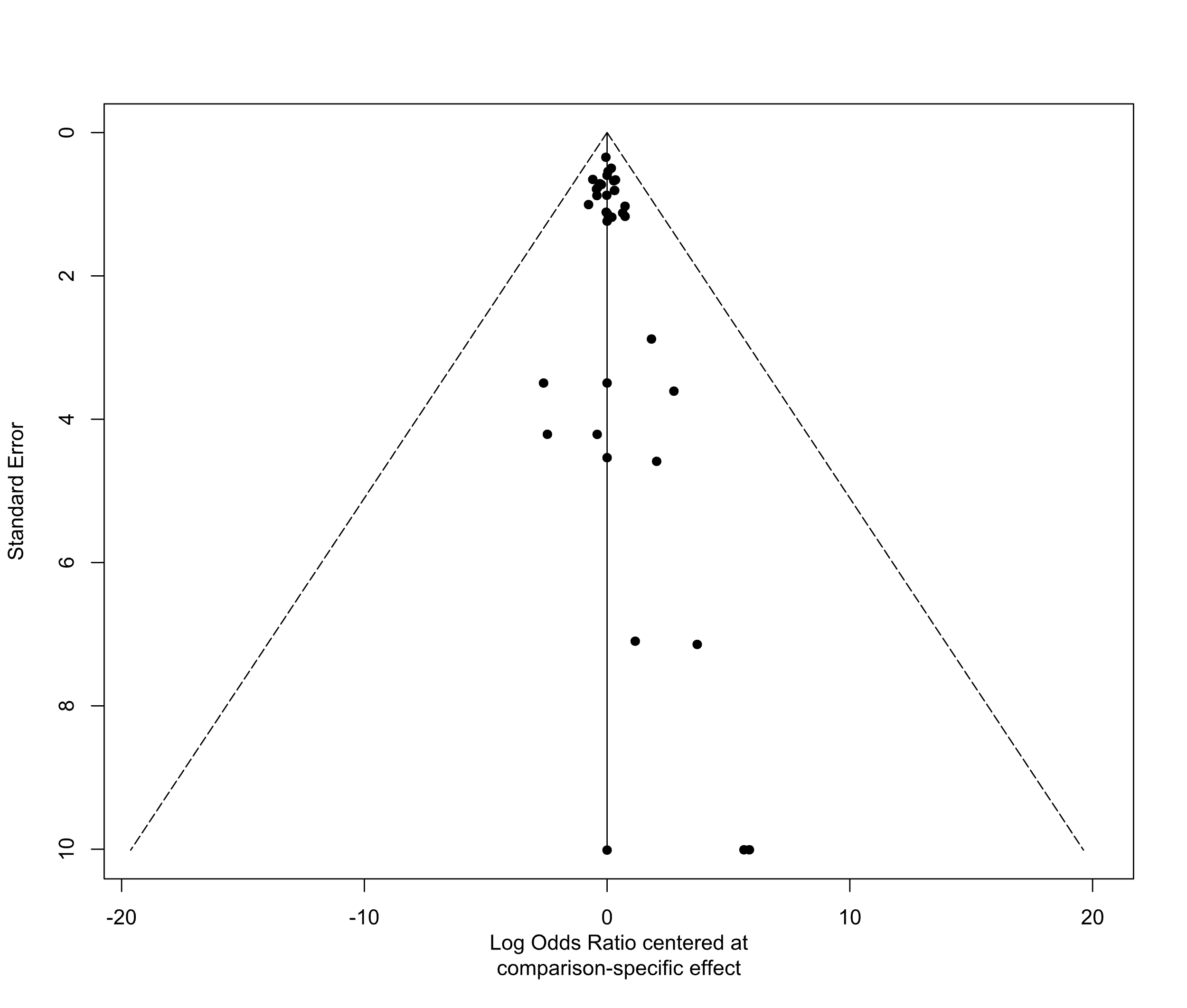
**

# 5. Analysis of efficacy

### **Supplementary Table S5.1. League table for the response rate* outcome**

| **Aripiprazol** |  |  |  |  |  |  |  |  |  |  |  |  |  |  |  |  |  |  |  |  |  |  |  |  |  |
| --- | --- | --- | --- | --- | --- | --- | --- | --- | --- | --- | --- | --- | --- | --- | --- | --- | --- | --- | --- | --- | --- | --- | --- | --- | --- |
| 0.48 (0.09–2.74) | **Ayahuasca** |  |  |  |  |  |  |  |  |  |  |  |  |  |  |  |  |  |  |  |  |  |  |  |  |
| 1.24 (0.65–2.37) | 2.57 (0.45–14.82) | **Brexpiprazole** |  |  |  |  |  |  |  |  |  |  |  |  |  |  |  |  |  |  |  |  |  |  |  |
| 1.80 (0.25–12.71) | 3.71 (0.29–47.37) | 1.44 (0.21–10.18) | **Buspirone** |  |  |  |  |  |  |  |  |  |  |  |  |  |  |  |  |  |  |  |  |  |  |
| 0.30 (0.04–2.38) | 0.61 (0.04–8.62) | 0.24 (0.03–1.94) | 0.17 (0.01–2.70) | **D-Cycloserine** |  |  |  |  |  |  |  |  |  |  |  |  |  |  |  |  |  |  |  |  |  |
| 1.72 (0.49–6.09) | 3.55 (0.45–27.95) | 1.38 (0.38–5.01) | 0.96 (0.10–9.10) | 5.79 (0.55–61.36) | **DBS** |  |  |  |  |  |  |  |  |  |  |  |  |  |  |  |  |  |  |  |  |
| 0.15 (0.04–0.50) | 0.31 (0.04–2.35) | 0.12 (0.03–0.42) | 0.08 (0.01–0.77) | 0.50 (0.05–5.18) | 0.09 (0.02–0.45) | **ECT** |  |  |  |  |  |  |  |  |  |  |  |  |  |  |  |  |  |  |  |
| 2.15 (0.96–4.80) | 4.45 (0.72–27.34) | 1.73 (0.75–4.00) | 1.20 (0.16–9.11) | 7.24 (0.84–62.12) | 1.25 (0.32–4.94) | 14.52 (3.81–55.33) | **Fluoxetine** |  |  |  |  |  |  |  |  |  |  |  |  |  |  |  |  |  |  |
| 0.56 (0.30–1.05) | 1.16 (0.20–6.63) | 0.45 (0.23–0.88) | 0.31 (0.04–2.23) | 1.89 (0.23–15.23) | 0.33 (0.09–1.17) | 3.78 (1.09–13.06) | 0.26 (0.11–0.59) | **Ketamine** |  |  |  |  |  |  |  |  |  |  |  |  |  |  |  |  |  |
| 1.96 (0.75–5.14) | 4.06 (0.61–26.90) | 1.58 (0.59–4.21) | 1.09 (0.15–7.91) | 6.61 (0.72–60.43) | 1.14 (0.26–4.98) | 13.25 (3.14–55.85) | 0.91 (0.30–2.75) | 3.50 (1.31–9.35) | **Lamotrigine** |  |  |  |  |  |  |  |  |  |  |  |  |  |  |  |  |
| 1.25 (0.53–2.96) | 2.58 (0.41–16.32) | 1.01 (0.41–2.46) | 0.70 (0.09–5.42) | 4.21 (0.48–36.92) | 0.73 (0.18–2.97) | 8.44 (2.14–33.34) | 0.58 (0.21–1.60) | 2.23 (0.92–5.40) | 0.64 (0.20–2.00) | **Lanicemine** |  |  |  |  |  |  |  |  |  |  |  |  |  |  |  |
| 2.67 (0.95–7.50) | 5.52 (0.80–37.98) | 2.15 (0.77–5.98) | 1.49 (0.28–7.83) | 8.99 (0.95–84.86) | 1.55 (0.34–7.10) | 18.02 (4.07–79.73) | 1.24 (0.39–3.98) | 4.77 (1.67–13.63) | 1.36 (0.46–3.99) | 2.14 (0.64–7.13) | **Lithium** | . |  |  |  |  |  |  |  |  |  |  |  |  |  |
| 0.29 (0.05–1.58) | 0.61 (0.06–6.32) | 0.24 (0.04–1.30) | 0.16 (0.01–2.02) | 0.99 (0.07–13.44) | 0.17 (0.02–1.29) | 1.98 (0.27–14.59) | 0.14 (0.02–0.80) | 0.52 (0.10–2.86) | 0.15 (0.02–0.95) | 0.23 (0.04–1.42) | 0.11 (0.02–0.72) | **Minocycline** |  |  |  |  |  |  |  |  |  |  |  |  |  |
| 1.46 (0.20–10.53) | 3.02 (0.23–39.07) | 1.17 (0.16–8.43) | 0.81 (0.24–2.71) | 4.91 (0.30–81.37) | 0.85 (0.09–8.21) | 9.85 (1.04–93.22) | 0.68 (0.09–5.26) | 2.61 (0.36–18.98) | 0.74 (0.10–5.49) | 1.17 (0.15–9.27) | 0.55 (0.10–2.95) | 4.98 (0.40–62.43) | **Sodium Valproate** |  |  |  |  |  |  |  |  |  |  |  |  |
| 0.71 (0.08–5.95) | 1.46 (0.10–21.36) | 0.57 (0.07–4.85) | 0.39 (0.02–6.68) | 2.39 (0.13–44.05) | 0.41 (0.04–4.55) | 4.78 (0.44–51.78) | 0.33 (0.04–2.96) | 1.27 (0.15–10.72) | 0.36 (0.04–3.45) | 0.57 (0.06–5.21) | 0.27 (0.03–2.62) | 2.42 (0.17–34.18) | 0.49 (0.03–8.33) | **Nitrous Oxide** |  |  |  |  |  |  |  |  |  |  |  |
| 2.58 (1.24–5.35) | 5.33 (0.89–31.77) | 2.07 (0.96–4.47) | 1.44 (0.19–10.62) | 8.69 (1.04–72.54) | 1.50 (0.40–5.68) | 17.41 (4.77–63.56) | 1.20 (0.61–2.36) | 4.61 (2.17–9.79) | 1.31 (0.46–3.75) | 2.06 (0.79–5.38) | 0.97 (0.32–2.95) | 8.80 (1.55–50.04) | 1.77 (0.23–13.33) | 3.64 (0.42–31.85) | **Olanzapine** |  |  |  |  |  |  |  |  |  |  |
| 1.83 (0.84–3.99) | 3.77 (0.62–22.98) | 1.47 (0.65–3.33) | 1.02 (0.13–7.67) | 6.15 (0.72–52.29) | 1.06 (0.27–4.14) | 12.33 (3.28–46.35) | 0.85 (0.47–1.52) | 3.26 (1.46–7.29) | 0.93 (0.31–2.76) | 1.46 (0.54–3.97) | 0.68 (0.22–2.16) | 6.23 (1.07–36.22) | 1.25 (0.16–9.62) | 2.58 (0.29–22.96) | 0.71 (0.37–1.36) | **Olanzapine/Fluoxetine** |  |  |  |  |  |  |  |  |  |
| 1.90 (1.25–2.91) | 3.94 (0.73–21.20) | 1.53 (0.94–2.49) | 1.06 (0.16–7.17) | 6.42 (0.84–49.22) | 1.11 (0.34–3.65) | 12.86 (4.07–40.63) | 0.89 (0.45–1.75) | 3.40 (2.14–5.41) | 0.97 (0.41–2.31) | 1.52 (0.72–3.23) | 0.71 (0.28–1.83) | 6.50 (1.27–33.29) | 1.31 (0.19–9.00) | 2.69 (0.33–21.65) | 0.74 (0.41–1.34) | 1.04 (0.54–2.01) | **Placebo** |  |  |  |  |  |  |  |  |
| 1.06 (0.33–3.35) | 2.19 (0.30–16.11) | 0.85 (0.26–2.77) | 0.59 (0.07–5.27) | 3.57 (0.36–35.66) | 0.62 (0.12–3.06) | 7.15 (1.48–34.45) | 0.49 (0.14–1.76) | 1.89 (0.59–6.08) | 0.54 (0.14–2.14) | 0.85 (0.23–3.14) | 0.40 (0.10–1.65) | 3.61 (0.51–25.49) | 0.73 (0.08–6.61) | 1.50 (0.14–15.60) | 0.41 (0.12–1.40) | 0.58 (0.17–2.04) | 0.56 (0.19–1.62) | **Psilocybine** |  |  |  |  |  |  |  |
| 1.92 (0.82–4.49) | 3.96 (0.63–24.90) | 1.54 (0.68–3.49) | 1.07 (0.17–6.88) | 6.46 (0.74–56.38) | 1.12 (0.27–4.53) | 12.95 (3.30–50.76) | 0.89 (0.33–2.44) | 3.42 (1.43–8.19) | 0.98 (0.34–2.78) | 1.53 (0.54–4.40) | 0.72 (0.31–1.66) | 6.54 (1.09–39.27) | 1.31 (0.20–8.64) | 2.71 (0.30–24.73) | 0.74 (0.29–1.92) | 1.05 (0.39–2.82) | 1.01 (0.48–2.10) | 1.81 (0.49–6.65) | **Quetiapine** |  |  |  |  |  |  |
| 1.69 (0.51–5.63) | 3.50 (0.46–26.51) | 1.36 (0.42–4.46) | 0.94 (0.14–6.40) | 5.71 (0.56–58.46) | 0.99 (0.19–5.07) | 11.44 (2.29–57.13) | 0.79 (0.21–2.94) | 3.03 (0.90–10.21) | 0.86 (0.24–3.15) | 1.36 (0.35–5.24) | 0.63 (0.25–1.64) | 5.78 (0.80–41.98) | 1.16 (0.17–8.04) | 2.39 (0.22–25.57) | 0.66 (0.18–2.34) | 0.93 (0.25–3.41) | 0.89 (0.29–2.74) | 1.60 (0.34–7.56) | 0.88 (0.34–2.28) | **Quetiapine Mono** |  |  |  |  |  |
| 2.67 (0.38–18.90) | 5.51 (0.43–70.42) | 2.15 (0.30–15.13) | 1.49 (0.46–4.80) | 8.99 (0.55-146.86) | 1.55 (0.16–14.76) | 18.02 (1.94-167.70) | 1.24 (0.16–9.45) | 4.77 (0.67–34.08) | 1.36 (0.19–9.85) | 2.14 (0.27–16.66) | 1.00 (0.19–5.27) | 9.10 (0.74-112.51) | 1.83 (0.55–6.10) | 3.77 (0.22–63.78) | 1.03 (0.14–7.66) | 1.46 (0.19–11.03) | 1.40 (0.21–9.47) | 2.52 (0.28–22.54) | 1.39 (0.22–8.97) | 1.58 (0.23–10.70) | **Risperidone** |  |  |  |  |
| 0.47 (0.24–0.93) | 0.98 (0.17–5.73) | 0.38 (0.19–0.78) | 0.26 (0.04–1.92) | 1.60 (0.19–13.13) | 0.28 (0.07–1.02) | 3.21 (1.15–8.92) | 0.22 (0.09–0.52) | 0.85 (0.42–1.71) | 0.24 (0.09–0.67) | 0.38 (0.15–0.95) | 0.18 (0.06–0.52) | 1.62 (0.29–9.02) | 0.33 (0.04–2.41) | 0.67 (0.08–5.77) | 0.18 (0.08–0.41) | 0.26 (0.11–0.60) | 0.25 (0.15–0.42) | 0.45 (0.14–1.48) | 0.25 (0.10–0.61) | 0.28 (0.08–0.97) | 0.18 (0.02–1.29) | **rTMS** |  |  |  |
| 0.40 (0.16–0.96) | 0.82 (0.13–5.23) | 0.32 (0.13–0.80) | 0.22 (0.03–1.74) | 1.34 (0.15–11.83) | 0.23 (0.06–0.96) | 2.68 (0.79–9.12) | 0.18 (0.07–0.52) | 0.71 (0.29–1.75) | 0.20 (0.06–0.65) | 0.32 (0.11–0.93) | 0.15 (0.04–0.50) | 1.36 (0.22–8.26) | 0.27 (0.03–2.18) | 0.56 (0.06–5.19) | 0.15 (0.06–0.41) | 0.22 (0.08–0.60) | 0.21 (0.10–0.45) | 0.38 (0.10–1.41) | 0.21 (0.07–0.60) | 0.23 (0.06–0.92) | 0.15 (0.02–1.17) | 0.84 (0.43–1.64) | **TBS** |  |  |
| 0.61 (0.05–6.92) | 1.26 (0.07–23.49) | 0.49 (0.04–5.63) | 0.34 (0.02–7.26) | 2.06 (0.09–47.57) | 0.36 (0.02–5.14) | 4.12 (0.29–57.68) | 0.28 (0.02–3.41) | 1.09 (0.10–12.46) | 0.31 (0.02–3.96) | 0.49 (0.04–5.99) | 0.23 (0.02–2.99) | 2.08 (0.12–37.69) | 0.42 (0.02–9.04) | 0.86 (0.04–20.58) | 0.24 (0.02–2.78) | 0.33 (0.03–3.99) | 0.32 (0.03–3.50) | 0.58 (0.04–7.92) | 0.32 (0.03–3.89) | 0.36 (0.03–5.06) | 0.23 (0.01–4.89) | 1.29 (0.11–14.83) | 1.54 (0.12–18.91) | **tDCS** |  |
| 1.67 (0.35–8.02) | 3.45 (0.36–33.15) | 1.34 (0.28–6.42) | 0.93 (0.29–2.98) | 5.62 (0.44–71.07) | 0.97 (0.14–6.66) | 11.26 (1.68–75.34) | 0.78 (0.15–4.08) | 2.98 (0.61–14.50) | 0.85 (0.17–4.21) | 1.33 (0.25–7.23) | 0.62 (0.19–2.04) | 5.69 (0.61–52.74) | 1.14 (0.34–3.79) | 2.35 (0.18–30.98) | 0.65 (0.13–3.29) | 0.91 (0.18–4.75) | 0.88 (0.19–3.98) | 1.57 (0.25–10.06) | 0.87 (0.20–3.72) | 0.98 (0.22–4.50) | 0.62 (0.19–2.01) | 3.51 (0.71–17.45) | 4.20 (0.77–22.96) | 2.73 (0.16–46.23) | **Thyroid Hormone** |

*Response rate was defined as a ≥50% reduction in depressive symptoms measured by any standardized depression rating scale. Abbreviations: DBS: deep brain stimulation; ECT: electroconvulsive therapy; rTMS: repetitive transcranial magnetic therapy; TBS: theta burst stimulation; tDCS: transcranial direct current stimulation

### **Supplementary Figure S5.1. Ranking of included treatments for the response rate* outcome**

Network meta-analyses incorporate both direct evidence (eg, trials that directly compare two treatments) and indirect evidence (eg, studies that indirectly compare treatments by using a common comparator) to estimate the relative effectivenesss of each included treatment. This further allows included treatments to be ranked for each outcome.


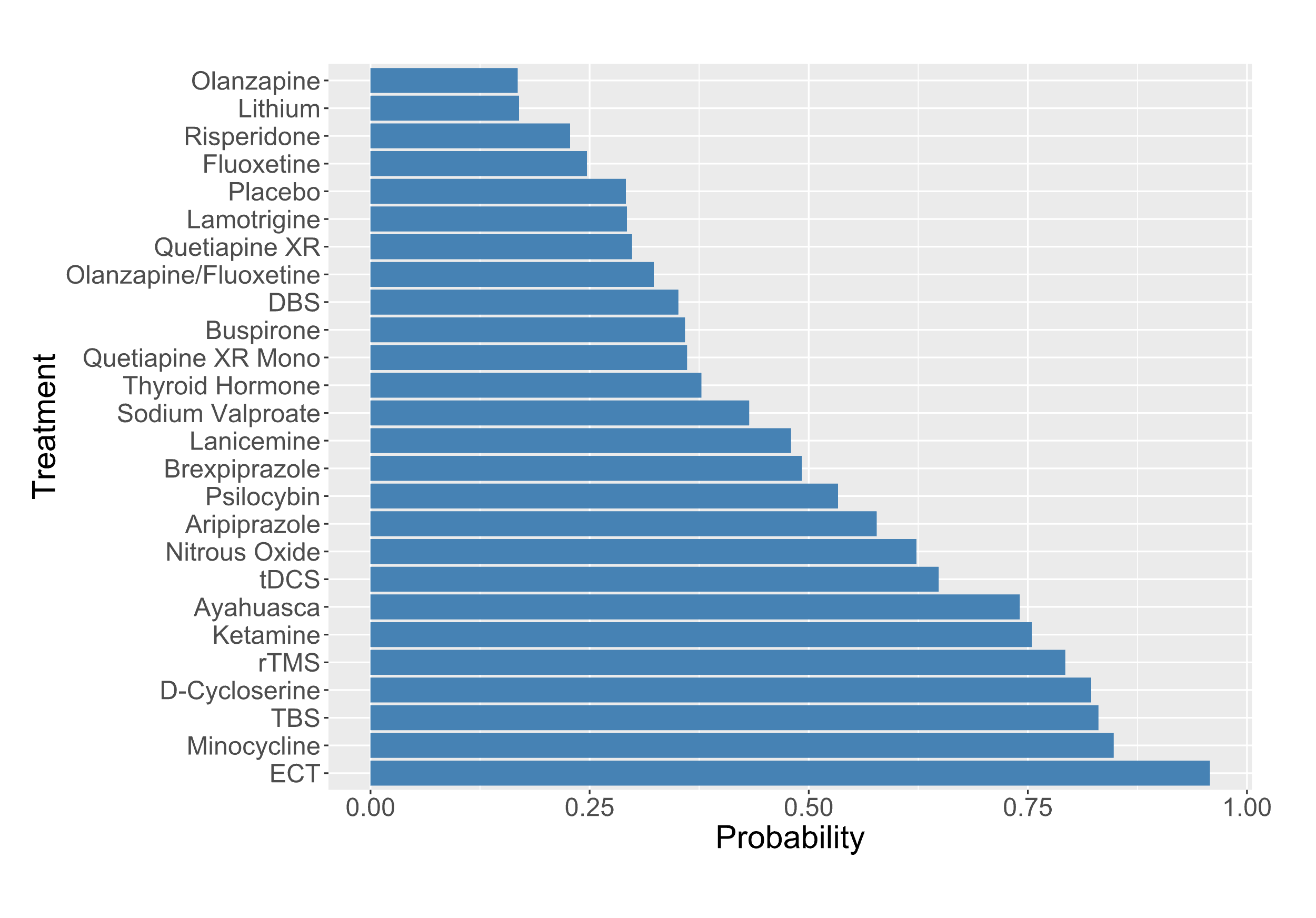


*Response rate was defined as a ≥50% reduction in depressive symptoms measured by any standardized depression rating scale. Abbreviations: DBS: deep brain stimulation; ECT: electroconvulsive therapy; rTMS: repetitive transcranial magnetic therapy; TBS: theta burst stimulation; tDCS: transcranial direct current stimulation; XR: extended release

### **Supplementary Figure S5.2. Network graph for the standardized mean differences outcome**

**
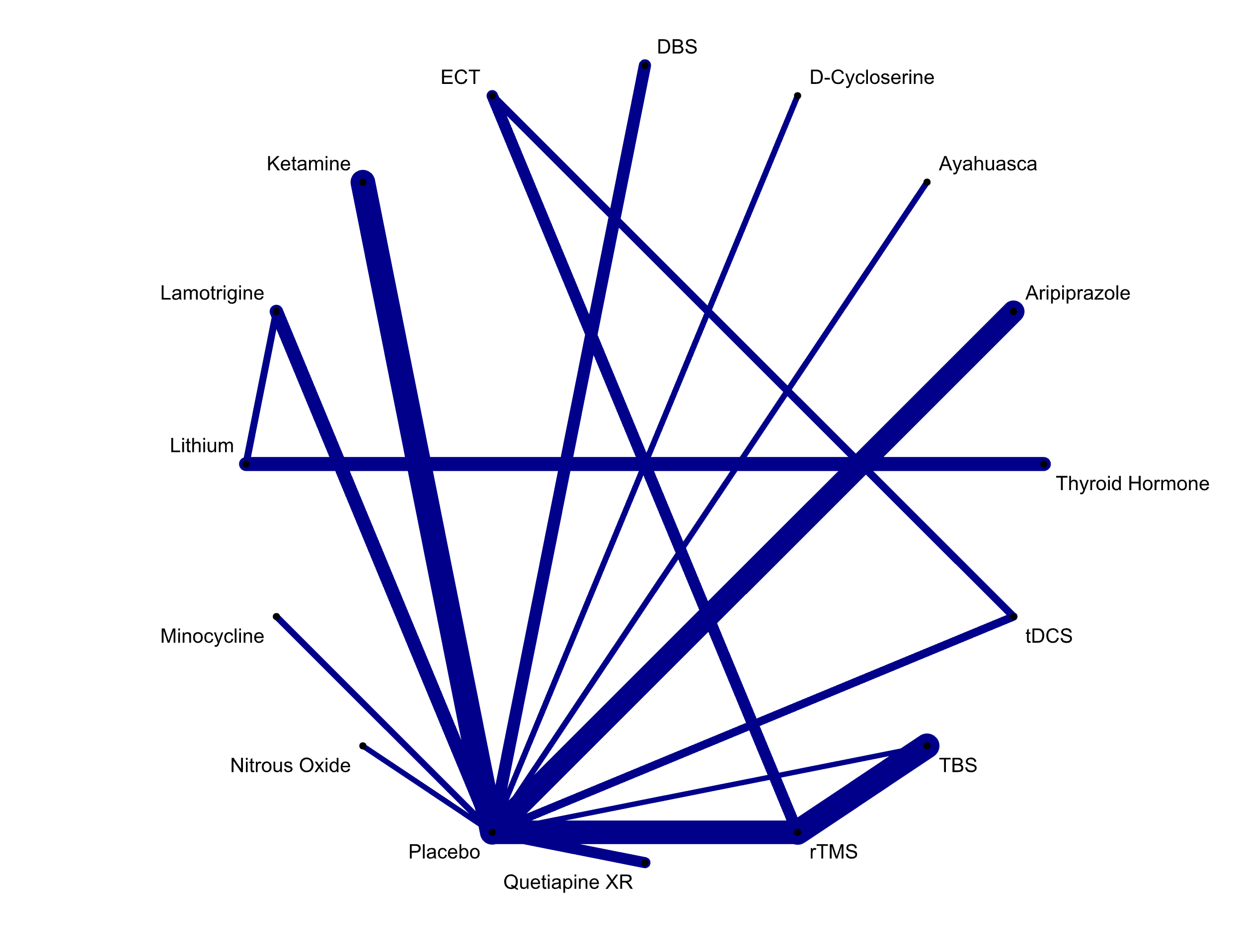
**

Abbreviations: DBS: deep brain stimulation; ECT: electroconvulsive therapy; rTMS: repetitive transcranial magnetic therapy; TBS: theta burst stimulation; tDCS: transcranial direct current stimulation; XR: extended release

### **Supplementary Figure S5.3. Forest plot for the standardized mean differences outcome**

**
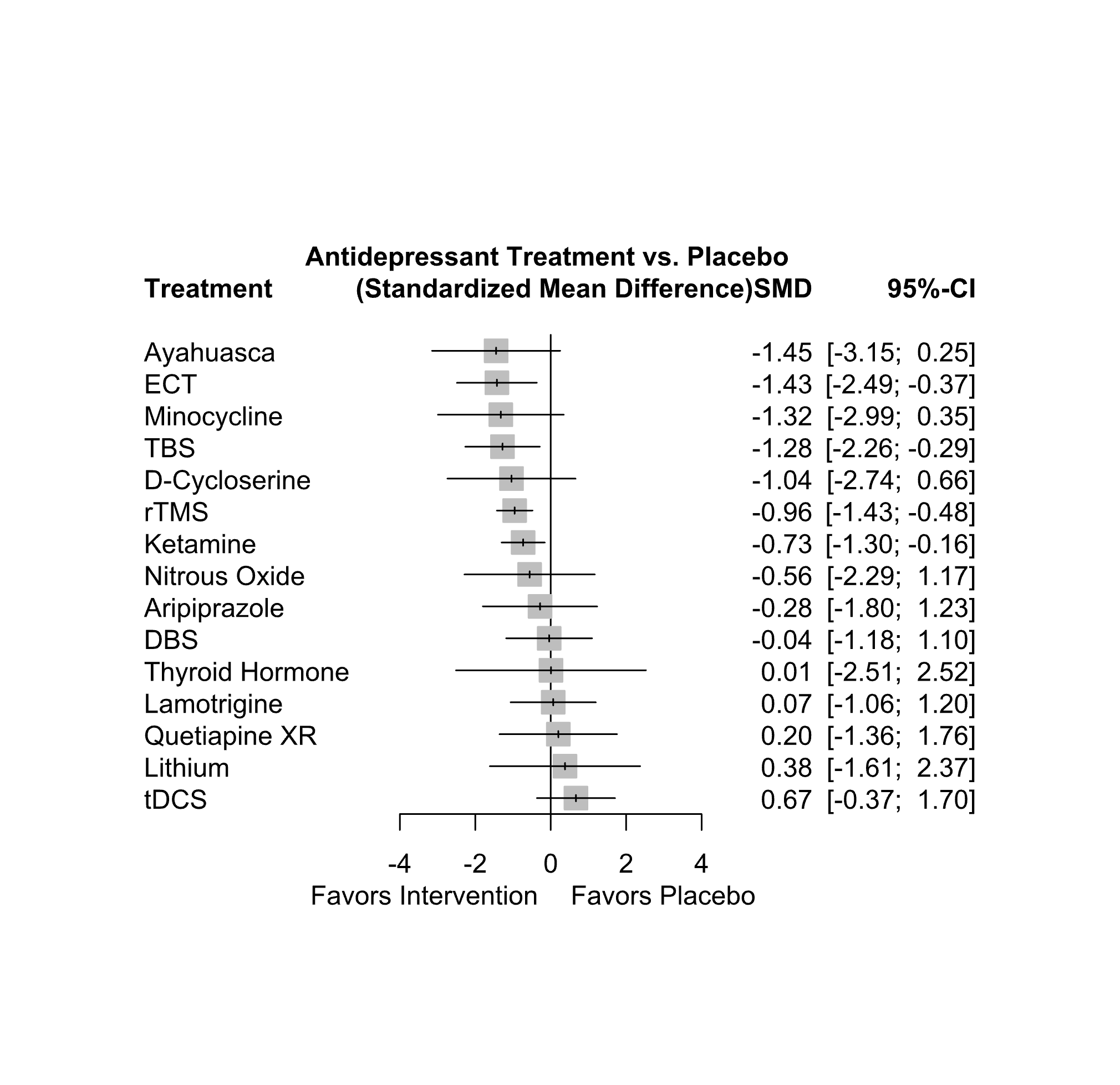
**

Abbreviations: CI: confidence interval; DBS: deep brain stimulation; ECT: electroconvulsive therapy; OR: odds ratio; rTMS: repetitive transcranial magnetic therapy; TBS: theta burst stimulation; tDCS: transcranial direct current stimulation; XR: extended release

### **Supplementary Table S5.2. League table for the standardized mean difference outcome**

| **Aripiprazole** |  |  |  |  |  |  |  |  |  |  |  |  |  |  |  |
| --- | --- | --- | --- | --- | --- | --- | --- | --- | --- | --- | --- | --- | --- | --- | --- |
| 1.17 (-1.11–3.44) | **Ayahuasca** |  |  |  |  |  |  |  |  |  |  |  |  |  |  |
| 0.76 (-1.52–3.03) | -0.41 (-2.81–1.99) | **D-Cycloserine** |  |  |  |  |  |  |  |  |  |  |  |  |  |
| -0.24 (-2.14–1.66) | -1.41 (-3.45–0.64) | -1.00 (-3.04–1.05) | **DBS** |  |  |  |  |  |  |  |  |  |  |  |  |
| 1.14 (-0.71–2.99) | -0.02 (-2.02–1.98) | 0.39 (-1.61–2.39) | 1.39 (-0.17–2.94) | **ECT** |  |  |  |  |  |  |  |  |  |  |  |
| 0.45 (-1.17–2.07) | -0.72 (-2.51–1.08) | -0.31 (-2.10–1.48) | 0.69 (-0.59–1.96) | -0.70 (-1.90–0.51) | **Ketamine** |  |  |  |  |  |  |  |  |  |  |
| -0.35 (-2.24–1.54) | -1.51 (-3.55–0.53) | -1.11 (-3.15–0.94) | -0.11 (-1.71–1.50) | -1.49 (-3.04–0.06) | -0.80 (-2.06–0.47) | **Lamotrigine** |  |  |  |  |  |  |  |  |  |
| -0.66 (-3.16–1.84) | -1.83 (-4.44–0.79) | -1.42 (-4.04–1.20) | -0.42 (-2.72–1.88) | -1.80 (-4.06–0.45) | -1.11 (-3.18–0.96) | -0.31 (-1.95–1.33) | **Lithium** |  |  |  |  |  |  |  |  |
| 1.04 (-1.22–3.29) | -0.13 (-2.51–2.26) | 0.28 (-2.10–2.66) | 1.28 (-0.74–3.30) | -0.10 (-2.08–1.87) | 0.59 (-1.17–2.36) | 1.39 (-0.63–3.40) | 1.70 (-0.90–4.30) | **Minocycline** |  |  |  |  |  |  |  |
| 0.28 (-2.02–2.58) | -0.89 (-3.31–1.54) | -0.48 (-2.90–1.94) | 0.52 (-1.55–2.59) | -0.87 (-2.90–1.16) | -0.17 (-1.99–1.65) | 0.62 (-1.44–2.69) | 0.94 (-1.70–3.57) | -0.76 (-3.17–1.64) | **Nitrous Oxide** |  |  |  |  |  |  |
| -0.28 (-1.80–1.23) | -1.45 (-3.15–0.25) | -1.04 (-2.74–0.66) | -0.04 (-1.18–1.10) | -1.43 (-2.49--0.37) | -0.73 (-1.30--0.16) | 0.07 (-1.06–1.20) | 0.38 (-1.61–2.37) | -1.32 (-2.99–0.35) | -0.56 (-2.29–1.17) | **Placebo** |  |  |  |  |  |
| -0.48 (-2.66–1.69) | -1.65 (-3.96–0.66) | -1.24 (-3.55–1.06) | -0.24 (-2.17–1.69) | -1.63 (-3.51–0.26) | -0.93 (-2.59–0.73) | -0.14 (-2.06–1.79) | 0.18 (-2.35–2.71) | -1.52 (-3.81–0.76) | -0.76 (-3.09–1.57) | -0.20 (-1.76–1.36) | **Quetiapine XR** |  |  |  |  |
| 0.67 (-0.92–2.26) | -0.49 (-2.26–1.27) | -0.08 (-1.85–1.68) | 0.91 (-0.32–2.15) | -0.47 (-1.47–0.53) | 0.23 (-0.52–0.97) | 1.02 (-0.20–2.25) | 1.33 (-0.71–3.38) | -0.37 (-2.10–1.37) | 0.40 (-1.39–2.19) | 0.96 ( 0.48–1.43) | 1.16 (-0.47–2.79) | **rTMS** |  |  |  |
| 0.99 (-0.81–2.80) | -0.17 (-2.14–1.79) | 0.24 (-1.73–2.20) | 1.24 (-0.27–2.74) | -0.15 (-1.51–1.21) | 0.55 (-0.59–1.69) | 1.34 (-0.16–2.84) | 1.66 (-0.57–3.88) | -0.04 (-1.98–1.89) | 0.72 (-1.27–2.71) | 1.28 ( 0.29–2.26) | 1.48 (-0.37–3.32) | 0.32 (-0.62–1.26) | **TBS** |  |  |
| -0.95 (-2.79–0.88) | -2.12 (-4.11--0.13) | -1.71 (-3.70–0.28) | -0.71 (-2.25–0.83) | -2.10 (-3.28--0.91) | -1.40 (-2.58--0.22) | -0.60 (-2.14–0.93) | -0.29 (-2.54–1.95) | -1.99 (-3.96--0.03) | -1.23 (-3.24–0.79) | -0.67 (-1.70–0.37) | -0.47 (-2.34–1.40) | -1.63 (-2.71--0.54) | -1.95 (-3.35--0.55) | **tDCS** |  |
| -0.29 (-3.23–2.65) | -1.46 (-4.49–1.58) | -1.05 (-4.08–1.99) | -0.05 (-2.81–2.71) | -1.43 (-4.16–1.30) | -0.74 (-3.32–1.84) | 0.06 (-2.19–2.31) | 0.37 (-1.17–1.91) | -1.33 (-4.35–1.69) | -0.57 (-3.62–2.49) | -0.01 (-2.52–2.51) | 0.19 (-2.77–3.16) | -0.96 (-3.52–1.60) | -1.28 (-3.99–1.42) | 0.66 (-2.06–3.38) | **Thyroid Hormone** |

Abbreviations: DBS: deep brain stimulation; ECT: electroconvulsive therapy; rTMS: repetitive transcranial magnetic therapy; TBS: theta burst stimulation; tDCS: transcranial direct current stimulation

### **Supplementary Figure S5.4. Ranking of included treatments for standardized mean differences outcome**

**
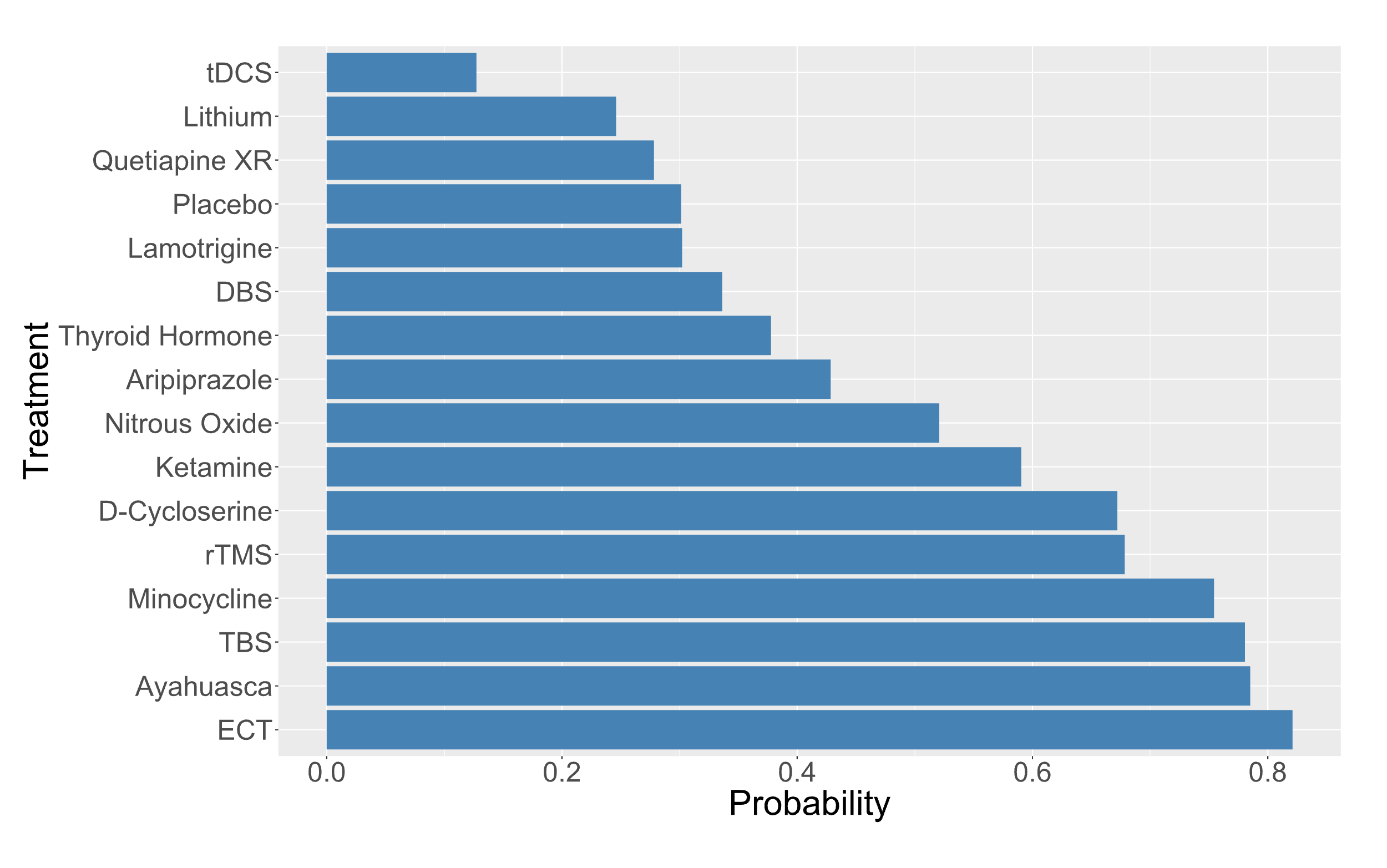
**

Abbreviations: DBS: deep brain stimulation; ECT: electroconvulsive therapy; rTMS: repetitive transcranial magnetic therapy; TBS: theta burst stimulation; tDCS: transcranial direct current stimulation; XR: extended release

### **Supplementary Figure S5.5. Network graph for the remission* outcome**

**
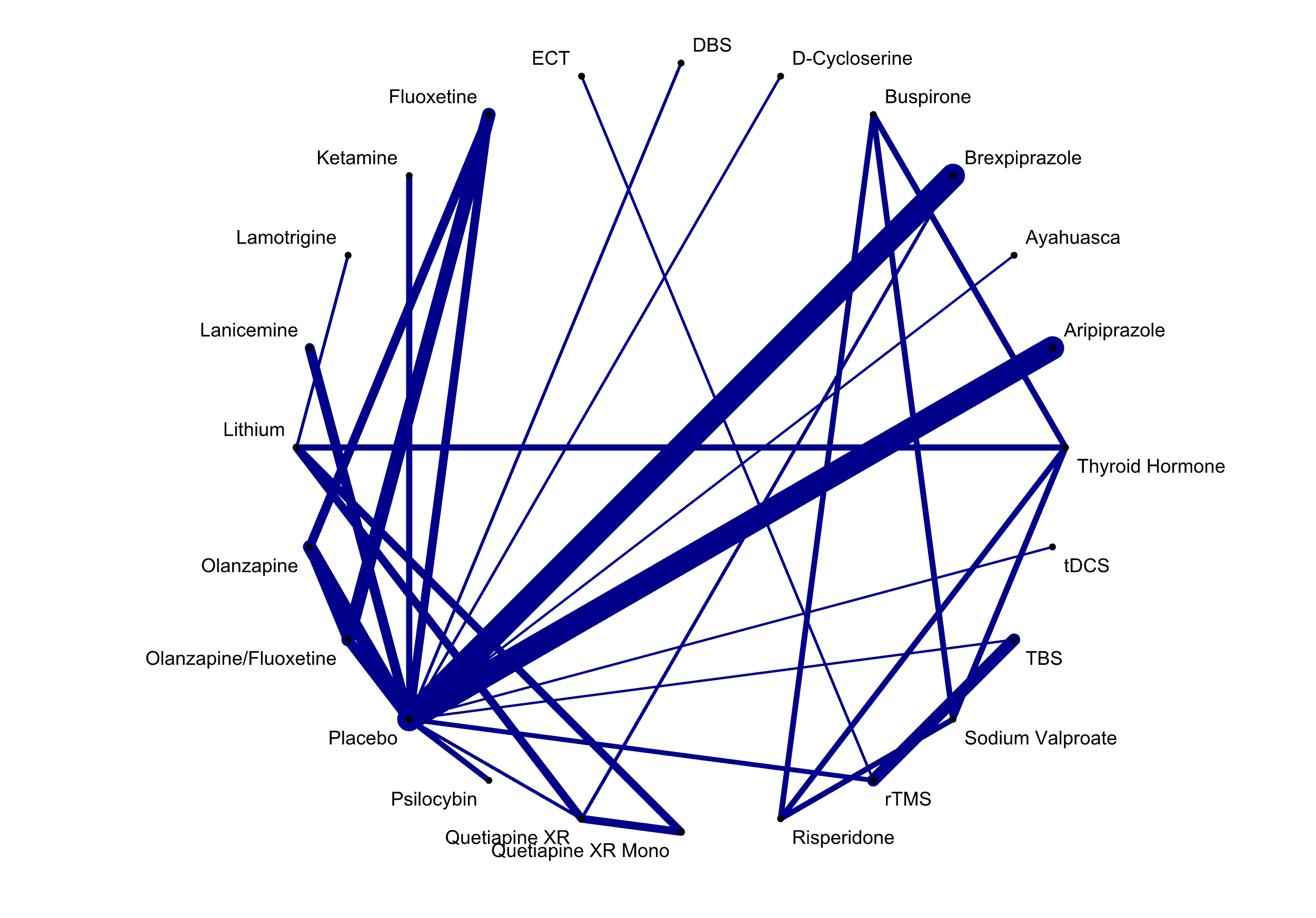
**

*Remission was defined as a Montgomery-Asberg Depression Rating Scale (MADRS) score of ≤ 10 or a Hamilton Depression Rating Scale (HAMD) score of ≤ 7. Abbreviations: DBS: deep brain stimulation; ECT: electroconvulsive therapy; rTMS: repetitive transcranial magnetic therapy; TBS: theta burst stimulation; tDCS: transcranial direct current stimulation; XR: extended release

### **Supplementary Figure S5.6. Forest plot for the remission* outcome**


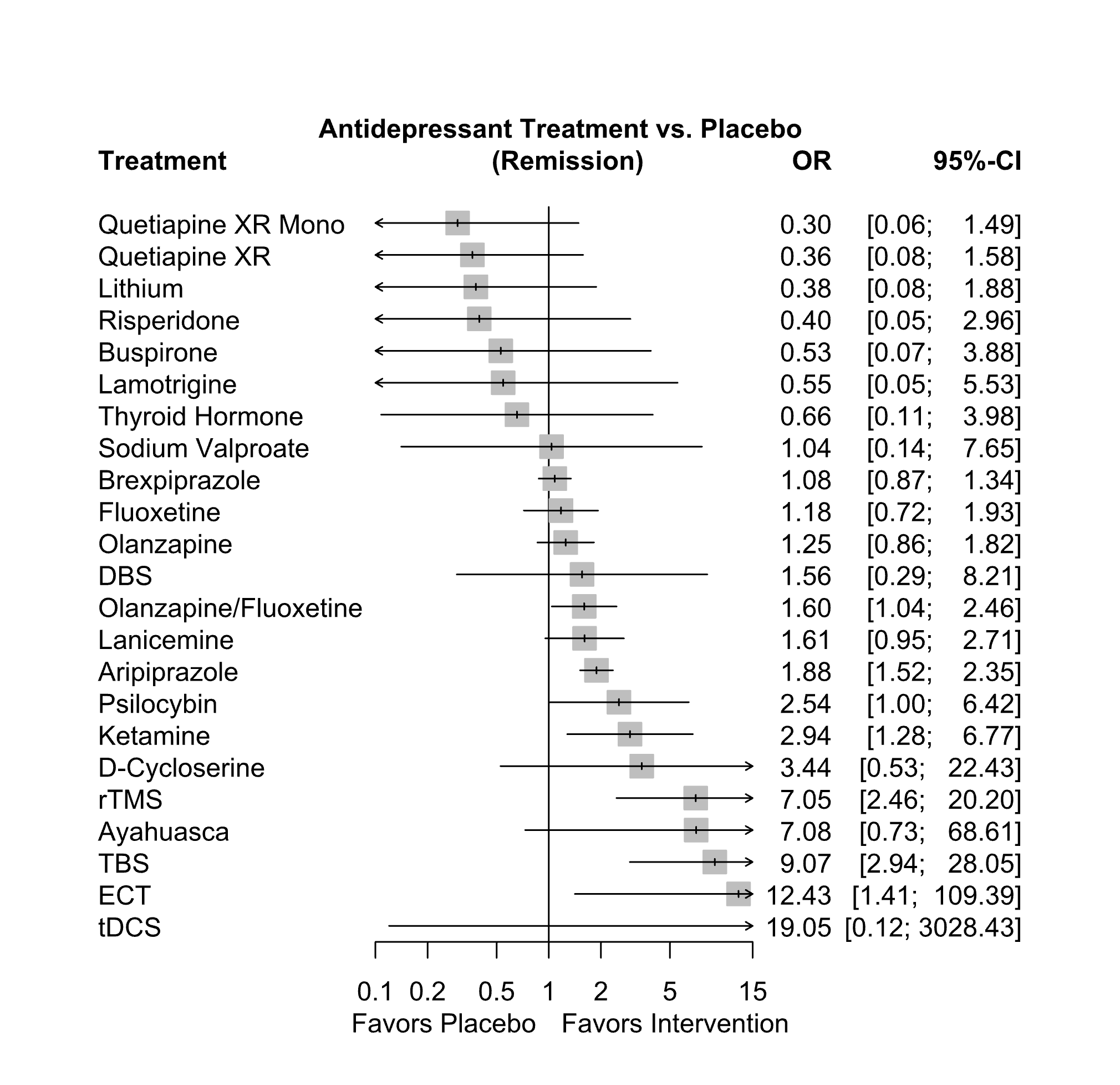


*Remission was defined as a Montgomery-Asberg Depression Rating Scale (MADRS) score of ≤ 10 or a Hamilton Depression Rating Scale (HAMD) score of ≤ 7. Abbreviations: CI: confidence interval; DBS: deep brain stimulation; ECT: electroconvulsive therapy; OR: odds ratio; rTMS: repetitive transcranial magnetic therapy; TBS: theta burst stimulation; tDCS: transcranial direct current stimulation; XR: extended release

### **Supplementary Table S5.3. League table for the remission* outcome**

| **Aripiprazole** |  |  |  |  |  |  |  |  |  |  |  |  |  |  |  |  |  |  |  |  |  |  |  |
| --- | --- | --- | --- | --- | --- | --- | --- | --- | --- | --- | --- | --- | --- | --- | --- | --- | --- | --- | --- | --- | --- | --- | --- |
| 0.27 (0.03–2.60) | **Ayahuasca** |  |  |  |  |  |  |  |  |  |  |  |  |  |  |  |  |  |  |  |  |  |  |
| 1.74 (1.28–2.36) | 6.54 (0.67–63.97) | **Brexpiprazole** |  |  |  |  |  |  |  |  |  |  |  |  |  |  |  |  |  |  |  |  |  |
| 3.57 (0.48–26.51) | 13.40 (0.65–275.17) | 2.05 (0.28–15.03) | **Buspirone** |  |  |  |  |  |  |  |  |  |  |  |  |  |  |  |  |  |  |  |  |
| 0.55 (0.08–3.62) | 2.06 (0.11–39.18) | 0.32 (0.05–2.08) | 0.15 (0.01–2.38) | **D-Cycloserine** |  |  |  |  |  |  |  |  |  |  |  |  |  |  |  |  |  |  |  |
| 1.21 (0.23–6.49) | 4.55 (0.27–76.03) | 0.70 (0.13–3.73) | 0.34 (0.03–4.56) | 2.21 (0.18–27.12) | **DBS** |  |  |  |  |  |  |  |  |  |  |  |  |  |  |  |  |  |  |
| 0.15 (0.02–1.35) | 0.57 (0.02–13.21) | 0.09 (0.01–0.77) | 0.04 (0.00–0.81) | 0.28 (0.02–4.88) | 0.13 (0.01–1.93) | **ECT** |  |  |  |  |  |  |  |  |  |  |  |  |  |  |  |  |  |
| 1.60 (0.93–2.75) | 6.02 (0.59–61.49) | 0.92 (0.54–1.58) | 0.45 (0.06–3.50) | 2.92 (0.42–20.32) | 1.32 (0.23–7.50) | 10.57 (1.14–98.27) | **Fluoxetine** |  |  |  |  |  |  |  |  |  |  |  |  |  |  |  |  |
| 0.64 (0.27–1.52) | 2.41 (0.21–27.05) | 0.37 (0.16–0.87) | 0.18 (0.02–1.56) | 1.17 (0.15–9.10) | 0.53 (0.08–3.40) | 4.23 (0.41–43.39) | 0.40 (0.15–1.05) | **Ketamine** |  |  |  |  |  |  |  |  |  |  |  |  |  |  |  |
| 3.46 (0.34–35.42) | 12.99 (0.51–332.95) | 1.99 (0.20–20.11) | 0.97 (0.12–7.58) | 6.30 (0.32–124.21) | 2.85 (0.16–49.43) | 22.80 (0.95–546.81) | 2.16 (0.20–23.04) | 5.40 (0.46–63.29) | **Lamotrigine** |  |  |  |  |  |  |  |  |  |  |  |  |  |  |
| 1.17 (0.66–2.06) | 4.40 (0.43–45.24) | 0.67 (0.38–1.18) | 0.33 (0.04–2.58) | 2.14 (0.30–14.97) | 0.97 (0.17–5.53) | 7.73 (0.83–72.32) | 0.73 (0.36–1.50) | 1.83 (0.68–4.89) | 0.34 (0.03–3.64) | **Lanicemine** |  |  |  |  |  |  |  |  |  |  |  |  |  |
| 4.96 (0.99–24.92) | 18.65 (1.16–299.75) | 2.85 (0.58–14.08) | 1.39 (0.42–4.58) | 9.05 (0.77–106.44) | 4.10 (0.41–41.17) | 32.74 (2.20–486.67) | 3.10 (0.58–16.50) | 7.75 (1.28–47.02) | 1.44 (0.27–7.68) | 4.24 (0.79–22.78) | **Lithium** |  |  |  |  |  |  |  |  |  |  |  |  |
| 1.51 (0.97–2.33) | 5.66 (0.57–56.50) | 0.87 (0.56–1.33) | 0.42 (0.06–3.21) | 2.74 (0.41–18.59) | 1.24 (0.23–6.84) | 9.93 (1.09–90.21) | 0.94 (0.58–1.52) | 2.35 (0.94–5.86) | 0.44 (0.04–4.55) | 1.28 (0.67–2.45) | 0.30 (0.06–1.57) | **Olanzapine** |  |  |  |  |  |  |  |  |  |  |  |
| 1.18 (0.73–1.91) | 4.42 (0.44–44.61) | 0.68 (0.42–1.09) | 0.33 (0.04–2.54) | 2.15 (0.31–14.71) | 0.97 (0.17–5.42) | 7.76 (0.85–71.26) | 0.73 (0.50–1.07) | 1.84 (0.72–4.70) | 0.34 (0.03–3.59) | 1.00 (0.51–1.98) | 0.24 (0.05–1.24) | 0.78 (0.51–1.20) | **Olanzapine/Fluoxetine** |  |  |  |  |  |  |  |  |  |  |
| 1.88 (1.52–2.35) | 7.08 (0.73–68.61) | 1.08 (0.87–1.34) | 0.53 (0.07–3.88) | 3.44 (0.53–22.43) | 1.56 (0.29–8.21) | 12.43 (1.41–109.39) | 1.18 (0.72–1.93) | 2.94 (1.28–6.77) | 0.55 (0.05–5.53) | 1.61 (0.95–2.71) | 0.38 (0.08–1.88) | 1.25 (0.86–1.82) | 1.60 (1.04–2.46) | **Placebo** |  |  |  |  |  |  |  |  |  |
| 0.74 (0.29–1.92) | 2.79 (0.24–32.40) | 0.43 (0.16–1.10) | 0.21 (0.02–1.88) | 1.35 (0.17–10.97) | 0.61 (0.09–4.12) | 4.89 (0.46–52.05) | 0.46 (0.16–1.32) | 1.16 (0.33–4.03) | 0.21 (0.02–2.60) | 0.63 (0.22–1.84) | 0.15 (0.02–0.95) | 0.49 (0.18–1.34) | 0.63 (0.23–1.75) | 0.39 (0.16–1.00) | **Psilocybin** |  |  |  |  |  |  |  |  |
| 5.20 (1.18–22.97) | 19.53 (1.31–292.01) | 2.99 (0.69–12.96) | 1.46 (0.38–5.61) | 9.48 (0.87–102.71) | 4.29 (0.47–39.50) | 34.28 (2.48–473.07) | 3.24 (0.69–15.29) | 8.11 (1.50–43.96) | 1.50 (0.25–9.01) | 4.44 (0.93–21.12) | 1.05 (0.56–1.96) | 3.45 (0.76–15.74) | 4.41 (0.95–20.42) | 2.76 (0.63–11.99) | 7.00 (1.23–39.83) | **Quetiapine XR** |  |  |  |  |  |  |  |
| 6.33 (1.25–32.02) | 23.77 (1.47–383.85) | 3.64 (0.73–18.09) | 1.77 (0.46–6.90) | 11.54 (0.98–136.38) | 5.22 (0.52–52.77) | 41.73 (2.79–623.29) | 3.95 (0.73–21.20) | 9.87 (1.61–60.37) | 1.83 (0.30–11.06) | 5.40 (1.00–29.27) | 1.27 (0.66–2.44) | 4.20 (0.81–21.90) | 5.37 (1.02–28.37) | 3.36 (0.67–16.74) | 8.52 (1.33–54.52) | 1.22 (0.64–2.33) | **Quetiapine XR Mono** |  |  |  |  |  |  |
| 4.75 (0.63–35.78) | 17.83 (0.86–369.59) | 2.73 (0.37–20.28) | 1.33 (0.54–3.28) | 8.65 (0.55–135.11) | 3.92 (0.29–53.16) | 31.30 (1.62–604.09) | 2.96 (0.37–23.42) | 7.41 (0.84–65.17) | 1.37 (0.17–10.89) | 4.05 (0.51–32.27) | 0.96 (0.28–3.22) | 3.15 (0.41–24.33) | 4.03 (0.52–31.44) | 2.52 (0.34–18.76) | 6.40 (0.70–58.42) | 0.91 (0.23–3.59) | 0.75 (0.19–2.98) | **Risperidone** |  |  |  |  |  |
| 0.27 (0.09–0.78) | 1.01 (0.08–12.28) | 0.15 (0.05–0.45) | 0.08 (0.01–0.72) | 0.49 (0.06–4.19) | 0.22 (0.03–1.58) | 1.76 (0.26–11.83) | 0.17 (0.05–0.53) | 0.42 (0.11–1.60) | 0.08 (0.01–0.99) | 0.23 (0.07–0.74) | 0.05 (0.01–0.37) | 0.18 (0.06–0.54) | 0.23 (0.07–0.71) | 0.14 (0.05–0.41) | 0.36 (0.09–1.47) | 0.05 (0.01–0.31) | 0.04 (0.01–0.29) | 0.06 (0.01–0.54) | **rTMS** |  |  |  |  |
| 1.82 (0.24–13.55) | 6.83 (0.33–140.48) | 1.04 (0.14–7.68) | 0.51 (0.21–1.23) | 3.31 (0.21–51.32) | 1.50 (0.11–20.18) | 11.98 (0.63–229.58) | 1.13 (0.14–8.87) | 2.84 (0.33–24.70) | 0.53 (0.07–4.12) | 1.55 (0.20–12.23) | 0.37 (0.11–1.21) | 1.21 (0.16–9.22) | 1.54 (0.20–11.91) | 0.96 (0.13–7.10) | 2.45 (0.27–22.15) | 0.35 (0.09–1.35) | 0.29 (0.07–1.12) | 0.38 (0.15–0.95) | 6.79 (0.71–64.96) | **Sodium Valproate** |  |  |  |
| 0.21 (0.07–0.66) | 0.78 (0.06–9.86) | 0.12 (0.04–0.38) | 0.06 (0.01–0.58) | 0.38 (0.04–3.38) | 0.17 (0.02–1.28) | 1.37 (0.20–9.60) | 0.13 (0.04–0.44) | 0.32 (0.08–1.32) | 0.06 (0.00–0.79) | 0.18 (0.05–0.62) | 0.04 (0.01–0.30) | 0.14 (0.04–0.45) | 0.18 (0.05–0.59) | 0.11 (0.04–0.34) | 0.28 (0.06–1.21) | 0.04 (0.01–0.26) | 0.03 (0.00–0.23) | 0.04 (0.00–0.44) | 0.78 (0.51–1.18) | 0.11 (0.01–1.13) | **TBS** |  |  |
| 0.10 (0.00–15.80) | 0.37 (0.00–96.03) | 0.06 (0.00–9.08) | 0.03 (0.00–6.44) | 0.18 (0.00–40.13) | 0.08 (0.00–16.94) | 0.65 (0.00–162.18) | 0.06 (0.00–10.05) | 0.15 (0.00–26.28) | 0.03 (0.00–7.53) | 0.08 (0.00–13.79) | 0.02 (0.00–4.05) | 0.07 (0.00–10.60) | 0.08 (0.00–13.61) | 0.05 (0.00–8.34) | 0.13 (0.00–23.06) | 0.02 (0.00–3.73) | 0.02 (0.00–3.19) | 0.02 (0.00–4.86) | 0.37 (0.00–65.51) | 0.05 (0.00–12.65) | 0.48 (0.00–85.72) | **tDCS** |  |
| 2.88 (0.47–17.70) | 10.81 (0.59–196.46) | 1.65 (0.27–10.02) | 0.81 (0.34–1.89) | 5.25 (0.39–70.80) | 2.37 (0.20–27.63) | 18.97 (1.12–320.05) | 1.79 (0.28–11.65) | 4.49 (0.62–32.76) | 0.83 (0.13–5.42) | 2.46 (0.38–16.07) | 0.58 (0.25–1.34) | 1.91 (0.30–12.07) | 2.44 (0.38–15.62) | 1.53 (0.25–9.27) | 3.88 (0.51–29.47) | 0.55 (0.19–1.58) | 0.45 (0.16–1.31) | 0.61 (0.25–1.46) | 10.75 (1.33–86.84) | 1.58 (0.67–3.73) | 13.85 (1.65–116.29) | 29.07 (0.13–6309.87) | **Thyroid Hormone** |

*Remission was defined as a Montgomery-Asberg Depression Rating Scale (MADRS) score of ≤ 10 or a Hamilton Depression Rating Scale (HAMD) score of ≤ 7. Abbreviations: DBS: deep brain stimulation; ECT: electroconvulsive therapy; rTMS: repetitive transcranial magnetic therapy; TBS: theta burst stimulation; tDCS: transcranial direct current stimulation

### **Supplementary Figure S5.7. Ranking of included treatments for the remission* outcome**


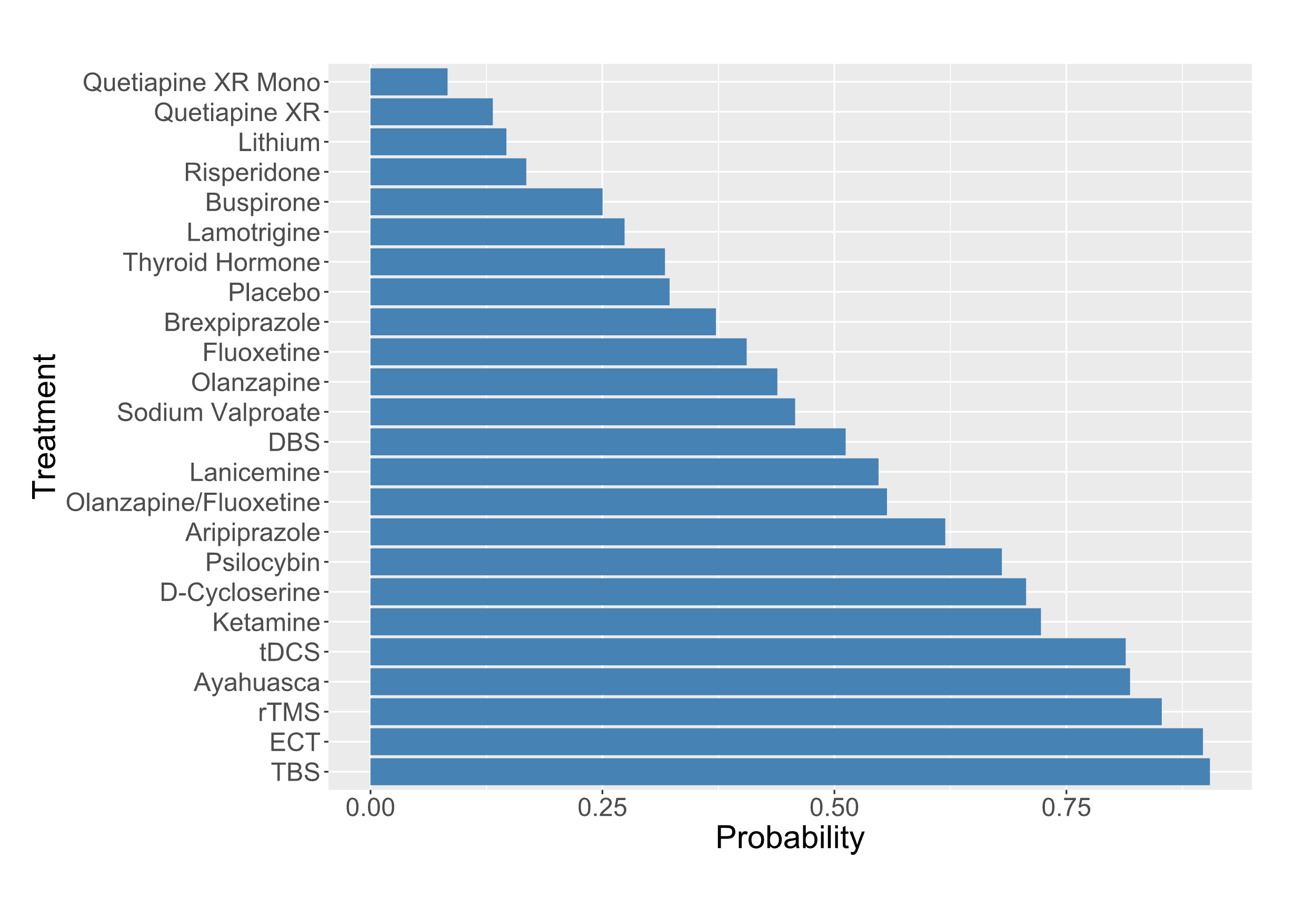


*Remission was defined as a Montgomery-Asberg Depression Rating Scale (MADRS) score of ≤ 10 or a Hamilton Depression Rating Scale (HAMD) score of ≤ 7. Abbreviations: DBS: deep brain stimulation; ECT: electroconvulsive therapy; rTMS: repetitive transcranial magnetic therapy; TBS: theta burst stimulation; tDCS: transcranial direct current stimulation; XR: extended release

### **Supplementary Figure S5.8. Network graph for the tolerance* outcome**


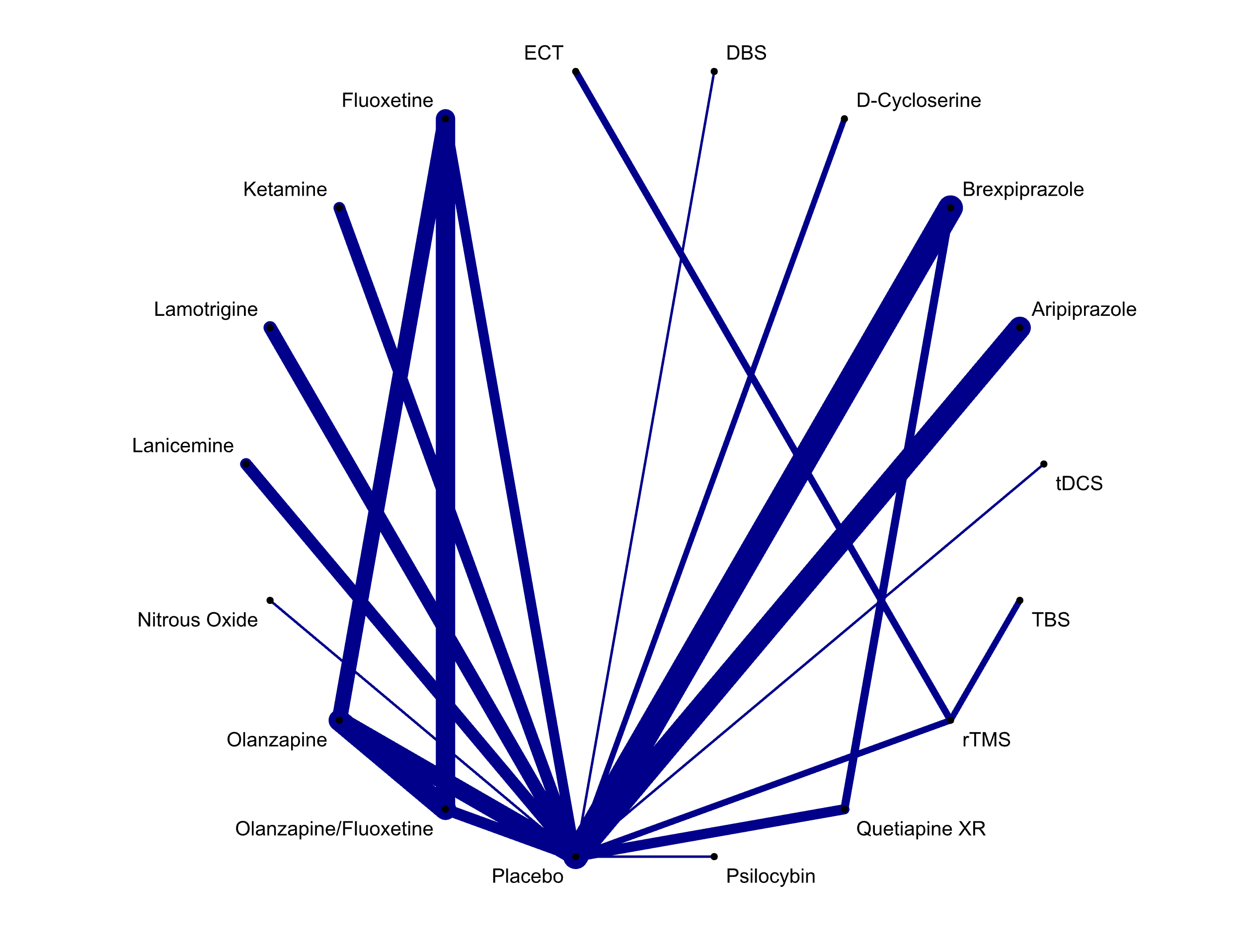


* Tolerance was defined as the proportion of participants who withdrew from a study due to adverse events. Abbreviations: DBS: deep brain stimulation; ECT: electroconvulsive therapy; rTMS: repetitive transcranial magnetic therapy; TBS: theta burst stimulation; tDCS: transcranial direct current stimulation; XR: extended release

### **Supplementary Figure S5.9. Forest plot for the tolerance* outcome**


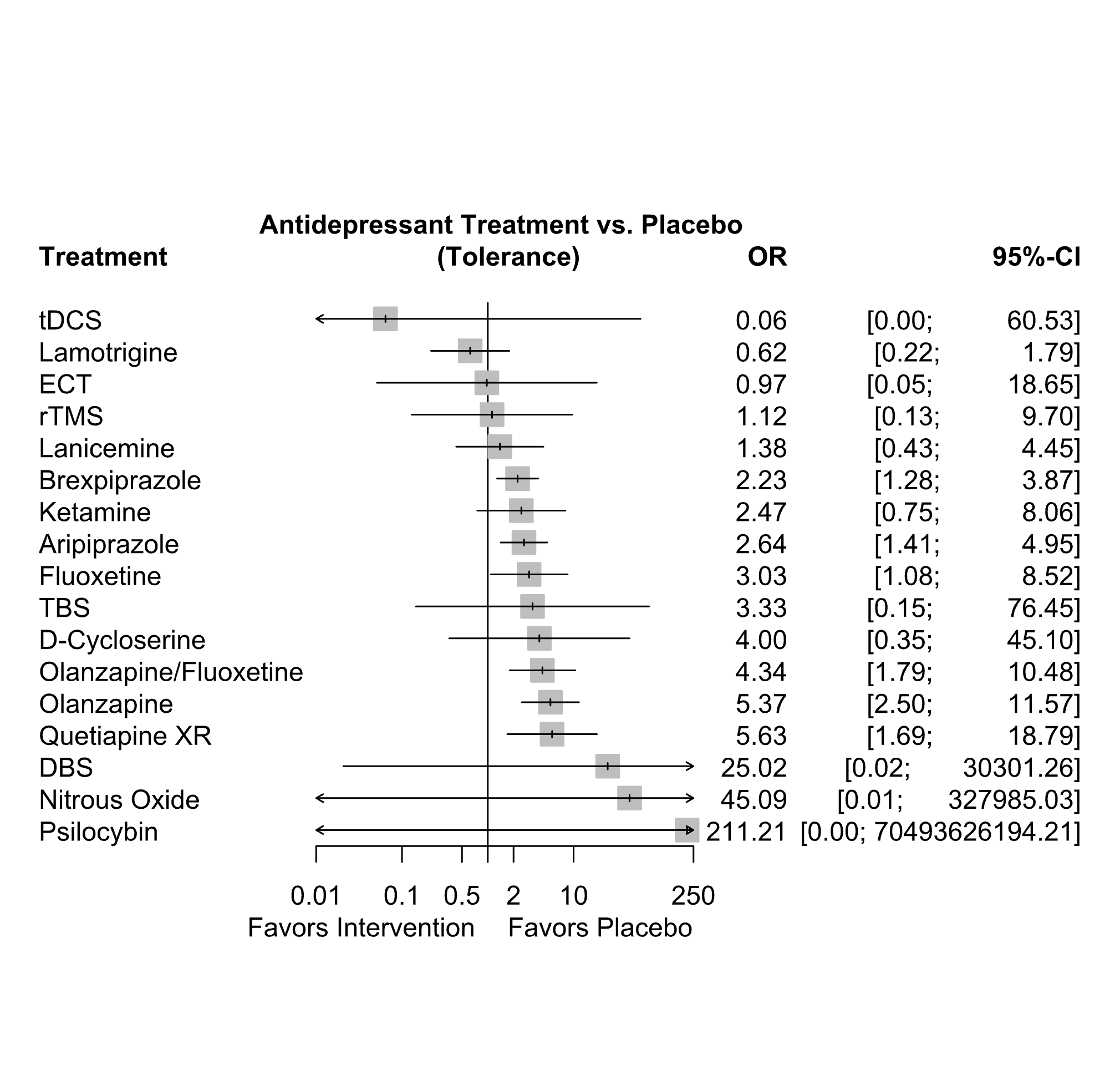


* Tolerance was defined as the proportion of participants who withdrew from a study due to adverse events. Abbreviations: CI: confidence interval; DBS: deep brain stimulation; ECT: electroconvulsive therapy; OR: odds ratio; rTMS: repetitive transcranial magnetic therapy; TBS: theta burst stimulation; tDCS: transcranial direct current stimulation; XR: extended release

### **Supplementary Table S5.4. League table for the tolerance* outcome**

| **Aripiprazole** |  |  |  |  |  |  |  |  |  |  |  |  |  |  |  |  |  |
| --- | --- | --- | --- | --- | --- | --- | --- | --- | --- | --- | --- | --- | --- | --- | --- | --- | --- |
| 1.19 (0.51-2.740000e+00) | **Brexpiprazole** |  |  |  |  |  |  |  |  |  |  |  |  |  |  |  |  |
| 0.66 (0.05-8.070000e+00) | 0.56 (0.05-6.690000e+00) | **D-Cycloserine** |  |  |  |  |  |  |  |  |  |  |  |  |  |  |  |
| 0.11 (0.00-1.314900e+02) | 0.09 (0.00-1.102000e+02) | 0.16 (0.00-2.893400e+02) | **DBS** |  |  |  |  |  |  |  |  |  |  |  |  |  |  |
| 2.71 (0.13-5.547000e+01) | 2.29 (0.11-4.610000e+01) | 4.11 (0.09-1.870100e+02) | 25.69 (0.01-5.606876e+04) | **ECT** |  |  |  |  |  |  |  |  |  |  |  |  |  |
| 0.87 (0.26-2.920000e+00) | 0.74 (0.23-2.380000e+00) | 1.32 (0.09-1.838000e+01) | 8.26 (0.01-1.077597e+04) | 0.32 (0.01-7.340000e+00) | **Fluoxetine** |  |  |  |  |  |  |  |  |  |  |  |  |
| 1.07 (0.28-4.090000e+00) | 0.90 (0.24-3.340000e+00) | 1.62 (0.11-2.405000e+01) | 10.15 (0.01-1.355396e+04) | 0.40 (0.02-9.510000e+00) | 1.23 (0.26-5.920000e+00) | **Ketamine** |  |  |  |  |  |  |  |  |  |  |  |
| 4.24 (1.25-1.446000e+01) | 3.58 (1.09-1.176000e+01) | 6.42 (0.46-9.013000e+01) | 40.17 (0.03-5.257627e+04) | 1.56 (0.07-3.593000e+01) | 4.87 (1.11-2.128000e+01) | 3.96 (0.81-1.932000e+01) | **Lamotrigine** |  |  |  |  |  |  |  |  |  |  |
| 1.91 (0.51-7.210000e+00) | 1.61 (0.44-5.880000e+00) | 2.89 (0.20-4.265000e+01) | 18.10 (0.01-2.412714e+04) | 0.70 (0.03-1.687000e+01) | 2.19 (0.46-1.045000e+01) | 1.78 (0.34-9.430000e+00) | 0.45 (0.09-2.180000e+00) | **Lanicemine** |  |  |  |  |  |  |  |  |  |
| 0.06 (0.00-4.359000e+02) | 0.05 (0.00-3.657900e+02) | 0.09 (0.00-8.923000e+02) | 0.55 (0.00-4.850821e+04) | 0.02 (0.00-2.532800e+02) | 0.07 (0.00-5.190800e+02) | 0.05 (0.00-4.303500e+02) | 0.01 (0.00-1.069300e+02) | 0.03 (0.00-2.407500e+02) | **Nitrous Oxide** |  |  |  |  |  |  |  |  |
| 0.49 (0.18-1.320000e+00) | 0.41 (0.16-1.070000e+00) | 0.74 (0.06-9.450000e+00) | 4.66 (0.00-5.875100e+03) | 0.18 (0.01-3.830000e+00) | 0.56 (0.25-1.270000e+00) | 0.46 (0.11-1.880000e+00) | 0.12 (0.03-4.300000e-01) | 0.26 (0.06-1.040000e+00) | 8.39 (0.00-6.306786e+04) | **Olanzapine** |  |  |  |  |  |  |  |
| 0.61 (0.21-1.800000e+00) | 0.51 (0.18-1.460000e+00) | 0.92 (0.07-1.215000e+01) | 5.77 (0.00-7.377370e+03) | 0.22 (0.01-4.890000e+00) | 0.70 (0.34-1.420000e+00) | 0.57 (0.13-2.490000e+00) | 0.14 (0.04-5.700000e-01) | 0.32 (0.07-1.380000e+00) | 10.39 (0.00-7.898354e+04) | 1.24 (0.68-2.270000e+00) | **Olanzapine/Fluoxetine** |  |  |  |  |  |  |
| 2.64 (1.41-4.950000e+00) | 2.23 (1.28-3.870000e+00) | 4.00 (0.35-4.510000e+01) | 25.02 (0.02-3.030126e+04) | 0.97 (0.05-1.865000e+01) | 3.03 (1.08-8.520000e+00) | 2.47 (0.75-8.060000e+00) | 0.62 (0.22-1.790000e+00) | 1.38 (0.43-4.450000e+00) | 45.09 (0.01-3.279850e+05) | 5.37 (2.50-1.157000e+01) | 4.34 (1.79-1.048000e+01) | **Placebo** |  |  |  |  |  |
| 0.01 (0.00-4.218777e+06) | 0.01 (0.00-3.549681e+06) | 0.02 (0.00-7.336469e+06) | 0.12 (0.00-1.372646e+08) | 0.00 (0.00-1.919873e+06) | 0.01 (0.00-4.921269e+06) | 0.01 (0.00-4.038862e+06) | 0.00 (0.00-1.012561e+06) | 0.01 (0.00-2.261775e+06) | 0.21 (0.00-4.862397e+08) | 0.03 (0.00-8.622208e+06) | 0.02 (0.00-6.992595e+06) | 0.00 (0.00-1.580292e+06) | **Psilocybin** |  |  |  |  |
| 0.47 (0.12-1.830000e+00) | 0.40 (0.11-1.370000e+00) | 0.71 (0.05-1.063000e+01) | 4.44 (0.00-5.955240e+03) | 0.17 (0.01-4.190000e+00) | 0.54 (0.11-2.630000e+00) | 0.44 (0.08-2.370000e+00) | 0.11 (0.02-5.500000e-01) | 0.25 (0.05-1.320000e+00) | 8.01 (0.00-6.316730e+04) | 0.95 (0.23-3.980000e+00) | 0.77 (0.17-3.430000e+00) | 0.18 (0.05-5.900000e-01) | 37.50 (0.00-1.298804e+10) | **Quetiapine XR** |  |  |  |
| 2.36 (0.25-2.228000e+01) | 1.99 (0.21-1.843000e+01) | 3.57 (0.14-9.141000e+01) | 22.31 (0.01-3.721980e+04) | 0.87 (0.12-6.510000e+00) | 2.70 (0.25-2.955000e+01) | 2.20 (0.19-2.576000e+01) | 0.56 (0.05-6.130000e+00) | 1.23 (0.11-1.434000e+01) | 40.19 (0.00-3.784219e+05) | 4.79 (0.49-4.730000e+01) | 3.87 (0.38-3.979000e+01) | 0.89 (0.10-7.710000e+00) | 188.27 (0.00-7.072454e+10) | 5.02 (0.42-5.942000e+01) | **rTMS** |  |  |
| 0.79 (0.03-1.936000e+01) | 0.67 (0.03-1.610000e+01) | 1.20 (0.02-6.297000e+01) | 7.51 (0.00-1.759968e+04) | 0.29 (0.01-6.090000e+00) | 0.91 (0.03-2.463000e+01) | 0.74 (0.03-2.107000e+01) | 0.19 (0.01-5.090000e+00) | 0.41 (0.01-1.175000e+01) | 13.53 (0.00-1.681527e+05) | 1.61 (0.06-4.058000e+01) | 1.30 (0.05-3.373000e+01) | 0.30 (0.01-6.880000e+00) | 63.37 (0.00-2.711708e+10) | 1.69 (0.06-4.848000e+01) | 0.34 (0.03-3.260000e+00) | **TBS** |  |
| 41.19 (0.04-3.998140e+04) | 34.73 (0.04-3.350174e+04) | 62.33 (0.04-8.911686e+04) | 389.94 (0.02-7.502118e+06) | 15.18 (0.01-2.633178e+04) | 47.23 (0.05-4.814303e+04) | 38.43 (0.04-4.013120e+04) | 9.71 (0.01-9.924340e+03) | 21.54 (0.02-2.243889e+04) | 702.65 (0.01-5.264500e+07) | 83.76 (0.09-8.246691e+04) | 67.60 (0.07-6.748320e+04) | 15.58 (0.02-1.470006e+04) | 3291.29 (0.00-3.507304e+12) | 87.76 (0.08-9.197103e+04) | 17.48 (0.01-2.297937e+04) | 51.94 (0.03-9.694299e+04) | **tDCS** |

*Tolerance was defined as the proportion of participants who withdrew from a study due to adverse events. Abbreviations: DBS: deep brain stimulation; ECT: electroconvulsive therapy; rTMS: repetitive transcranial magnetic therapy; TBS: theta burst stimulation; tDCS: transcranial direct current stimulation

### **Supplementary Figure S5.10 Ranking of included treatments for the tolerance* outcome**

**
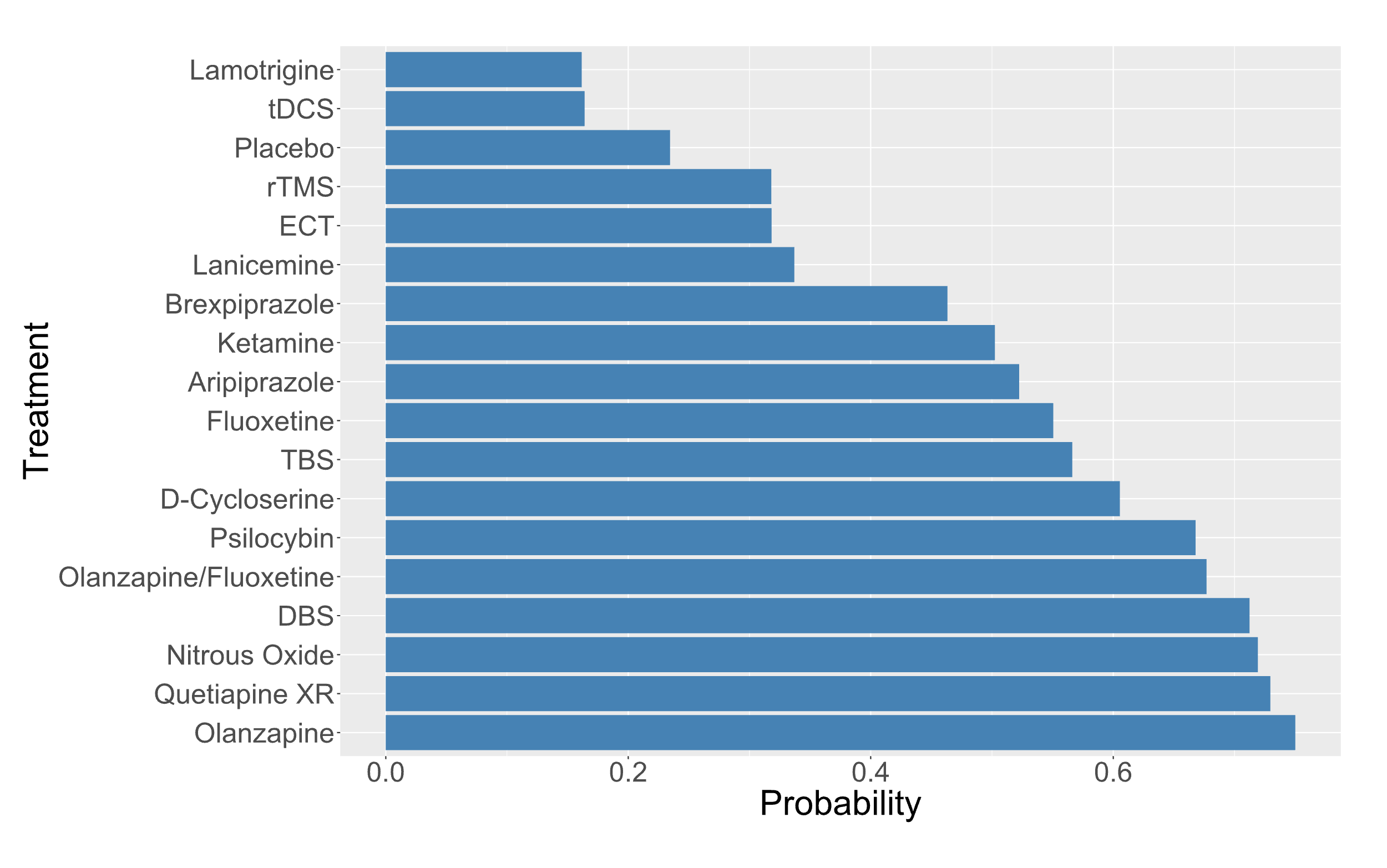
**

*Tolerance was defined as the proportion of participants who withdrew from a study due to adverse events. Abbreviations: DBS: deep brain stimulation; ECT: electroconvulsive therapy; rTMS: repetitive transcranial magnetic therapy; TBS: theta burst stimulation; tDCS: transcranial direct current stimulation; XR: extended release

# 6. Group-level analysis

## **Supplementary Figure S6.1 Network graph for group-level response rate outcome**


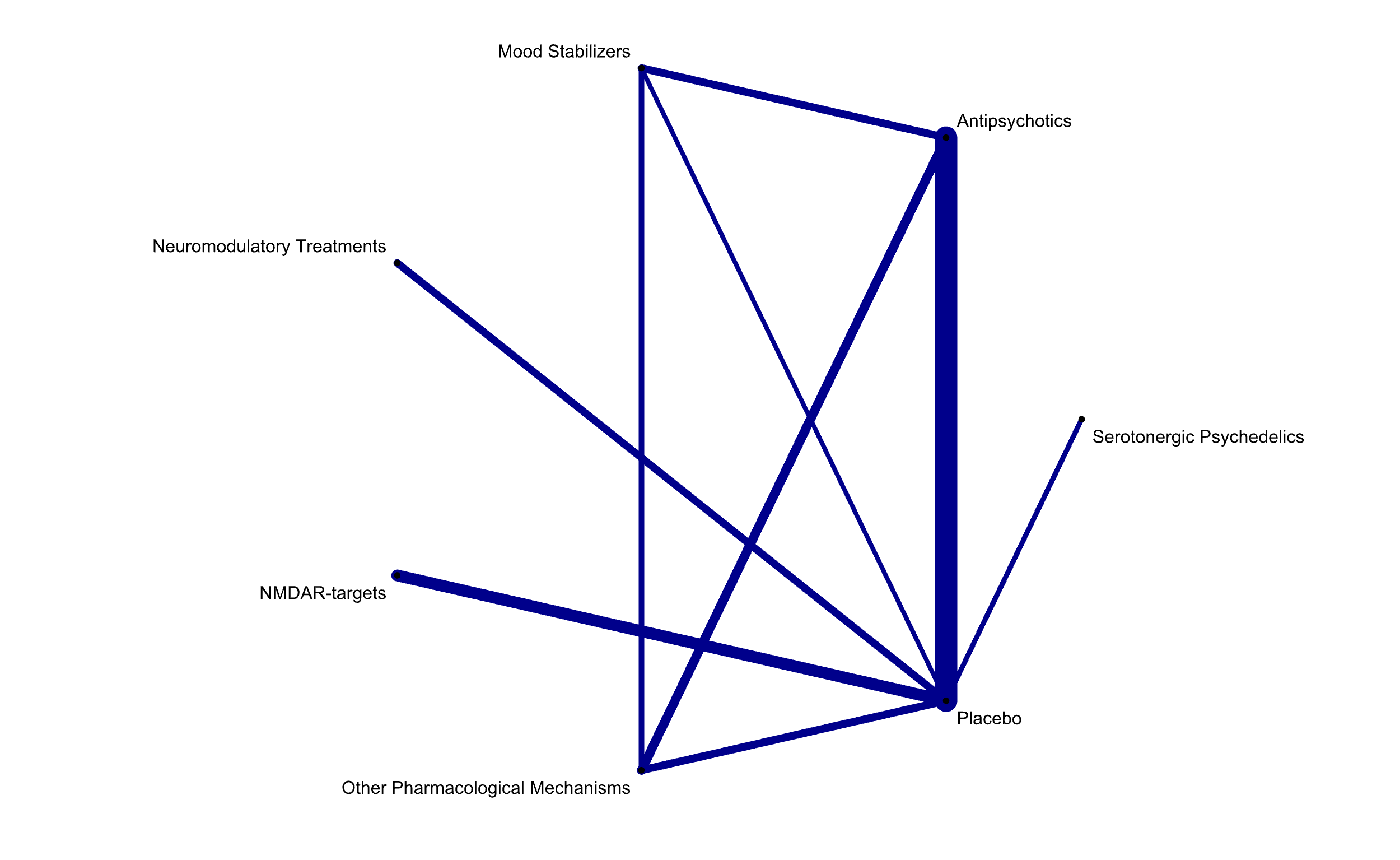


Abbreviations: NMDAR: N-methyl-D-aspartate receptor

## **Supplementary Table S6. League table for the group-level response rate outcome**

| **Antipsychotics** |  |  |  |  |  |  |
| --- | --- | --- | --- | --- | --- | --- |
| 1.25 (0.73-2.13) | **Mood Stabilizers** |  |  |  |  |  |
| 0.41 (0.24-0.70) | 0.33 (0.16-0.67) | **Neuromodulatory Treatments** |  |  |  |  |
| 0.46 (0.29-0.73) | 0.37 (0.19-0.72) | 1.14 (0.62-2.07) | **N-methyl-D-aspartate Receptor Targets** |  |  |  |
| 0.95 (0.58-1.56) | 0.76 (0.42-1.39) | 2.33 (1.17-4.66) | 2.05 (1.09-3.86) | **Other Pharmacological Mechanism** |  |  |
| 1.36 (1.04-1.78) | 1.09 (0.63-1.88) | 3.35 (2.09-5.35) | 2.94 (2.03-4.27) | 1.44 (0.86-2.39) | **Placebo** |  |
| 0.60 (0.23-1.56) | 0.48 (0.17-1.39) | 1.48 (0.53-4.13) | 1.30 (0.49-3.48) | 0.64 (0.22-1.81) | 0.44 (0.18-1.10) | **Serotonergic Psychedelics** |

## **Supplementary Figure S6.2. Ranking of included treatments for group-level response rate outcome**

**
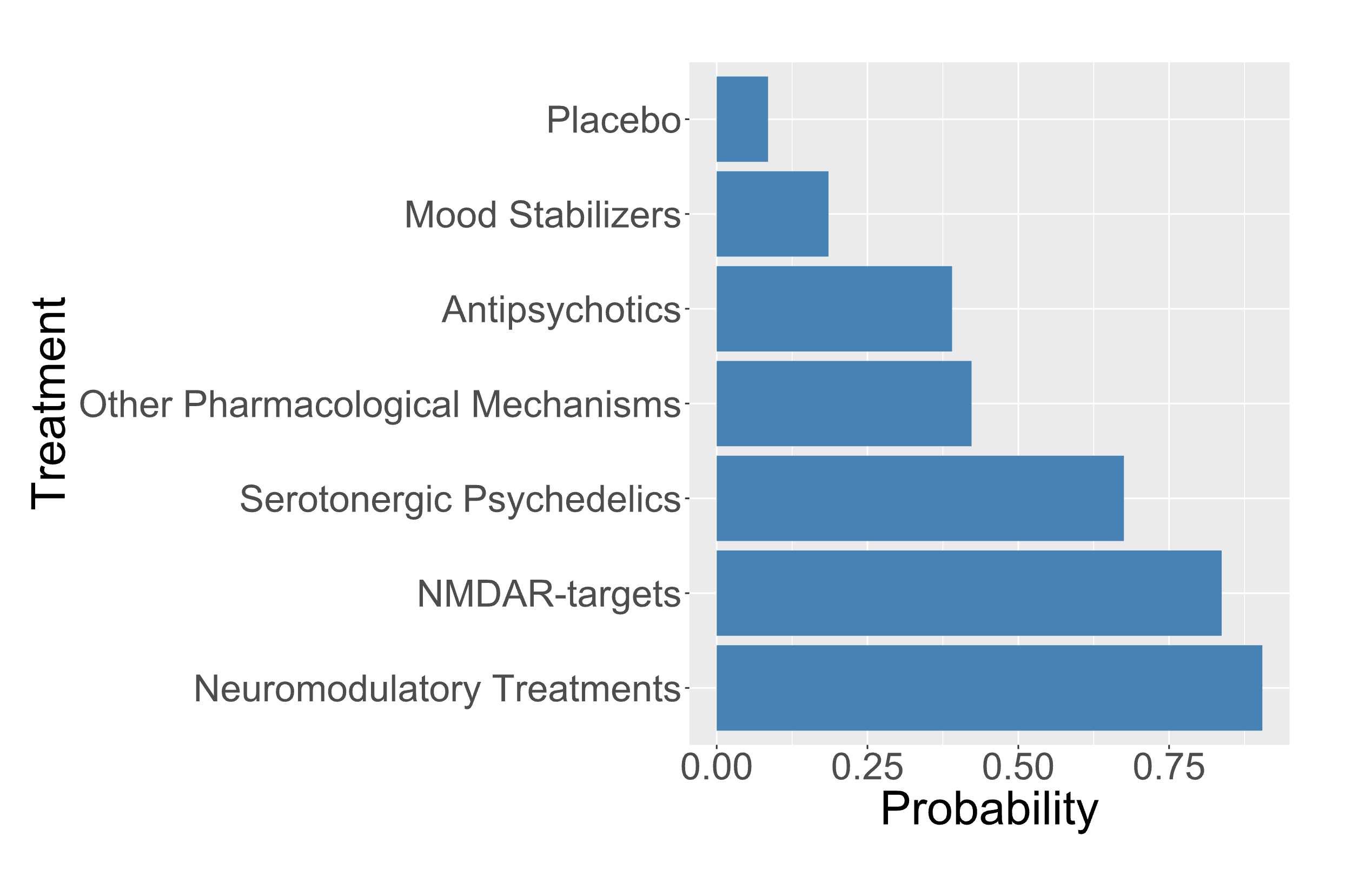
**

Abbreviations: NMDAR: N-methyl-D-aspartate receptor

# 7. Analysis of inconsistency

## **Supplementary Figure S7.1. Netheat plot for network meta-analysis of outcome response rate**


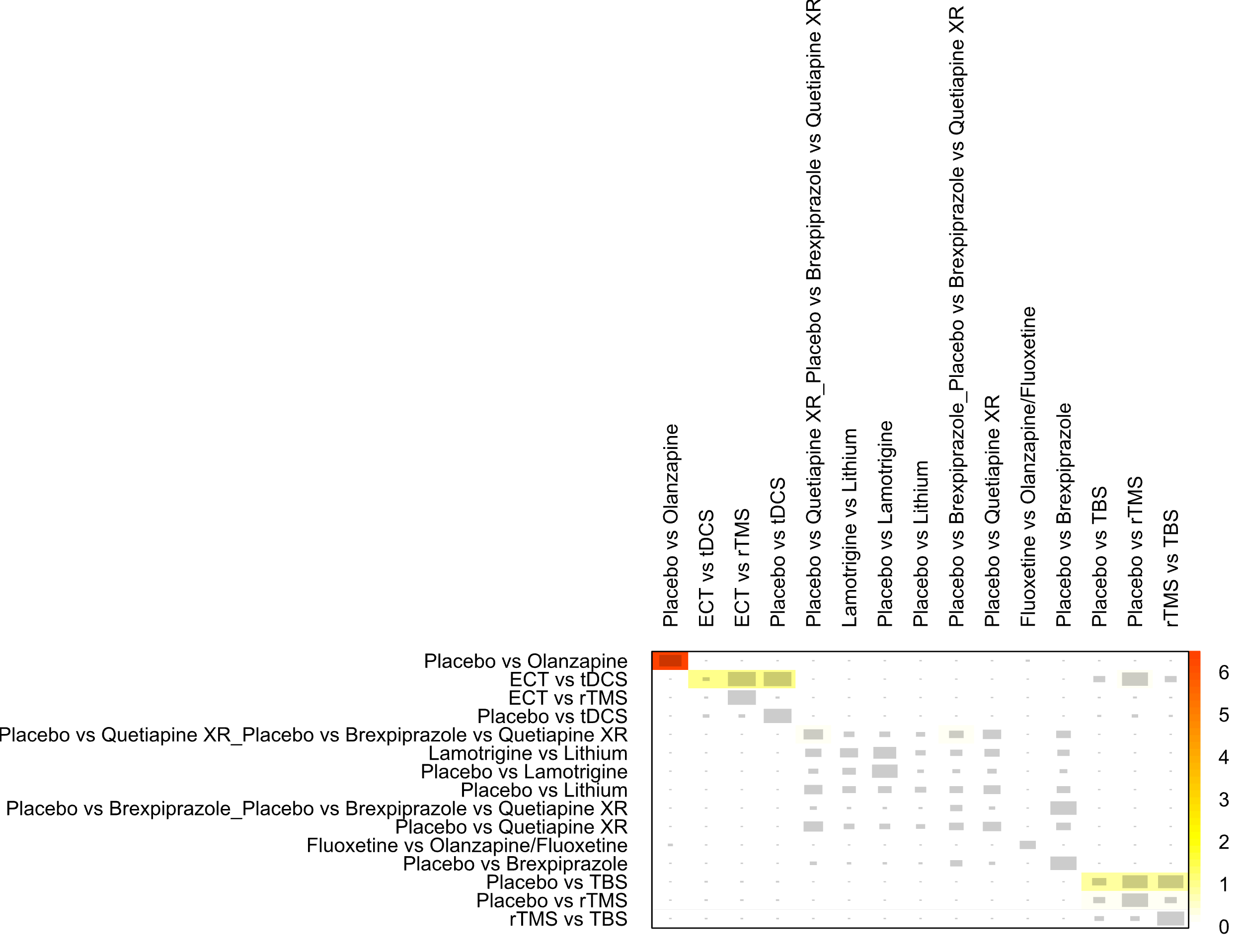


Netheat map (random effects model) showing the contribution of different designs to the total heterogeneity. The size of the grey boxes indicates how important one treatment comparison was for estimating the size of the other. For example, the comparison of standardized mean differences for estimating the comparison of placebo versus TBS stems in large part from indirect evidence drawn from studies that compared this treatment with rTMS or placebo with rTMS. The color indicates how much of the inconsistency of a treatment comparison in a row was attributable to the comparison in the column. Here, for example, the indirect evidence estimating the comparison of TBS and placebo is largely inconsistent with the direct evidence.

Abbreviations: DBS: deep brain stimulation; ECT: electroconvulsive therapy; rTMS: repetitive transcranial magnetic therapy; TBS: theta burst stimulation; tDCS: transcranial direct current stimulation; XR: extended release

## **Supplementary Figure S7.2. Netheat plot for network meta-analysis of the standardized mean difference outcome**

##

**
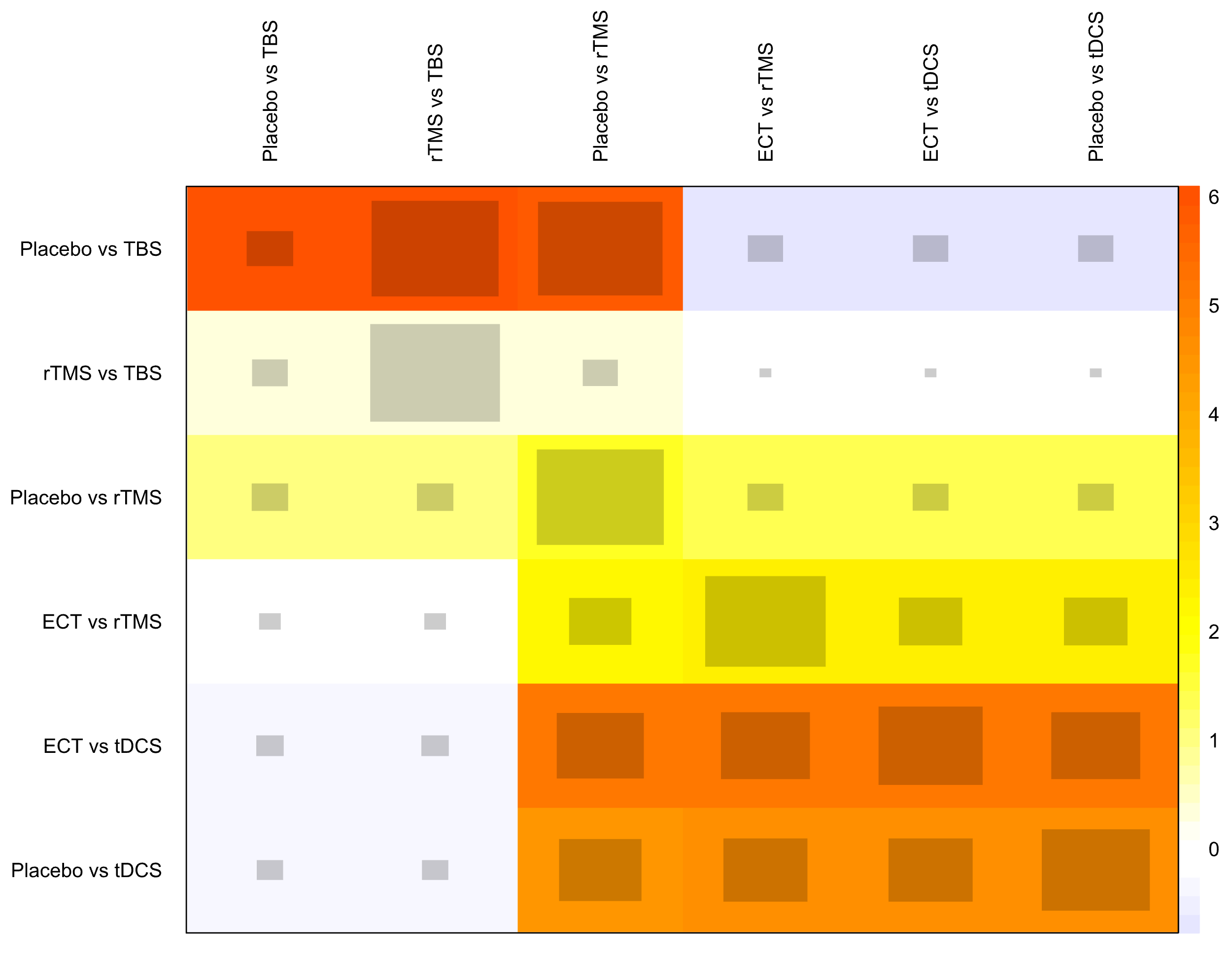
**

Abbreviations: ECT: electroconvulsive therapy; rTMS: repetitive transcranial magnetic therapy; TBS: theta burst stimulation; tDCS: transcranial direct current stimulation

## **Supplementary Figure S7.3. Netheat plot for network meta-analysis of the remission outcome**


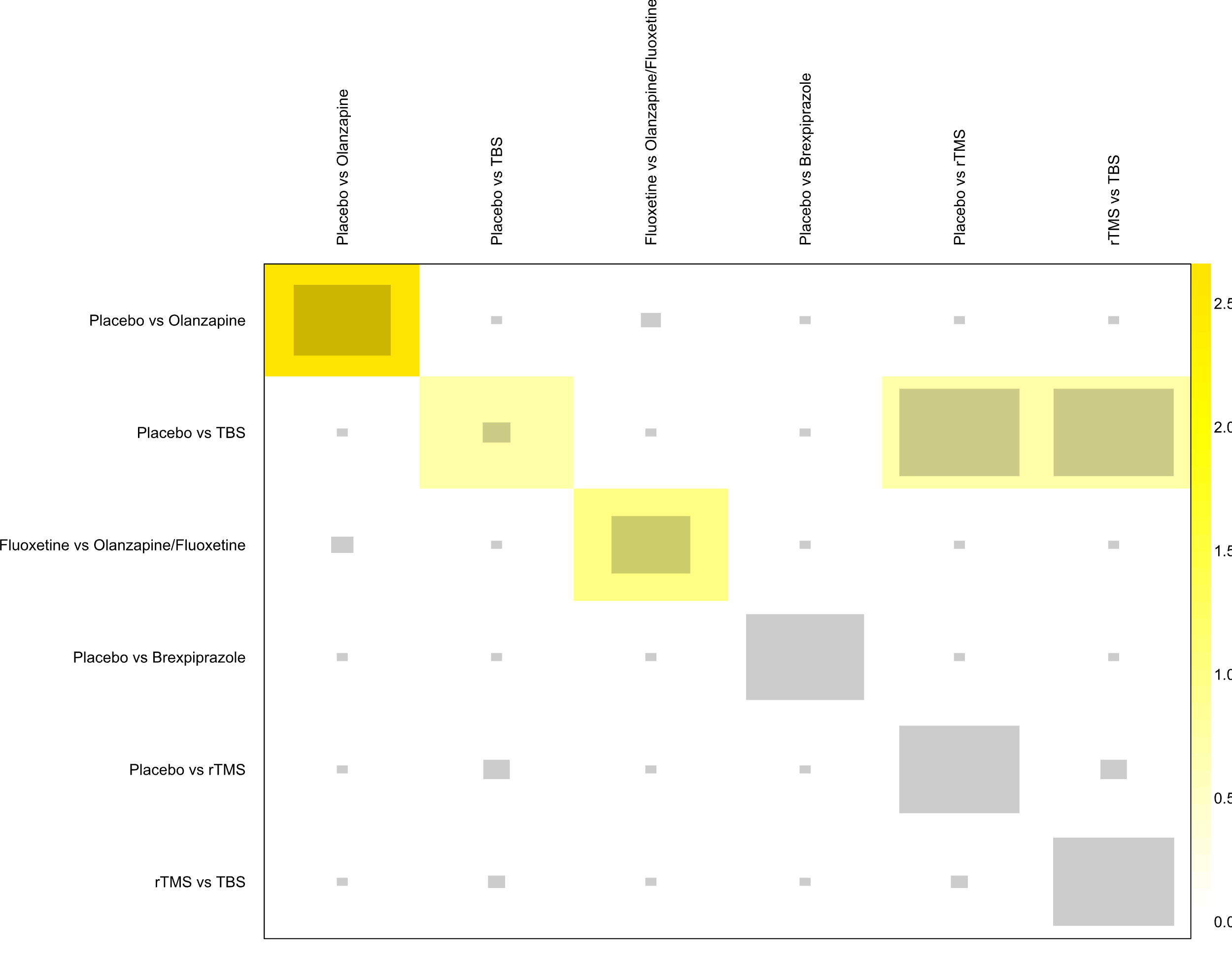


Abbreviations: rTMS: repetitive transcranial magnetic therapy; TBS: theta burst stimulation

## **Supplementary Figure S7.4. Netheat plot of network meta-analysis of the tolerance outcome**


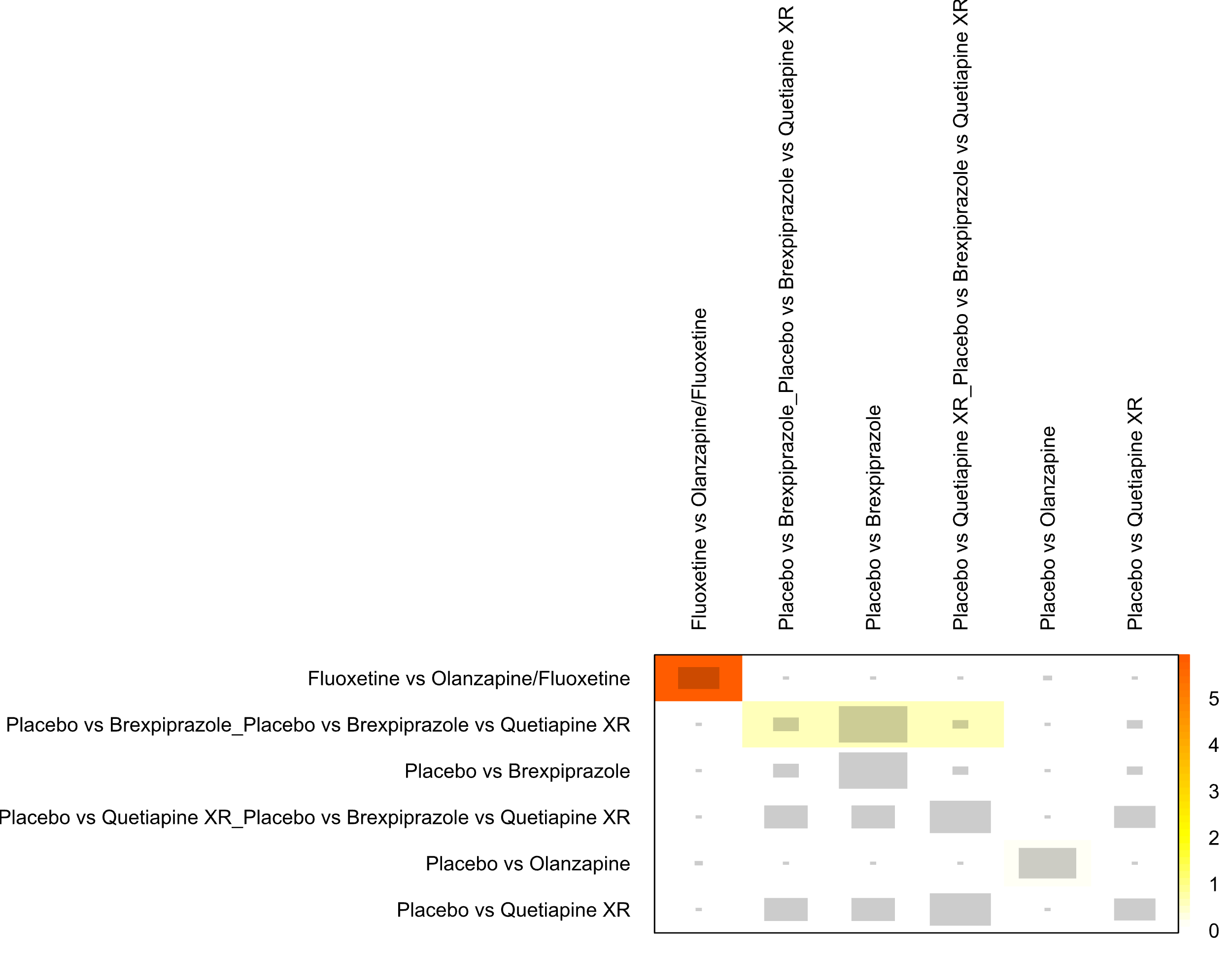


Abbreviations: XR: extended release

# 8. Pairwise Meta-Analysis

## **Supplementary Figure S8.1. Forest plot for pairwise meta-analysis of response rate outcome**


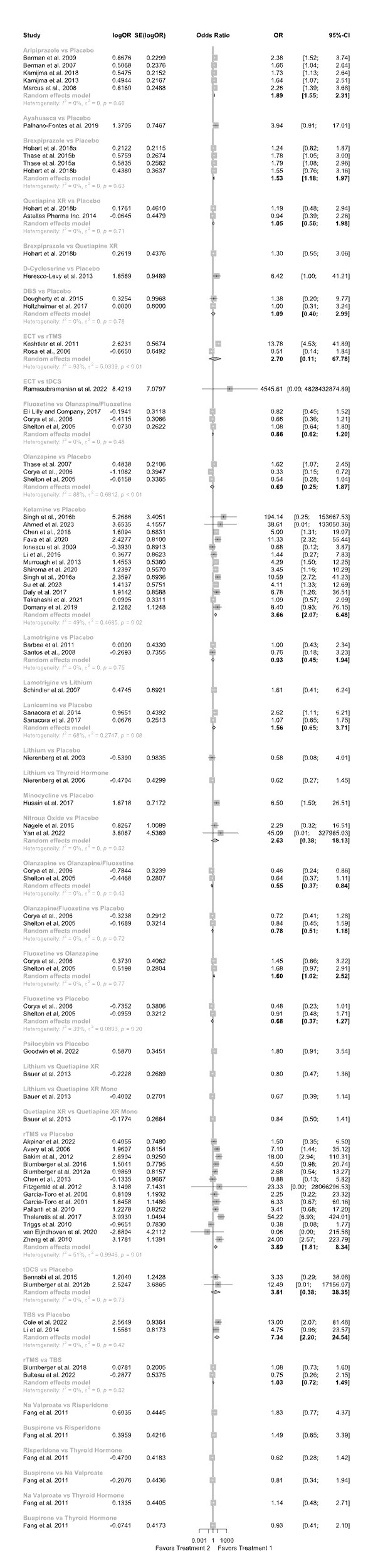


**
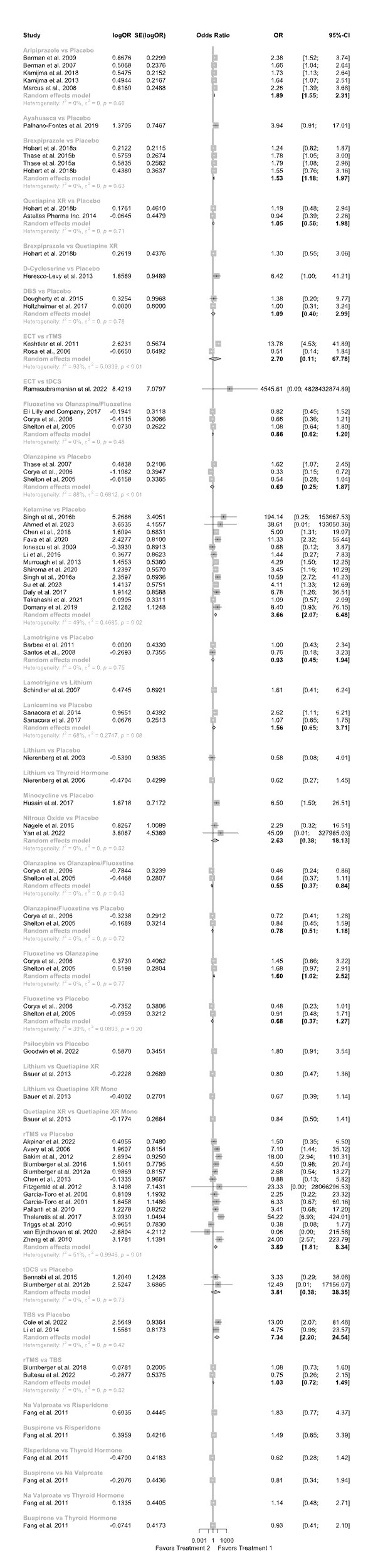
**

**
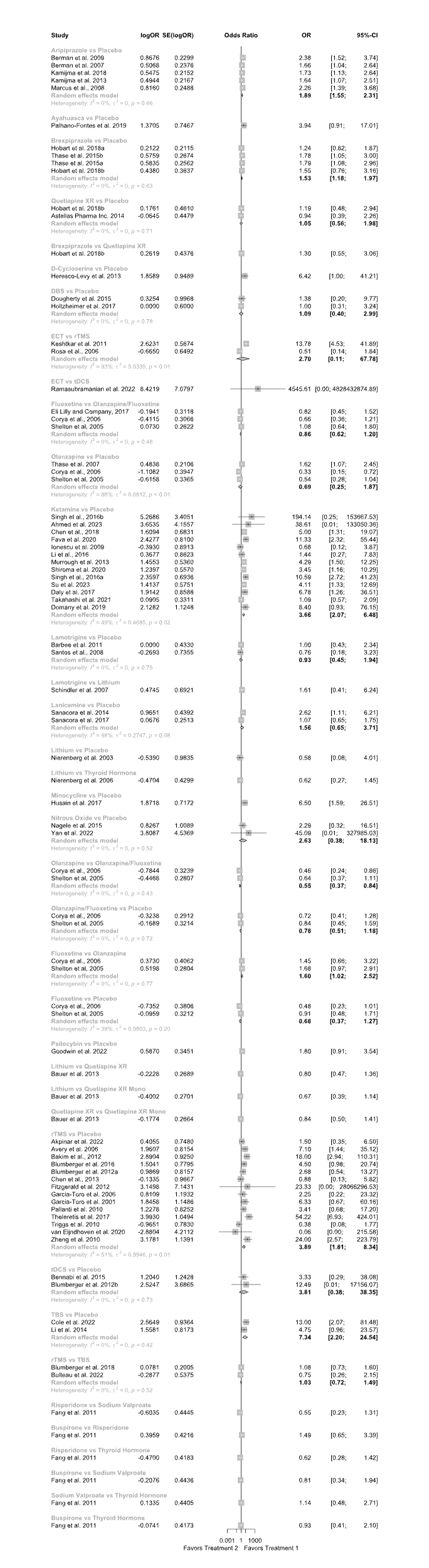
**

Abbreviations: CI: confidence interval; DBS: deep brain stimulation; ECT: electroconvulsive therapy; OR: odds ratio; rTMS: repetitive transcranial magnetic therapy; TBS: theta burst stimulation; tDCS: transcranial direct current stimulation; XR: extended release

## **Supplementary Figure S8.2. Forest plot for pairwise meta-analysis for the standardized mean difference outcome**


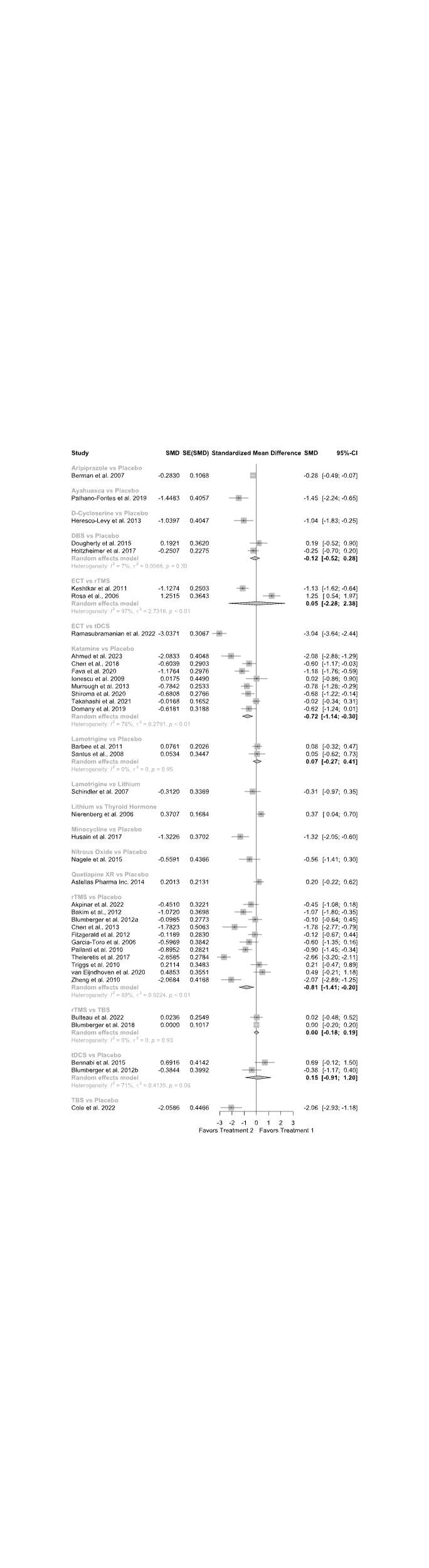


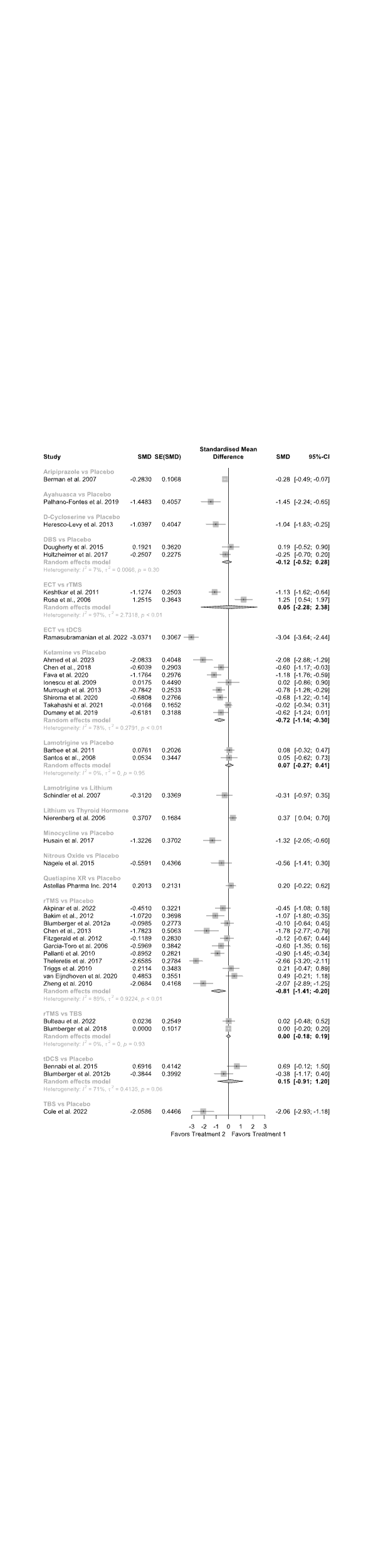


Abbreviations: CI: confidence interval; DBS: deep brain stimulation; ECT: electroconvulsive therapy; OR: odds ratio; SE: standard error; SMD: standardized mean difference; rTMS: repetitive transcranial magnetic therapy; TBS: theta burst stimulation; tDCS: transcranial direct current stimulation; XR: extended release

## **Supplementary Figure S8.3. Forest plot for pairwise meta-analysis for the remission outcome**


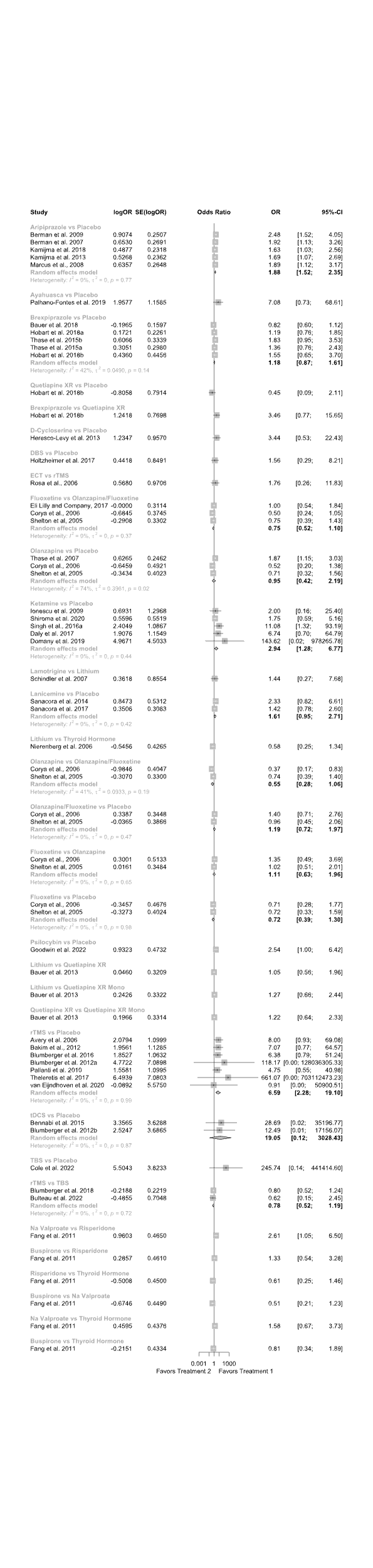


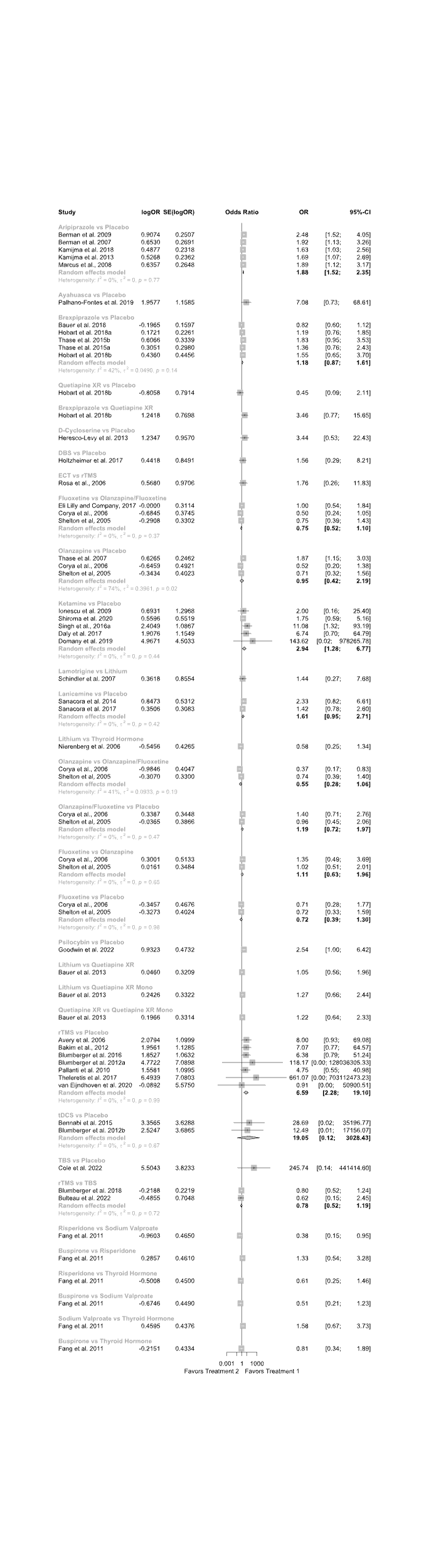


Abbreviations: CI: confidence interval; DBS: deep brain stimulation; ECT: electroconvulsive therapy; OR: odds ratio; rTMS: repetitive transcranial magnetic therapy; TBS: theta burst stimulation; tDCS: transcranial direct current stimulation; XR: extended release

## **Supplementary Figure S8.4. Forest plot for pairwise meta-analysis for the tolerance outcome**


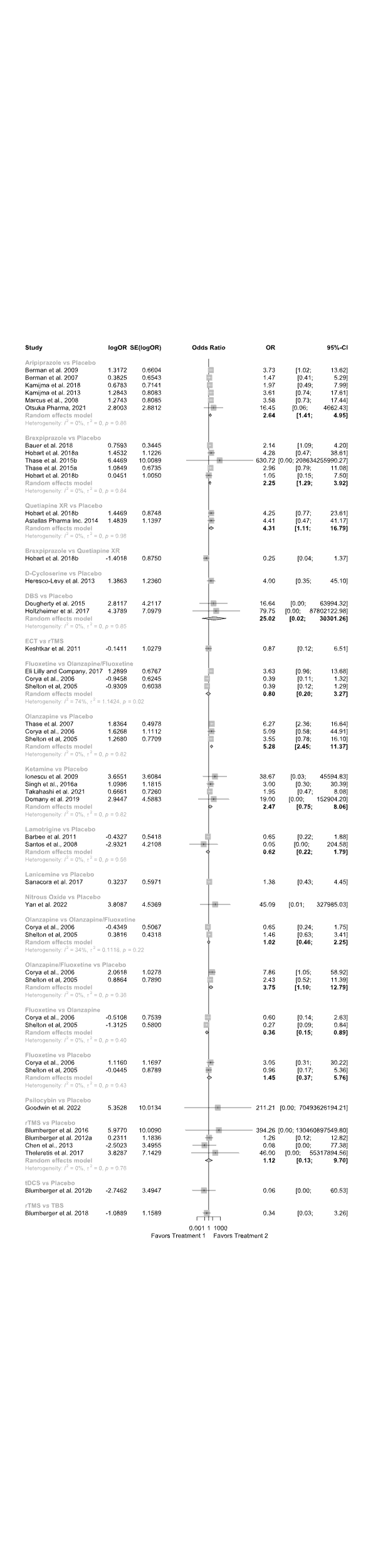


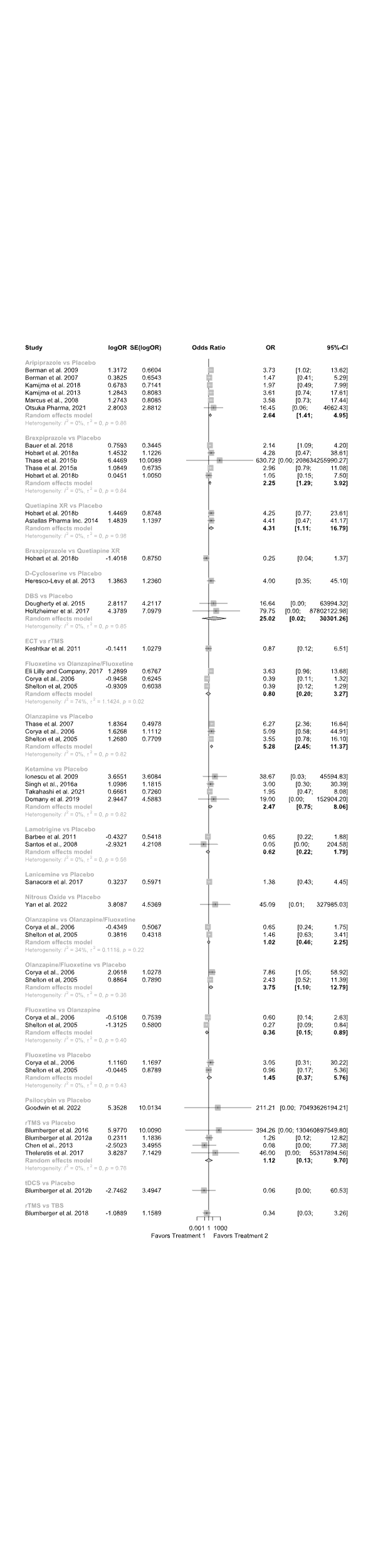


Abbreviations: CI: confidence interval; DBS: deep brain stimulation; ECT: electroconvulsive therapy; OR: odds ratio; rTMS: repetitive transcranial magnetic therapy; SE: standard error; TBS: theta burst stimulation; tDCS: transcranial direct current stimulation; XR: extended release

# 9. Bayesian Network Meta-Analysis

## **Supplementary Figure S9.1. Forest plot of estimated effect sizes for the response rate outcome**


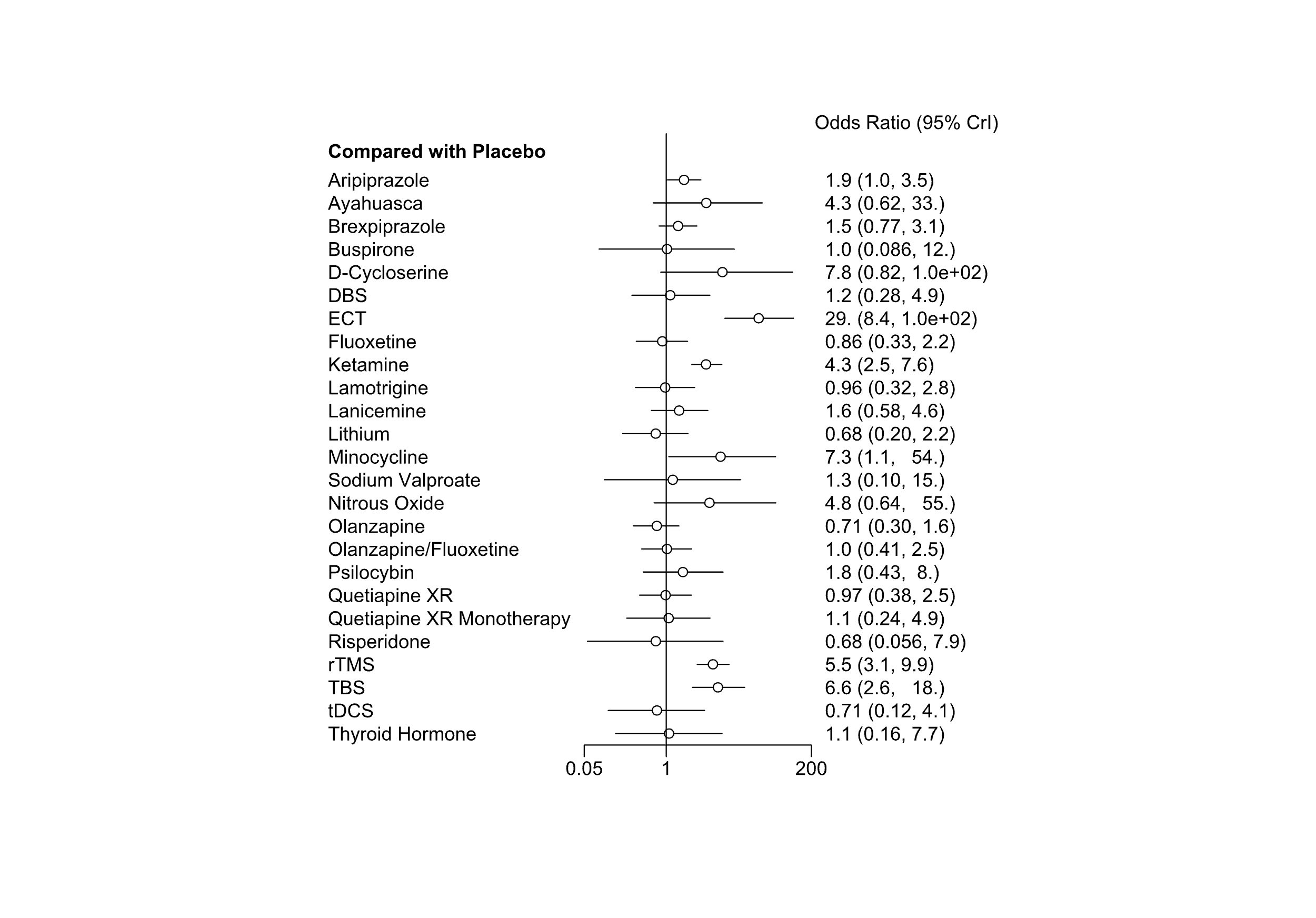


Abbreviations: Crl: credible interval; DBS: deep brain stimulation; ECT: electroconvulsive therapy; rTMS: repetitive transcranial magnetic therapy; TBS: theta burst stimulation; tDCS: transcranial direct current stimulation; XR: extended release

## **Supplementary Figure S9.2. Forest plot of a nodesplit for the response rate outcome**


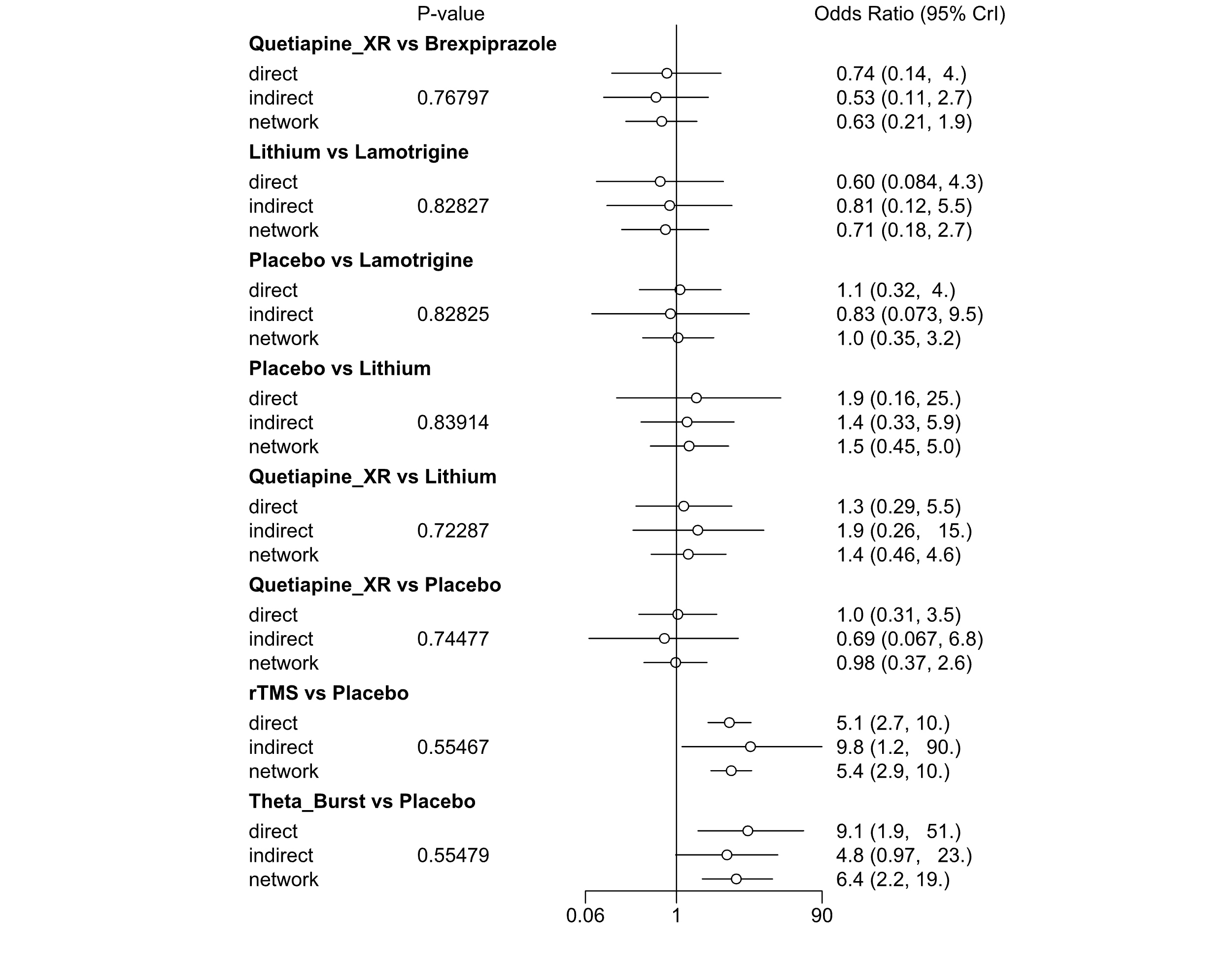


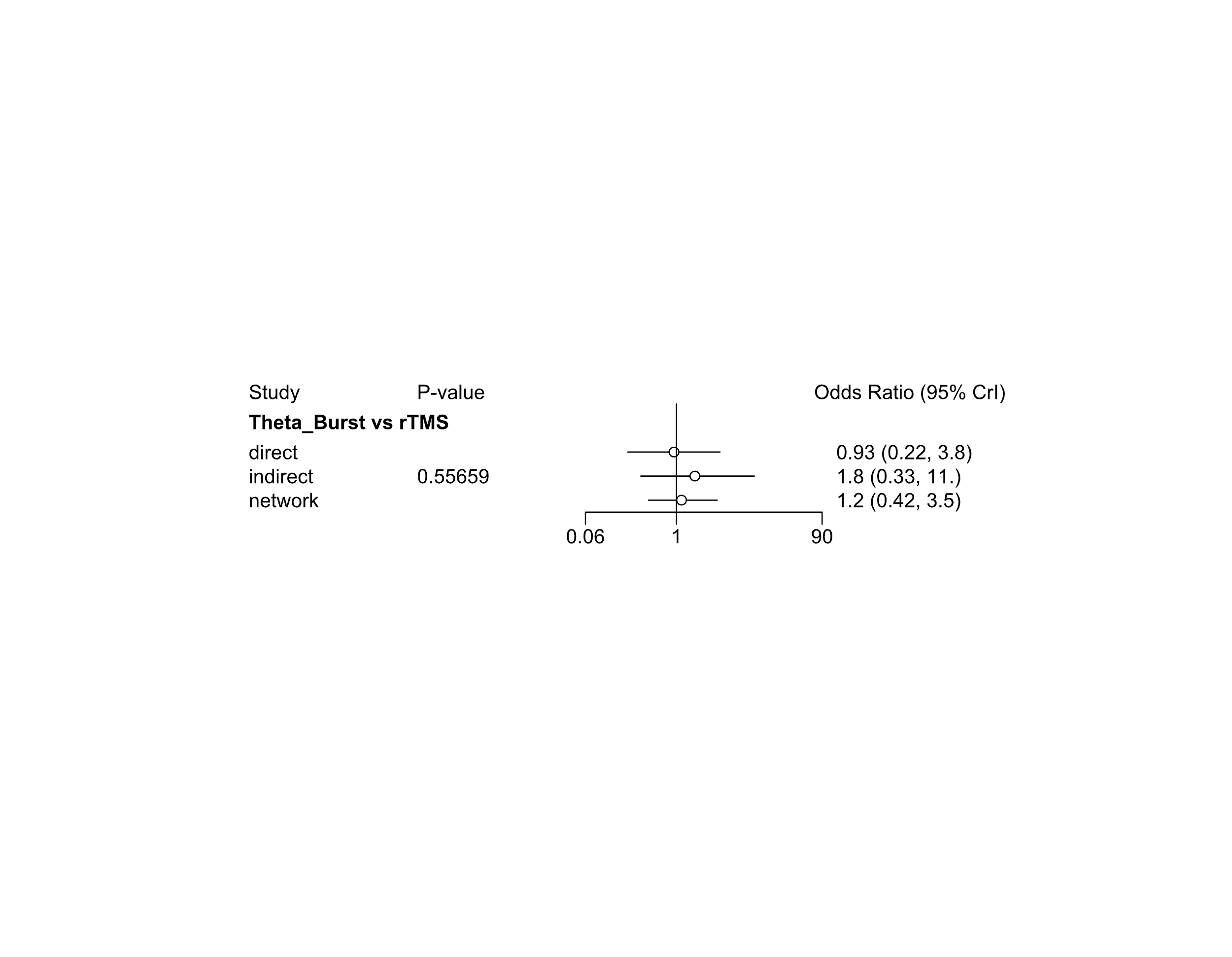


The analysis compares the evaluated effect when using only direct, indirect, or combined evidence. A p<0.05 in one or more comparisons indicates inconsistency in the network. No indication of inconsistency was found in our network, as no comparison showed a p<0.05.

Abbreviations: Crl: credible interval; rTMS: repetitive transcranial magnetic therapy; XR: extended release

## **Supplementary Figure S9.3. Ranking of included treatments for response rate outcome**


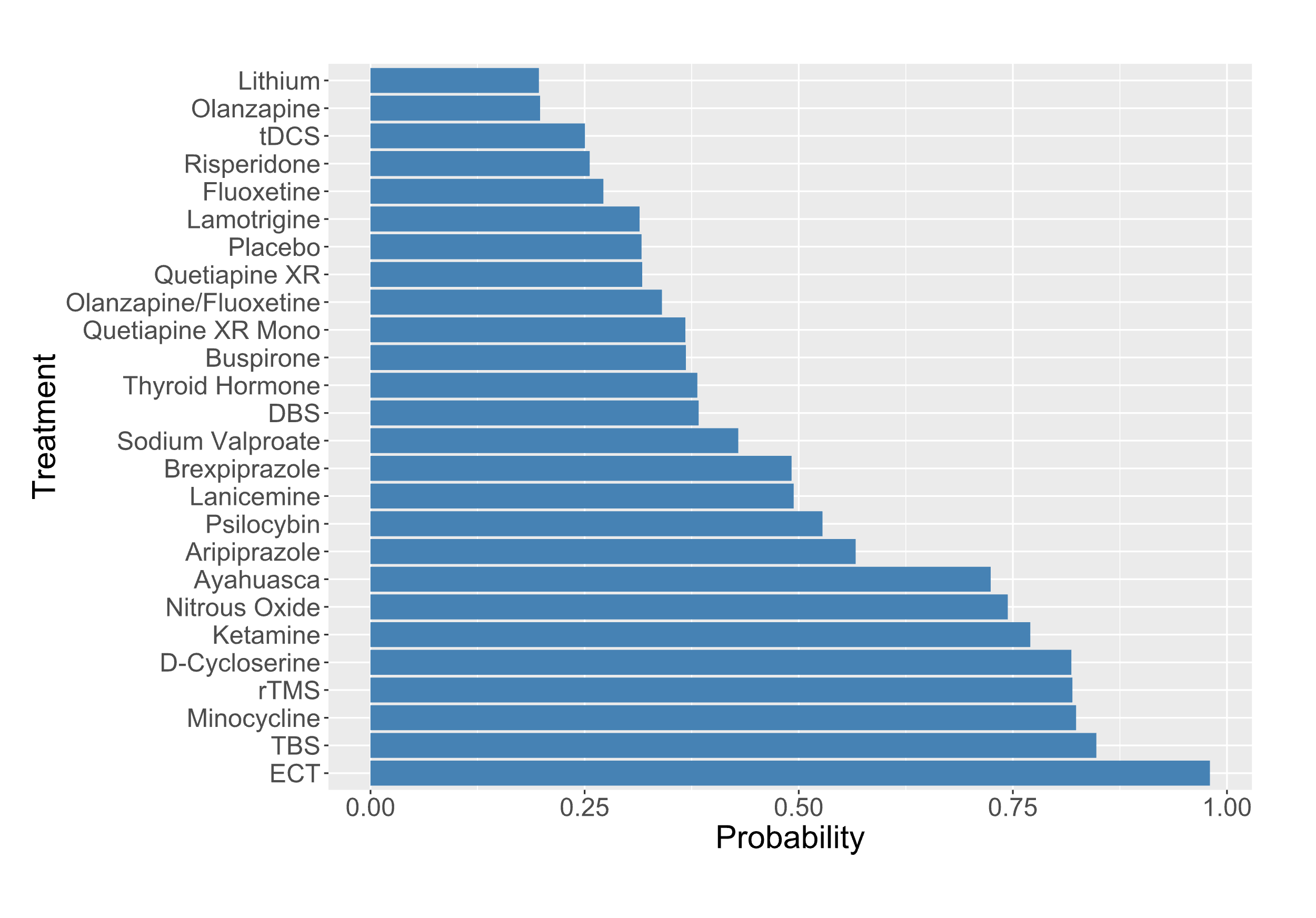


The surface under the cumulative ranking (SUCRA) score is shown on the y-axis. This score is equivalent to the p-score and may be used to estimate which treatment had the highest cumulative probability of being the most effective. Abbreviations: DBS: deep brain stimulation; ECT: electroconvulsive therapy; rTMS: repetitive transcranial magnetic therapy; TBS: theta burst stimulation; tDCS: transcranial direct current stimulation; XR: extended release

# 10. Scenario Analyses

The following scenario analyses evaluated the robustness of the main outcome (response rate) by only analyzing a certain subset of included studies (e.g., excluding studies without a placebo arm).

## **Sham vs. Placebo**

### **Supplementary Figure S10.1. Forest plot excluding studies using sham conditions**


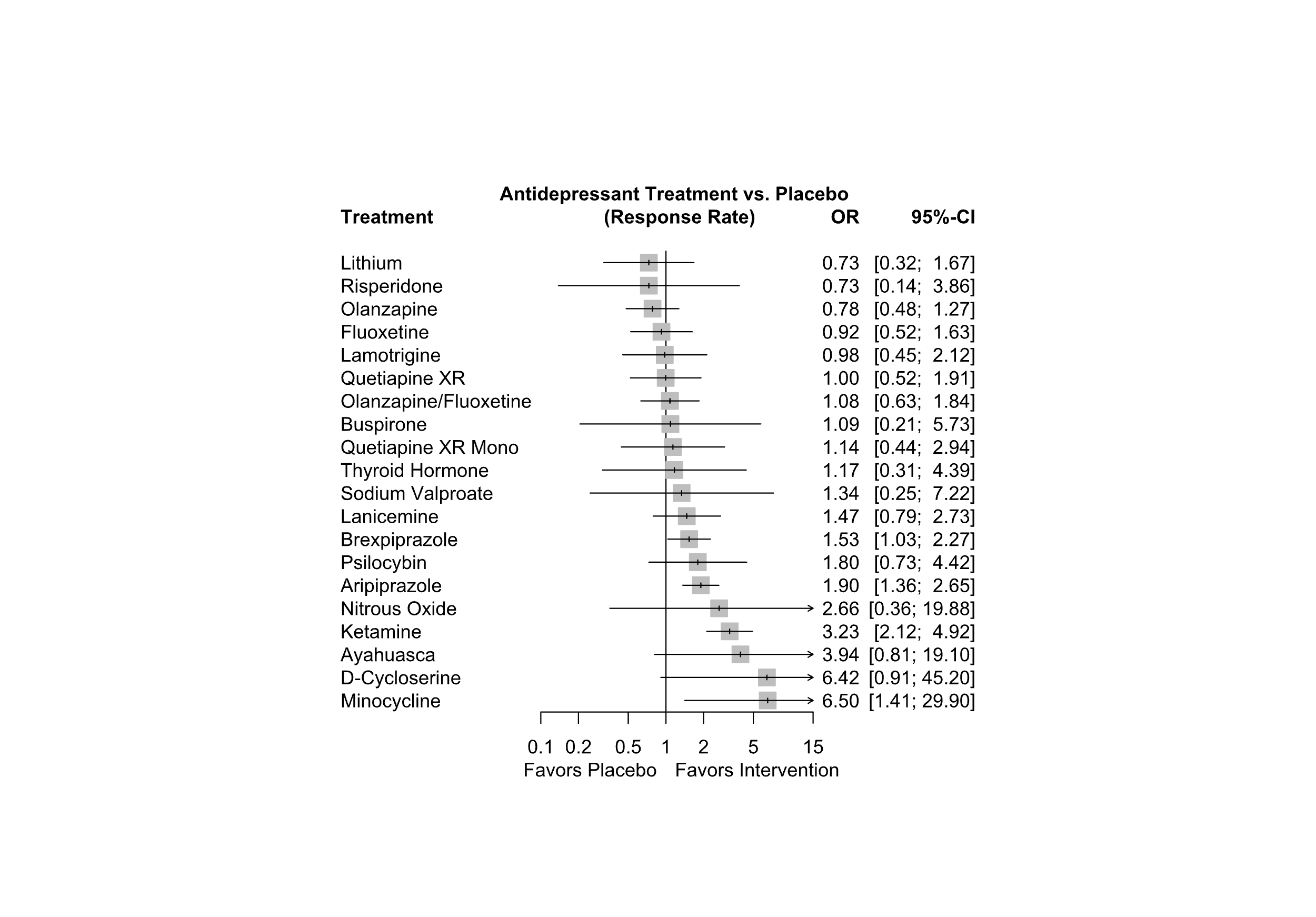


Abbreviations: CI: confidence interval; OR: odds ratio; XR: extended release

### **Supplementary Figure S10.2. Forest plot excluding studies using placebo conditions**


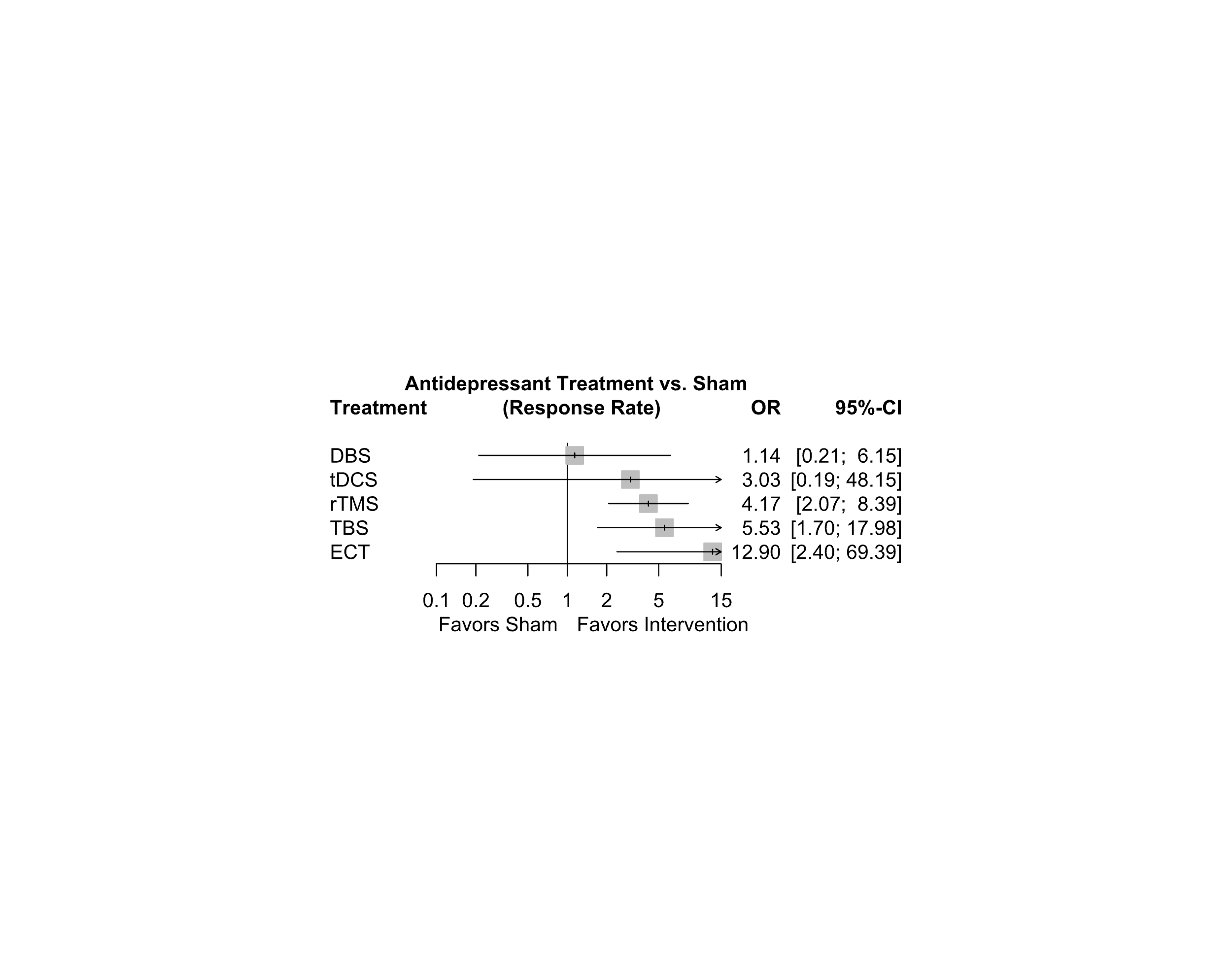


Abbreviations: CI: confidence interval; DBS: deep brain stimulation; ECT: electroconvulsive therapy; OR: odds ratio; rTMS: repetitive transcranial magnetic therapy; TBS: theta burst stimulation; tDCS: transcranial direct current stimulation

## **Supplementary Figure S10.3. Forest plot excluding studies that did not use a placebo condition**


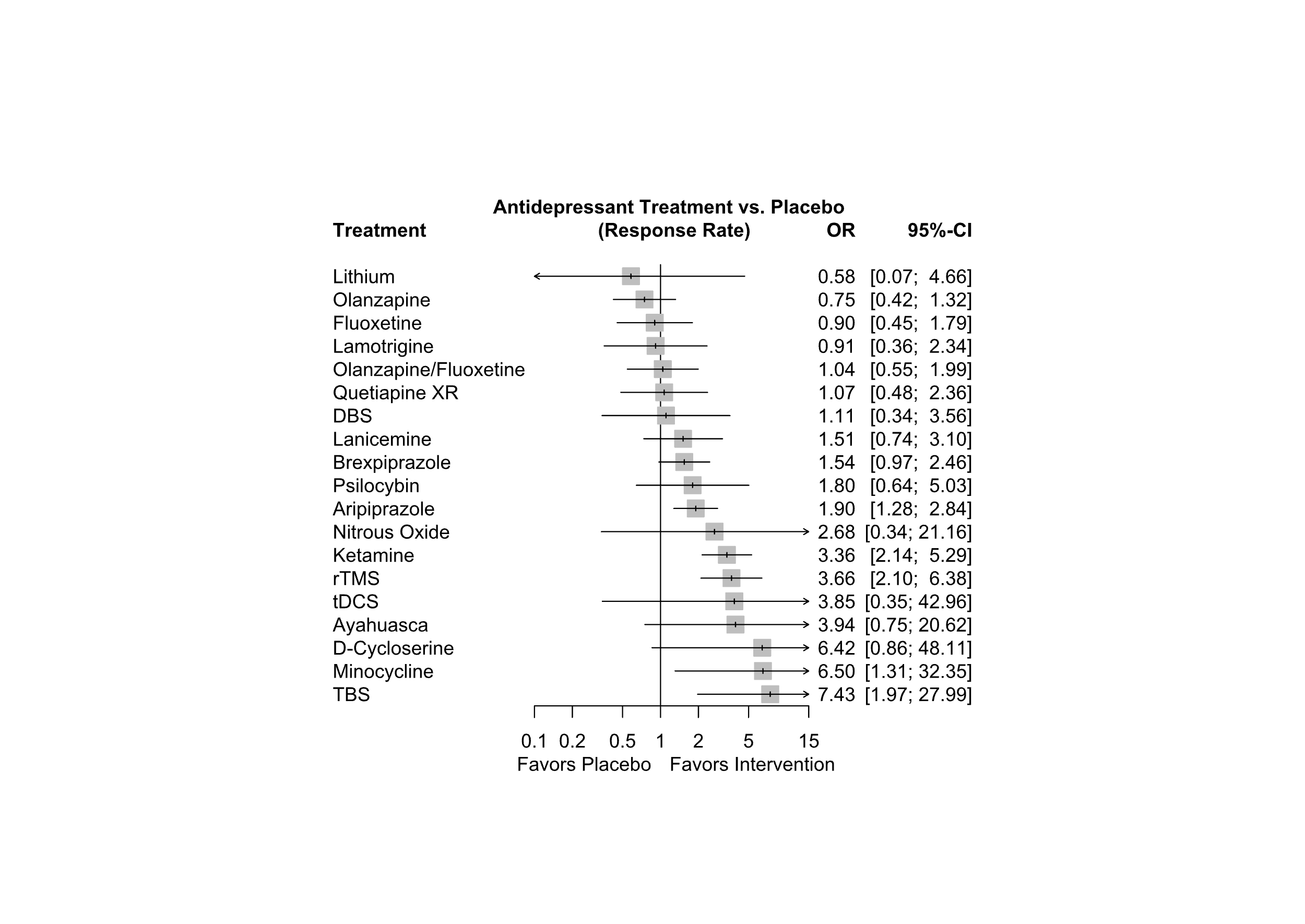


Abbreviations: CI: confidence interval; DBS: deep brain stimulation; OR: odds ratio; rTMS: repetitive transcranial magnetic therapy; TBS: theta burst stimulation; tDCS: transcranial direct current stimulation; XR: extended release

## **Supplementary Figure S10.4. Forest plot excluding studies that did not blind participants**


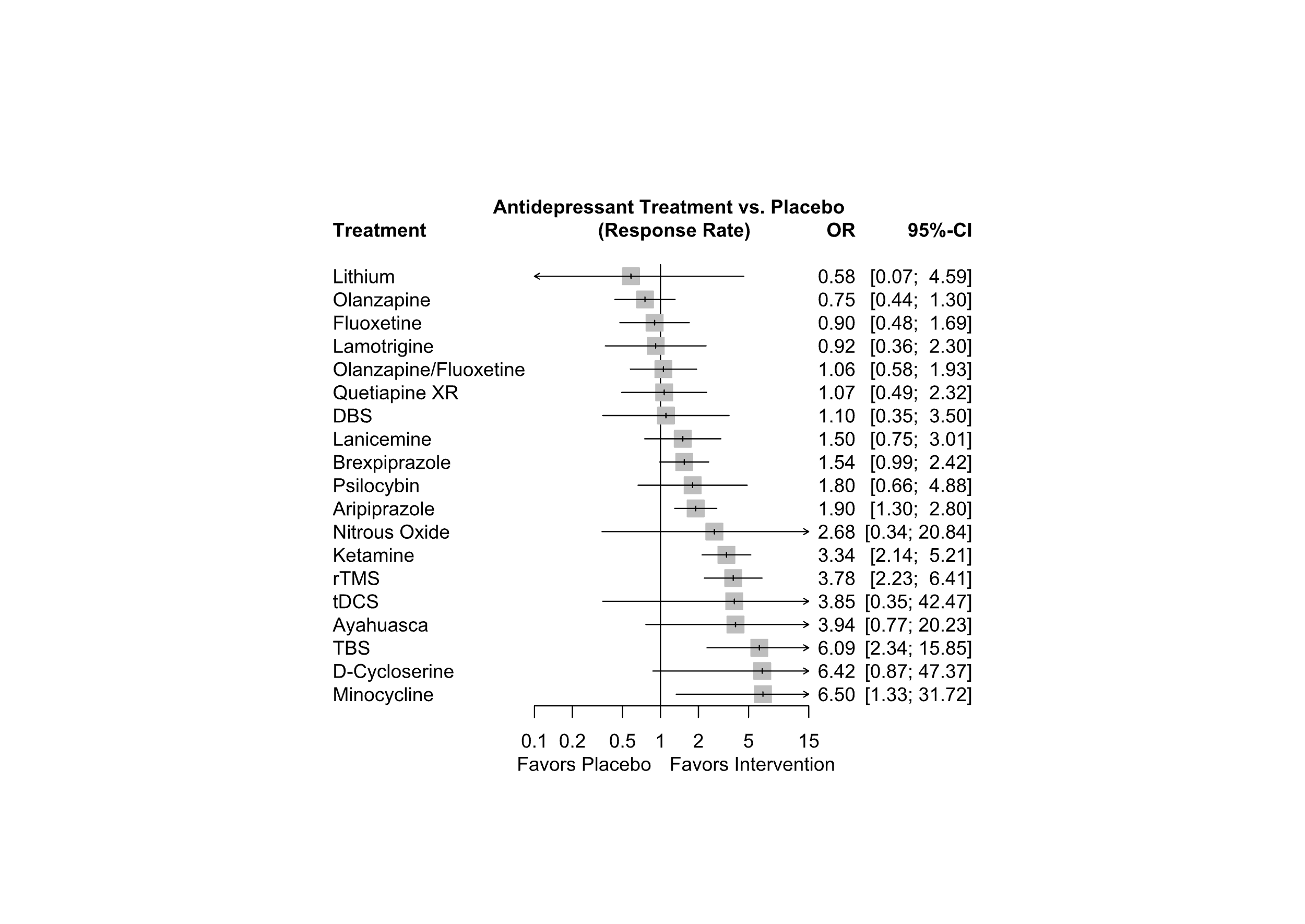


Abbreviations: CI: confidence interval; DBS: deep brain stimulation; OR: odds ratio; rTMS: repetitive transcranial magnetic therapy; TBS: theta burst stimulation; tDCS: transcranial direct current stimulation; XR: extended release

## **Supplementary Figure S10.5. Forest plot excluding studies with a high risk of bias**


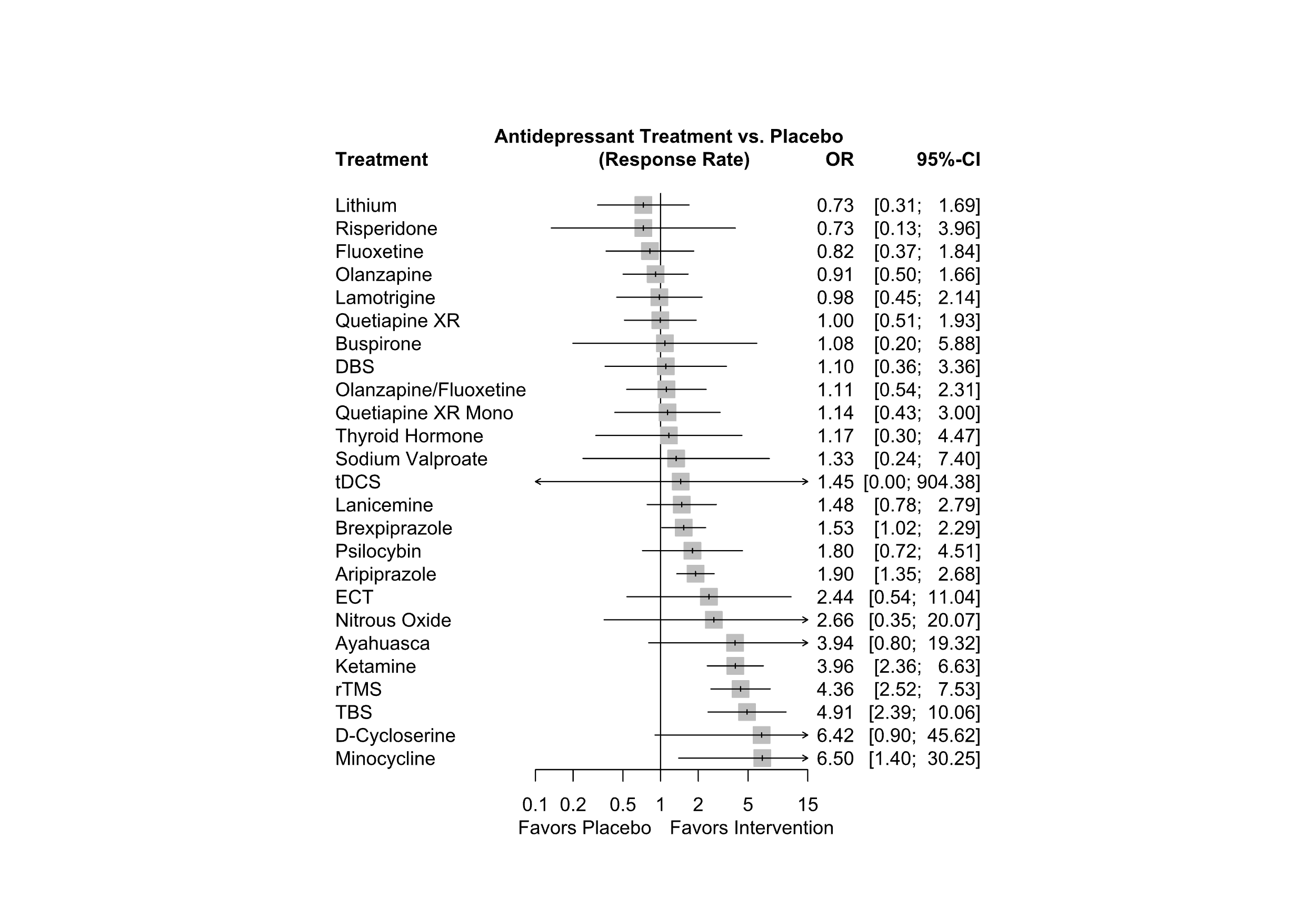


Abbreviations: CI: confidence interval; DBS: deep brain stimulation; ECT: electroconvulsive therapy; OR: odds ratio; rTMS: repetitive transcranial magnetic therapy; TBS: theta burst stimulation; tDCS: transcranial direct current stimulation; XR: extended release

## **Supplementary Figure S10.6. Forest plot including only studies with non-commercial sponsorship**

**
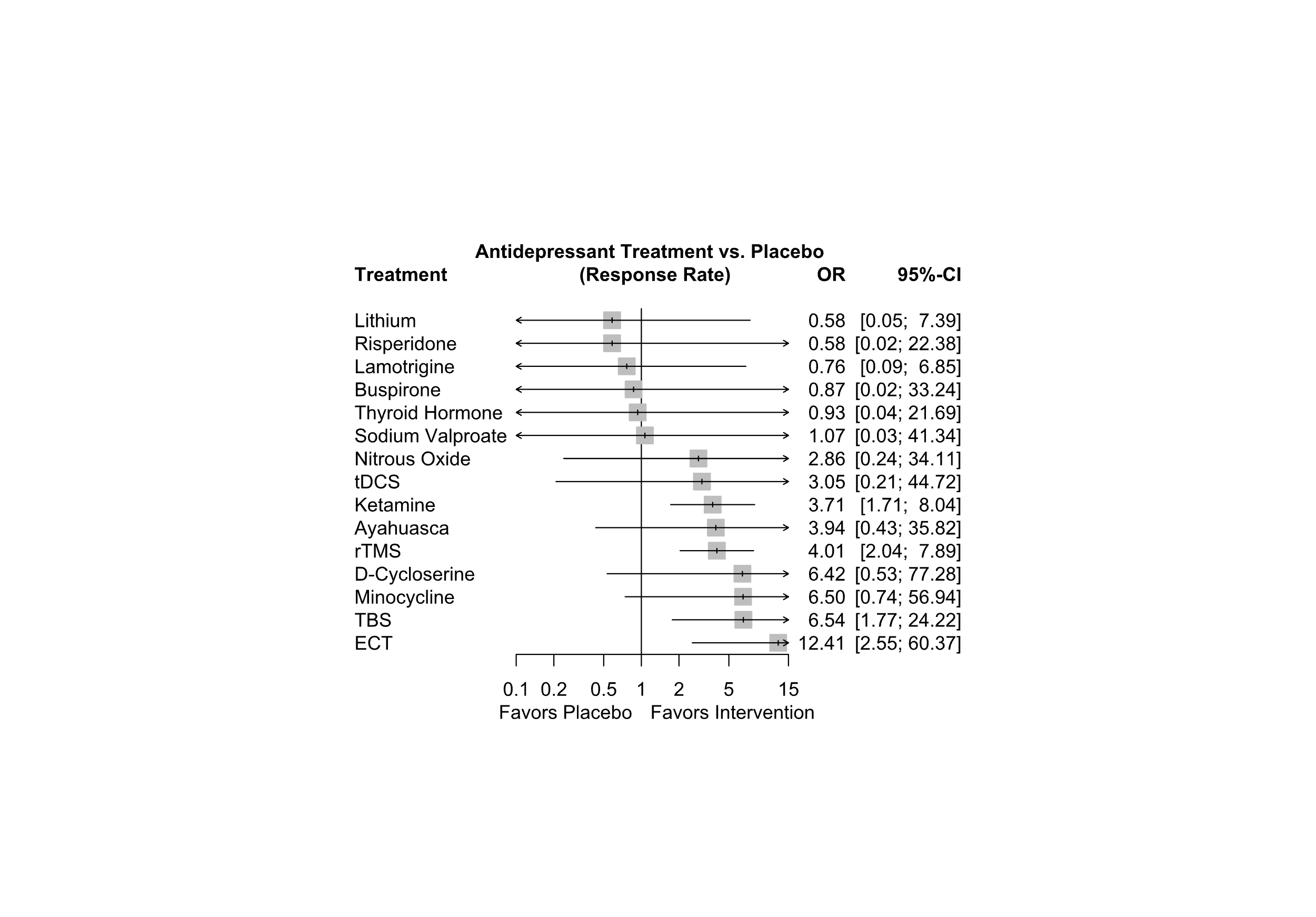
**

Abbreviations: CI: confidence interval; ECT: electroconvulsive therapy; OR: odds ratio; rTMS: repetitive transcranial magnetic therapy; TBS: theta burst stimulation; tDCS: transcranial direct current stimulation.

**Supplementary Figure S10.7. Forest plot excluding studies where response rate was imputed**  **
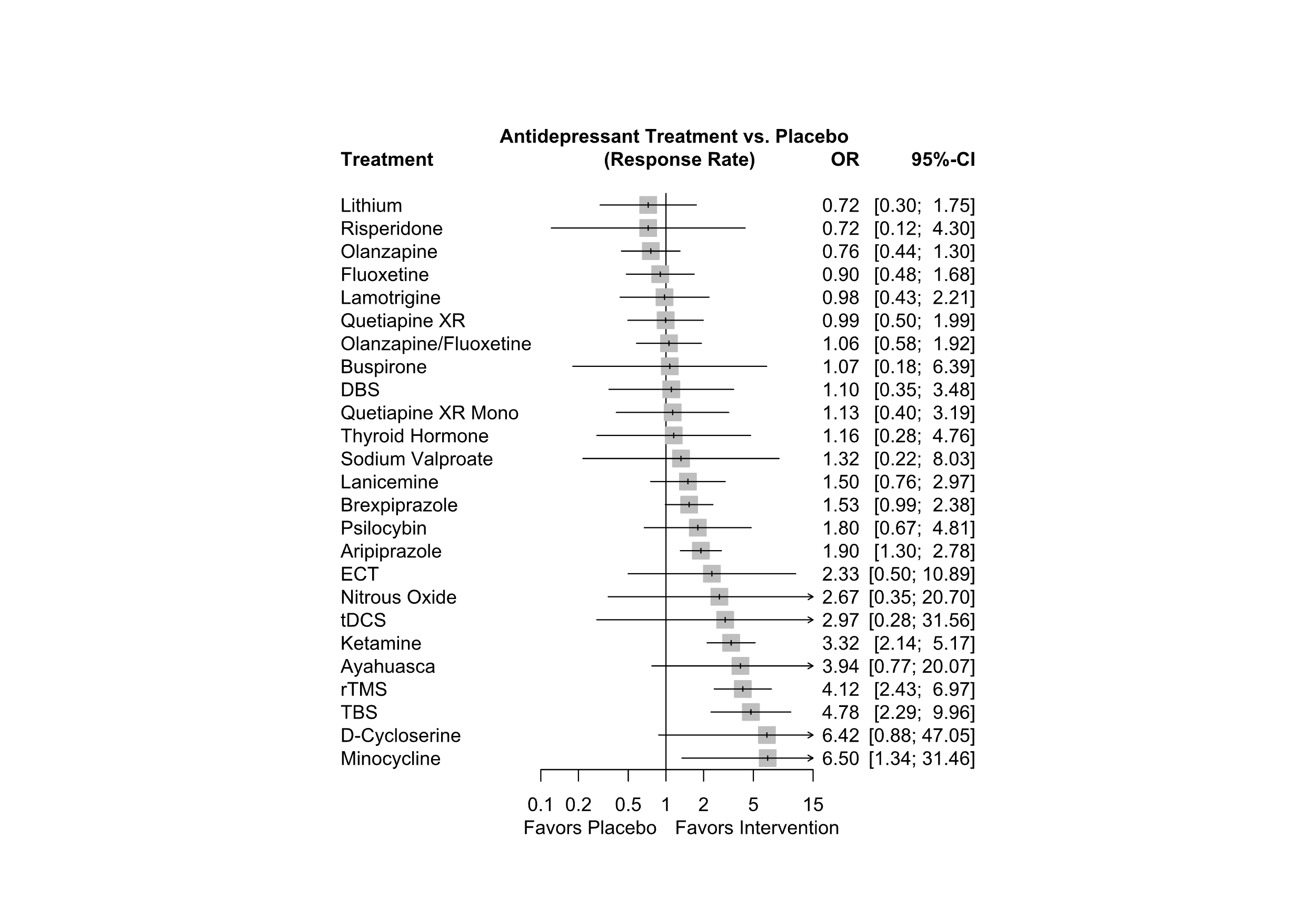
**

Abbreviations: CI: confidence interval; DBS: deep brain stimulation; ECT: electroconvulsive therapy; OR: odds ratio; rTMS: repetitive transcranial magnetic therapy; TBS: theta burst stimulation; tDCS: transcranial direct current stimulation; XR: extended release

# 11. Subanalyses

The following subanalyses examined only the subset of included studies that involved ketamine. This was done to examine possible differences between (*S*)-ketamine and racemic ketamine as well as different routes of administration. We also conducted an analysis including the TRANSFORM 1-3 studies which were pivotal for the approval of SPRAVATO for the treatment of treatment resistant depression[1-3]. This was done in a separate analysis because these studies use a different study design, which compares the verum plus an open label oral antidepressant that is newly initiated, to a placebo with an oral antidepressant. Readers should note that including these studies may lead to an overestimation of the effect of (*S*)-ketamine since the comparison to a placebo instead of a placebo plus an antidepressant might inflate the effect size of this treatment.

## **Racemic ketamine vs. (S)-ketamine**

### **Supplementary Figure S11.1. Forest plot comparing response rate of ketamine and (*S*)-ketamine**

**
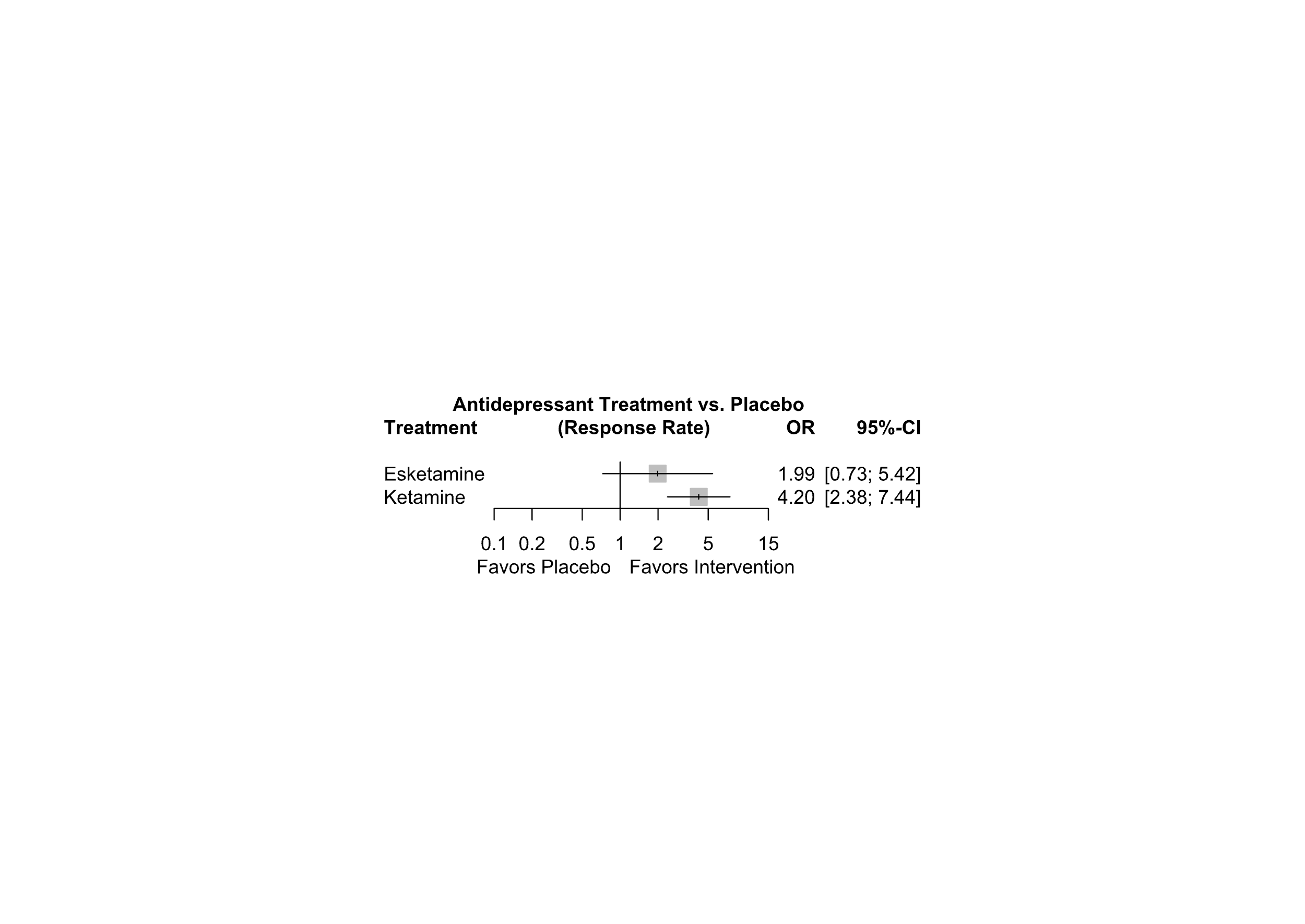
**

Abbreviations: CI: confidence interval; OR: odds ratio

### **Supplementary Table S11.1. Netleague table comparing response rates to ketamine, (*S*)-ketamine and placebo**

| **(*S*)-ketamine** |  |  |
| --- | --- | --- |
| 0.47 (0.15-1.50) | **Ketamine** |  |
| 1.99 (0.73-5.42) | 4.20 (2.38-7.44) | **Placebo** |

## **Racemic ketamine versus (S)-ketamine by route of administration**

### **Supplementary Figure S11.2. Forest plot comparing response rate to different forms of ketamine**


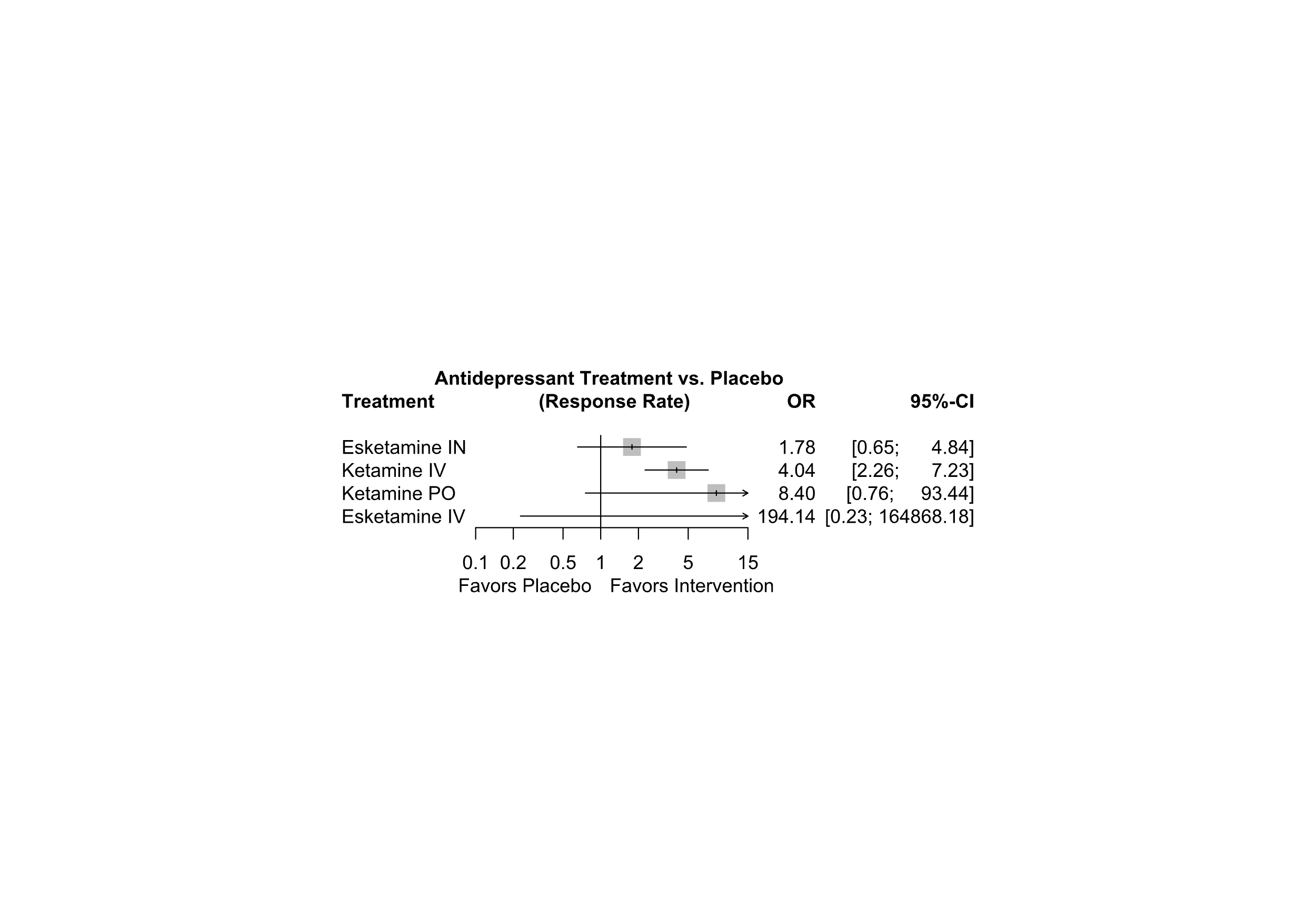


Abbreviations: CI: confidence interval; IN: intranasal; IV: intravenous; OR: odds ratio; PO: oral

### **Supplementary Table S11.2. Netleague table comparing response rates to different forms of ketamine and placebo**

| **(*S*)-ketamine IN** |  |  |  |  |
| --- | --- | --- | --- | --- |
| 0.01 (0.00-8.37) | **(*S*)-ketamine IV** |  |  |  |
| 0.44 (0.14-1.40) | 48.07 (0.06-41855.94) | **Ketamine IV** |  |  |
| 0.21 (0.02-2.87) | 23.11 (0.02-29793.02) | 0.48 (0.04-5.73) | **Ketamine PO** |  |
| 1.78 (0.65-4.84) | 194.14 (0.23-164868.18) | 4.04 (2.26-7.23) | 8.40 (0.76-93.44) | **Placebo** |

Abbreviations: IN: intranasal; IV: intravenous; OR: odds ratio; PO: oral

## **Racemic ketamine vs. (S)-ketamine including TRANSFORM 1-3 studies**

### **Supplementary Figure S11.3. Forest plot comparing response rates of ketamine and (*S*)-ketamine, including the TRANSFORM 1-3 studies**


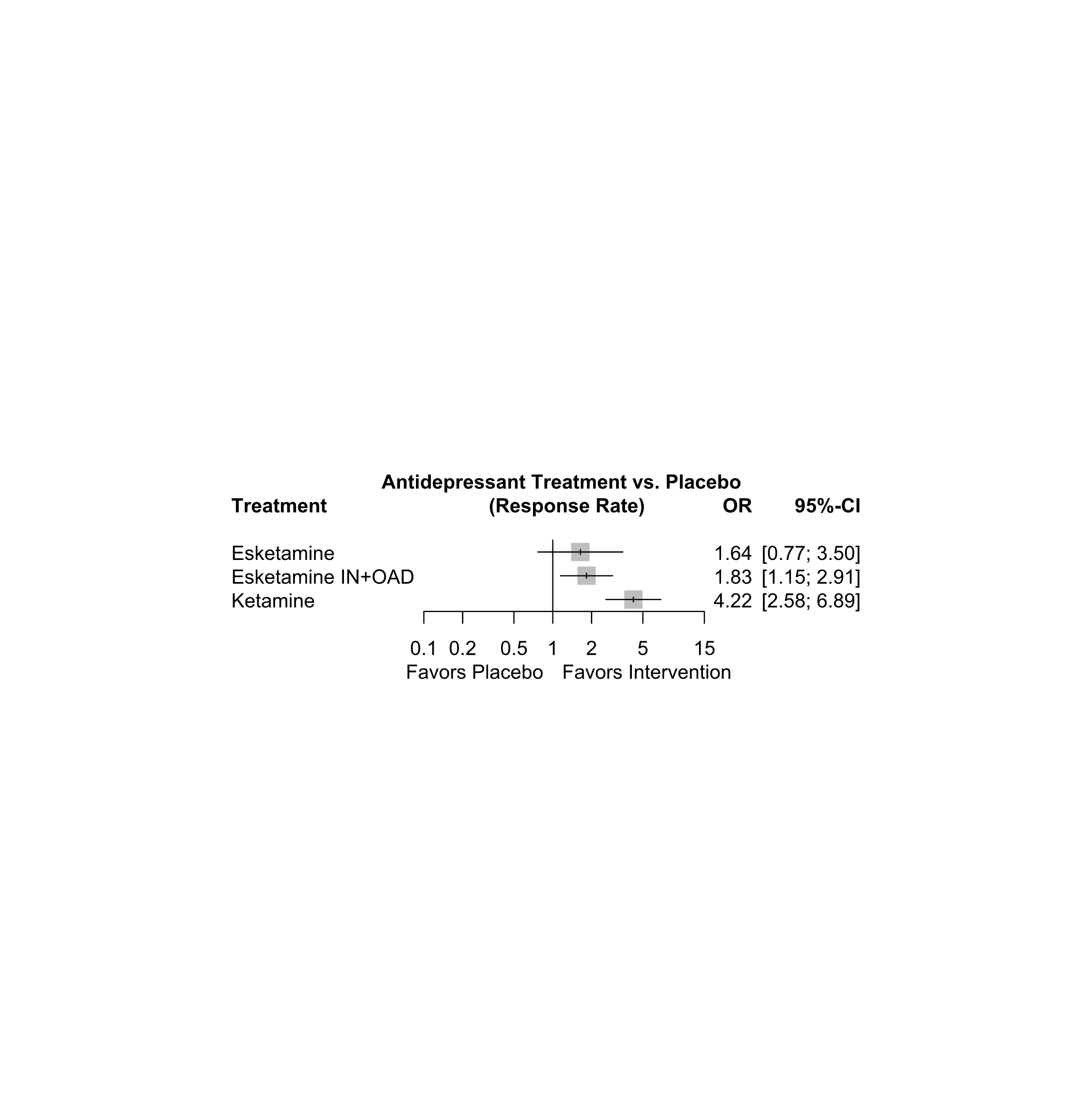


Abbreviations: CI: confidence interval; IN: intranasal; OAD: oral antidepressant; OR: odds ratio

### **Supplementary Table S11.3. Netleague table comparing response rates to ketamine, (*S*)-ketamine and placebo, including the TRANSFORM 1-3 studies**

| **Esketamine** |  |  |  |
| --- | --- | --- | --- |
| 0.90 (0.37-2.18) | **Esketamine IN+OAD** |  |  |
| 0.39 (0.16-0.96) | 0.43 (0.22-0.85) | **Ketamine** |  |
| 1.64 (0.77-3.50) | 1.83 (1.15-2.91) | 4.22 (2.58-6.89) | **Placebo** |

Abbreviations: IN: intranasal; OAD: oral antidepressant

## **Racemic ketamine versus (S)-ketamine by route of administration, including the TRANSFORM 1-3 studies**

### **Supplementary Figure S11.4. Forest plot comparing response rate to different forms of ketamine, including the TRANSFORM 1-3 studies**


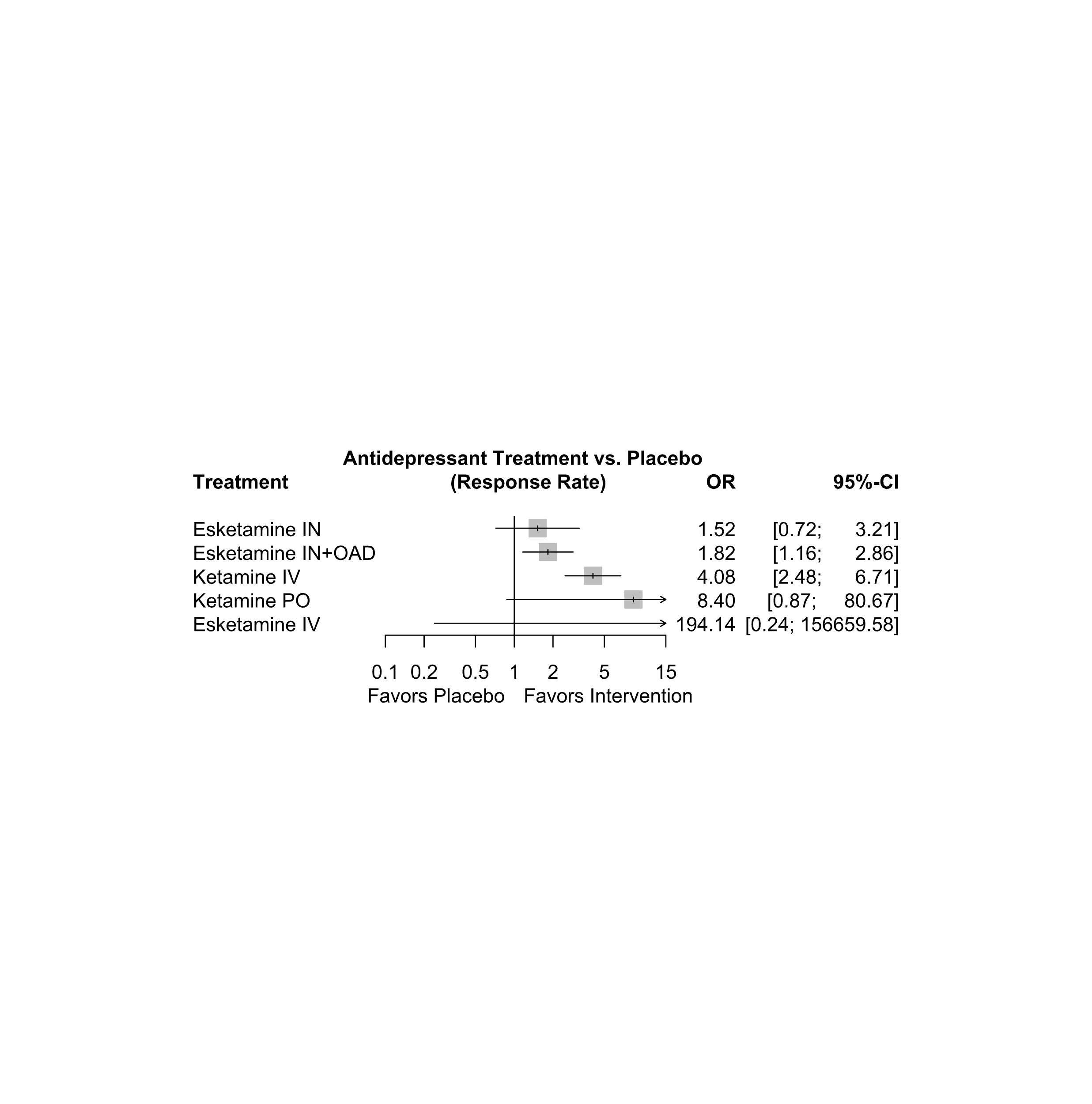


Abbreviations: CI: confidence interval; IN: intranasal; IV: intravenous; OAD: oral antidepressant; OR: odds ratio; PO: oral

### **Supplementary Table S11.4. Netleague table comparing response rates to different forms of ketamine and placebo including TRANSFORM 1-3 studies**

| **Esketamine IN** |  |  |  |  |  |
| --- | --- | --- | --- | --- | --- |
| 0.83 (0.35-1.99) | **Esketamine IN+OAD** |  |  |  |  |
| 0.01 (0.00-6.59) | 0.01 (0.00-7.70) | **Esketamine IV** |  |  |  |
| 0.37 (0.15-0.91) | 0.45 (0.23-0.87) | 47.55 (0.06-39085.22) | **Ketamine IV** |  |  |
| 0.18 (0.02-1.96) | 0.22 (0.02-2.18) | 23.11 (0.02-27053.03) | 0.49 (0.05-4.93) | **Ketamine PO** |  |
| 1.52 (0.72-3.21) | 1.82 (1.16-2.86) | 194.14 (0.24-156659.58) | 4.08 (2.48-6.71) | 8.40 (0.87-80.67) | **Placebo** |

Abbreviations: IN: intranasal; IV: intravenous; OAD: oral antidepressant; OR: odds ratio; PO: oral

**Supplementary Figure S11.5. Forest plot including TRANSFORM 1-3 studies**

**
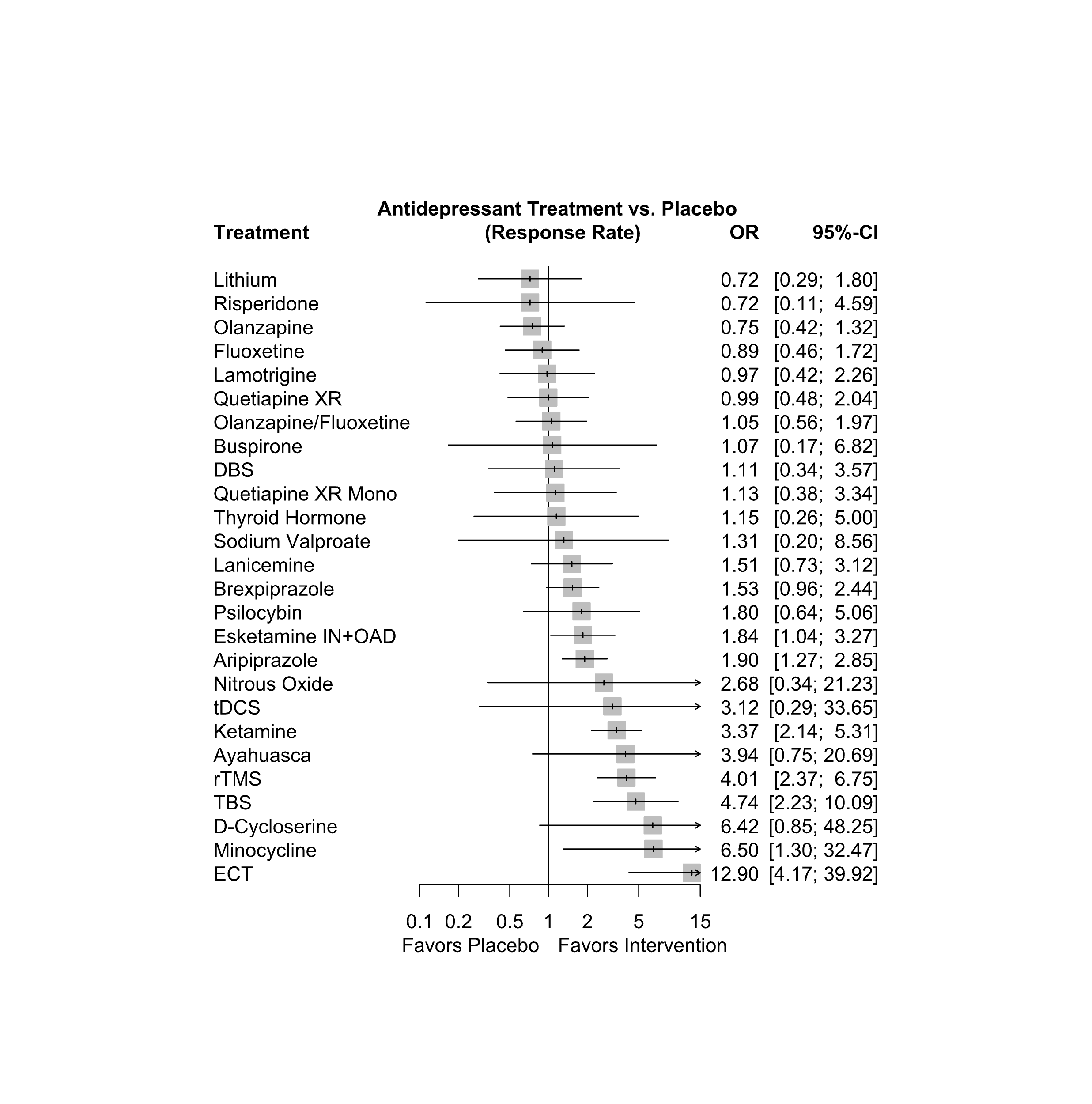
**

Abbreviations: CI: confidence interval; DBS: deep brain stimulation; ECT: electroconvulsive therapy; OAD: oral antidepressant; OR: odds ratio; rTMS: repetitive transcranial magnetic therapy; TBS: theta burst stimulation; tDCS: transcranial direct current stimulation; XR: extended release

# 12. Meta-regression

## **Supplementary Table S12. Meta-regression for variables in IV ketamine studies for the response rate outcome**

| **Variable** | **Response Rate** |  |
| --- | --- | --- |
|  | **Coefficient (95% CI)** | **B** |
| **Year of publication** | -0.65-0.88 | 0.11 |
| **Baseline** **depression score** | -0.54-1.33 | 0.38 |
| **Sex** | -0.6-0.59 | 0.01 |
| **Age** | -1.44-0.1 | -0.63 |
| **Active Placebo** | -1.07-1.59 | 0.23 |

Abbreviations: B: coefficient for given covariate; CI: confidence interval

# Supplementary references

1 Popova V, Daly EJ, Trivedi M, Cooper K, Lane R, Lim P, et al. Efficacy and safety of flexibly dosed esketamine nasal spray combined with a newly initiated oral antidepressant in treatment-resistant depression: a randomized double-blind active-controlled study. Am J Psychiatry. 2019;176(6):428-38.

2 Ochs-Ross R, Daly EJ, Zhang Y, Lane R, Lim P, Morrison RL, et al. Efficacy and safety of esketamine nasal spray plus an oral antidepressant in elderly patients with treatment-resistant depression-TRANSFORM-3. Am J Geriatr Psychiatry. 2020;28(2):121-41.

3 Fedgchin M, Trivedi M, Daly EJ, Melkote R, Lane R, Lim P, et al. Efficacy and safety of fixed-dose esketamine nasal spray combined with a new oral antidepressant in treatment-resistant depression: results of a randomized, double-blind, active-controlled Study (TRANSFORM-1). Int J Neuropsychopharmacol. 2019;22(10):616-30.

4 Berman RM, Fava M, Thase ME, Trivedi MH, Swanink R, McQuade RD, et al. Aripiprazole augmentation in major depressive disorder: a double-blind, placebo-controlled study in patients with inadequate response to antidepressants. CNS Spectr. 2009;14(4):197-206.

5 Berman RM, Marcus RN, Swanink R, McQuade RD, Carson WH, Corey-Lisle PK, et al. The efficacy and safety of aripiprazole as adjunctive therapy in major depressive disorder: a multicenter, randomized, double-blind, placebo-controlled study. J Clin Psychiatry. 2007;68(6):843-53.

6 Kamijima K, Kimura M, Kuwahara K, Kitayama Y, Tadori Y. Randomized, double-blind comparison of aripiprazole/sertraline combination and placebo/sertraline combination in patients with major depressive disorder. Psychiatry Clin Neurosci. 2018;72(8):591-601.

7 Kamijima K, Higuchi T, Ishigooka J, Ohmori T, Ozaki N, Kanba S, et al. Aripiprazole augmentation to antidepressant therapy in Japanese patients with major depressive disorder: a randomized, double-blind, placebo-controlled study (ADMIRE study). J Affect Disord. 2013;151(3):899-905.

8 Marcus RN, McQuade RD, Carson WH, Hennicken D, Fava M, Simon JS, et al. The efficacy and safety of aripiprazole as adjunctive therapy in major depressive disorder: a second multicenter, randomized, double-blind, placebo-controlled study. J Clin Psychopharmacol. 2008;28(2):156-65.

9 Otsuka Pharmaceutical Development & Commercialization Inc. Study to evaluate the efficacy, safety and tolerability of an oral aripiprazole/​escitalopram combination therapy in participants with major depressive disorder (MDD). Available at: <https://clinicaltrials.gov/study/NCT01111565>. 2021.

10 Palhano-Fontes F, Barreto D, Onias H, Andrade KC, Novaes MM, Pessoa JA, et al. Rapid antidepressant effects of the psychedelic ayahuasca in treatment-resistant depression: a randomized placebo-controlled trial. Psychol Med. 2019;49(4):655-63.

11 Bauer M, Hefting N, Lindsten A, Josiassen MK, Hobart M. A randomised, placebo-controlled 24-week study evaluating adjunctive brexpiprazole in patients with major depressive disorder. Acta Neuropsychiatr. 2019;31(1):27-35.

12 Hobart M, Skuban A, Zhang P, Josiassen MK, Hefting N, Augustine C, et al. Efficacy and safety of flexibly dosed brexpiprazole for the adjunctive treatment of major depressive disorder: a randomized, active-referenced, placebo-controlled study. Curr Med Res Opin. 2018;34(4):633-42.

13 Thase ME, Youakim JM, Skuban A, Hobart M, Augustine C, Zhang P, et al. Efficacy and safety of adjunctive brexpiprazole 2 mg in major depressive disorder: a phase 3, randomized, placebo-controlled study in patients with inadequate response to antidepressants. J Clin Psychiatry. 2015;76(9):1224-31.

14 Thase ME, Youakim JM, Skuban A, Hobart M, Zhang P, McQuade RD, et al. Adjunctive brexpiprazole 1 and 3 mg for patients with major depressive disorder following inadequate response to antidepressants: a phase 3, randomized, double-blind study. J Clin Psychiatry. 2015;76(9):1232-40.

15 Hobart M, Skuban A, Zhang P, Augustine C, Brewer C, Hefting N, et al. A randomized, placebo-controlled study of the efficacy and safety of fixed-dose brexpiprazole 2 mg/d as adjunctive treatment of adults with major depressive disorder. J Clin Psychiatry. 2018;79(4):17m12058-17m58.

16 Heresco-Levy U, Gelfin G, Bloch B, Levin R, Edelman S, Javitt DC, et al. A randomized add-on trial of high-dose d-cycloserine for treatment-resistant depression. Int J Neuropsychopharmacol. 2013;16(3):501-06.

17 Dougherty DD, Rezai AR, Carpenter LL, Howland RH, Bhati MT, O'Reardon JP, et al. A randomized sham-controlled trial of deep brain stimulation of the ventral capsule/ventral striatum for chronic treatment-resistant depression. Biol Psychiatry. 2015;78(4):240-48.

18 Holtzheimer PE, Husain MM, Lisanby SH, Taylor SF, Whitworth LA, McClintock S, et al. Subcallosal cingulate deep brain stimulation for treatment-resistant depression: a multisite, randomised, sham-controlled trial. Lancet Psychiatry. 2017;4(11):839-49.

19 Keshtkar M, Ghanizadeh A, Firoozabadi A. Repetitive transcranial magnetic stimulation versus electroconvulsive therapy for the treatment of major depressive disorder, a randomized controlled clinical trial. J ECT. 2011;27(4):310-14.

20 Rosa MA, Gattaz WF, Pascual-Leone A, Fregni F, Rosa MO, Rumi DO, et al. Comparison of repetitive transcranial magnetic stimulation and electroconvulsive therapy in unipolar non-psychotic refractory depression: a randomized, single-blind study. Int J Neuropsychopharmacol. 2006;9(6):667-76.

21 Ramasubramanian, Vikhram, Mathumathi S, Rajendhiran G, Bijulakshmi P, Kannan M. A comparative study of the effect of electroconvulsive therapy and transcranial direct current stimulation in the treatment of persons suffering from treatment‑resistant depression. Ind Psychiatry J. 2022;31(1):68-73.

22 Eli Lilly and Company. A study of olanzapine and fluoxetine for treatment-resistant depression. Available at: <https://clinicaltrials.gov/study/NCT01687478?tab=results>. 2019.

23 Thase ME, Corya SA, Osuntokun O, Case M, Henley DB, Sanger TM, et al. A randomized, double-blind comparison of olanzapine/fluoxetine combination, olanzapine, and fluoxetine in treatment-resistant major depressive disorder. J Clin Psychiatry. 2007;68(2):224-36.

24 Singh JB, Fedgchin M, Daly E, Xi L, Melman C, De Bruecker G, et al. Intravenous esketamine in adult treatment-resistant depression: a double-blind, double-randomization, placebo-controlled study. Biol Psychiatry. 2016;80(6):424-31.

25 Ahmed GK, Elserogy YM, Elfadl GMA, Ghada Abdelsalam K, Ali MA. Antidepressant and anti-suicidal effects of ketamine in treatment-resistant depression associated with psychiatric and personality comorbidities: a double-blind randomized trial. J Affect Disord. 2023;325(July 2022):127-34.

26 Chen MH, Li CT, Lin WC, Hong CJ, Tu PC, Bai YM, et al. Cognitive function of patients with treatment-resistant depression after a single low dose of ketamine infusion. J Affect Disord. 2018;241(201):1-7.

27 Fava M, Freeman MP, Flynn M, Judge H, Hoeppner BB, Cusin C, et al. Double-blind, placebo-controlled, dose-ranging trial of intravenous ketamine as adjunctive therapy in treatment-resistant depression (TRD). Mol Psychiatry. 2020;25(7):1592-603.

28 Ionescu DF, Bentley KH, Eikermann M, Taylor N, Johnson-Akeju O, Swee MB, et al. Repeat-dose ketamine augmentation for treatment-resistant depression with chronic suicidal ideation: a randomized, double blind, placebo controlled trial. J Affect Disord. 2019;243(August 2018):516-24.

29 Li CT, Chen MH, Lin WC, Hong CJ, Yang BH, Liu RS, et al. The effects of low-dose ketamine on the prefrontal cortex and amygdala in treatment-resistant depression: a randomized controlled study. Hum Brain Mapp. 2016;37(3):1080-90.

30 Murrough JW, Iosifescu DV, Chang LC, Al Jurdi RK, Green CE, Perez AM, et al. Antidepressant efficacy of ketamine in treatment-resistant major depression: a two-site randomized controlled trial. Am J Psychiatry. 2013;170(10):1134-42.

31 Shiroma PR, Thuras P, Wels J, Albott CS, Erbes C, Tye S, et al. A randomized, double-blind, active placebo-controlled study of efficacy, safety, and durability of repeated vs single subanesthetic ketamine for treatment-resistant depression. Transl Psychiatry. 2020;10(1).

32 Singh JB, Fedgchin M, Daly EJ, De Boer P, Cooper K, Lim P, et al. A double-blind, randomized, placebo-controlled, dose-frequency study of intravenous ketamine in patients with treatment-resistant depression. Am J Psychiatry. 2016;173(8):816-26.

33 Su T-p, Li C-t, Lin W-c, Wu H-j, Tsai S-j. Regular research article trial of low-dose ketamine infusion in patients with treatment-resistant depression and prominent suicidal ideation. Int J Neuropsychopharmacol. 2023(March):1-9.

34 Daly EJ, Singh JB, Fedgchin M, Cooper K, Lim P, Shelton RC, et al. Efficacy and safety of intranasal esketamine adjunctive to oral antidepressant therapy in treatment-resistant depression: a randomized clinical trial. JAMA Psychiatry. 2018;75(2):139-48.

35 Takahashi N, Yamada A, Shiraishi A, Shimizu H, Goto R, Tominaga Y. Efficacy and safety of fixed doses of intranasal esketamine as an add-on therapy to oral antidepressants in Japanese patients with treatment-resistant depression: a phase 2b randomized clinical study. BMC Psychiatry. 2021;21(1):1-13.

36 Domany Y, Bleich-Cohen M, Tarrasch R, Meidan R, Litvak-Lazar O, Stoppleman N, et al. Repeated oral ketamine for out-patient treatment of resistant depression: randomised, double-blind, placebo-controlled, proof-of-concept study. Br J Psychiatry. 2019;214(1):20-26.

37 Barbee JG, Thompson TR, Jamhour NJ, Stewart JW, Conrad EJ, Reimherr FW, et al. A double-blind placebo-controlled trial of lamotrigine as an antidepressant augmentation agent in treatment-refractory unipolar depression. J Clin Psychiatry. 2011;72(10):1405-12.

38 Santos MA, Rocha FL, Hara C. Efficacy and safety of antidepressant augmentation with lamotrigine in patients with treatment-resistant depression: a randomized, placebo-controlled, double-blind study. Prim Care Companion J Clin Psychiatry. 2008;10(3):187-90.

39 Schindler F, Anghelescu IG. Lithium versus lamotrigine augmentation in treatment resistant unipolar depression: a randomized, open-label study. Int Clin Psychopharmacol. 2007;22(3):179-82.

40 Sanacora G, Smith MA, Pathak S, Su HL, Boeijinga PH, McCarthy DJ, et al. Lanicemine: a low-trapping NMDA channel blocker produces sustained antidepressant efficacy with minimal psychotomimetic adverse effects. Mol Psychiatry. 2014;19(9):978-85.

41 Sanacora G, Johnson MR, Khan A, Atkinson SD, Riesenberg RR, Schronen JP, et al. Adjunctive lanicemine (AZD6765) in patients with major depressive disorder and history of inadequate response to antidepressants: a randomized, placebo-controlled study. Neuropsychopharmacology. 2017;42(4):844-53.

42 Nierenberg AA, Papakostas GI, Petersen T, Montoya HD, Worthington JJ, Tedlow J, et al. Lithium augmentation of nortriptyline for subjects resistant to multiple antidepressants. J Clin Psychopharmacol. 2003;23(1):92-95.

43 Nierenberg AA, Fava M, Trivedi MH, Wisniewski SR, Thase ME, McGrath PJ, et al. A comparison of lithium and T3 augmentation following two failed medication treatments for depression: a STAR*D report. Am J Psychiatry. 2006;163(9):1519-30.

44 Husain MI, Chaudhry IB, Husain N, Khoso AB, Rahman RR, Hamirani MM, et al. Minocycline as an adjunct for treatment-resistant depressive symptoms: a pilot randomised placebo-controlled trial. J Psychopharmacol. 2017;31(9):1166-75.

45 Nagele P, Duma A, Kopec M, Gebara MA, Parsoei A, Walker M, et al. Nitrous oxide for treatment-resistant major depression: a proof-of-concept trial. Biol Psychiatry. 2015;78(1):10-18.

46 Yan D, Liu B, Wei X, Ou W, Liao M, Ji S, et al. Efficacy and safety of nitrous oxide for patients with treatment-resistant depression, a randomized controlled trial. Psychiatry Res. 2022;317(September):114867-67.

47 Corya SA, Williamson D, Sanger TM, Briggs SD, Case M, Tollefson G. A randomized, double-blind comparison of olanzapine/fluoxetine combination, olanzapine, fluoxetine, and venlafaxine in treatment-resistant depression. Depress Anxiety. 2006;23(6):364-72.

48 Shelton RC, Williamson DJ, Corya SA, Sanger TM, Van Campen LE, Case M, et al. Olanzapine/fluoxetine combination for treatment-resistant depression: a controlled study of SSRI and nortriptyline resistance. J Clin Psychiatry. 2005;66(10):1289-97.

49 Goodwin GM, Aaronson ST, Alvarez O, Arden PC, Baker A, Bennett JC, et al. Single-dose psilocybin for a treatment-resistant episode of major depression. N Engl J Med. 2022;387(18):1637-48.

50 Astellas Pharma Inc. Study to evaluate the effect and safety of quetiapine extended release (XR) (FK949E) in major depressive disorder. Available at: <https://clinicaltrials.gov/ct2/show/NCT01725282>. 2014.

51 Bauer M, Dell'Osso L, Kasper S, Pitchot W, Vansvik ED, Köhler J, et al. Extended-release quetiapine fumarate (quetiapine XR) monotherapy and quetiapine XR or lithium as add-on to antidepressants in patients with treatment-resistant major depressive disorder. J Affect Disord. 2013;151(1):209-19.

52 Akpinar K, Kalkan Oğuzhanoğlu N, Toker Uğurlu T. Efficacy of transcranial magnetic stimulation in treatment-resistant depression. Turk J Med. 2022;52(4):1344-54.

53 Avery DH, Holtzheimer PE, Fawaz W, Russo J, Neumaier J, Dunner DL, et al. A controlled study of repetitive transcranial magnetic stimulation in medication-resistant major depression. Biol Psychiatry. 2006;59(2):187-94.

54 Bakim B, Uzun UE, Karamustafalioglu O, Ozcelik B, Alpak G, Tankaya O, et al. The combination of antidepressant drug therapy and high-frequency repetitive transcranial magnetic stimulation in medication-resistant depression. Klinik Psikofarmakoloji Bulteni. 2012;22(3):244-53.

55 Blumberger DM, Maller JJ, Thomson L, Mulsant BH, Rajji TK, Maher M, et al. Unilateral and bilateral MRI-targeted repetitive transcranial magnetic stimulation for treatmentresistant depression: a randomized controlled study. J Psychiatry Neurosci. 2016;41(4):E58-E66.

56 Blumberger DM, Mulsant BH, Fitzgerald PB, Rajji TK, Ravindran AV, Young LT, et al. A randomized double-blind sham-controlled comparison of unilateral and bilateral repetitive transcranial magnetic stimulation for treatment-resistant major depression. World J Biol Psychiatry. 2012;13(6):423-35.

57 Chen SJ, Chang CH, Tsai HC, Chen ST, Lin CC. Superior antidepressant effect occurring 1 month after rTMS: add-on rTMS for subjects with medication-resistant depression. Neuropsychiatr DIs Treat. 2013;9:397-401.

58 Fitzgerald PB, Hoy KE, Herring SE, McQueen S, Peachey AVJ, Segrave RA, et al. A double blind randomized trial of unilateral left and bilateral prefrontal cortex transcranial magnetic stimulation in treatment resistant major depression. J Affect Disord. 2012;139(2):193-98.

59 Garcia-Toro M, Salva J, Daumal J, Andres J, Romera M, Lafau O, et al. High (20-Hz) and low (1-Hz) frequency transcranial magnetic stimulation as adjuvant treatment in medication-resistant depression. Psychiatry Res 2006;146(1):53-57.

60 Garcia-Toro M, Mayol A, Arnillas H, Capllonch I, Ibarra O, Crespí M, et al. Modest adjunctive benefit with transcranial magnetic stimulation in medication-resistant depression. J Affect Disord. 2001;64(2-3):271-75.

61 Pallanti S, Bernardi S, Di Rollo A, Antonini S, Quercioli L. Unilateral low frequency versus sequential bilateral repetitive transcranial magnetic stimulation: is simpler better for treatment of resistant depression? Neuroscience. 2010;167(2):323-28.

62 Theleritis C, Sakkas P, Paparrigopoulos T, Vitoratou S, Tzavara C, Bonaccorso S, et al. Two versus one high-frequency repetitive transcranial magnetic stimulation session per day for treatment-resistant depression. J ECT. 2017;33(3):190-97.

63 Triggs WJ, Ricciuti N, Ward HE, Cheng J, Bowers D, Goodman WK, et al. Right and left dorsolateral pre-frontal rTMS treatment of refractory depression: a randomized, sham-controlled trial. Psychiatry Res. 2010;178(3):467-74.

64 van Eijndhoven PFP, Bartholomeus J, Möbius M, de Bruijn A, Ferrari GRA, Mulders P, et al. A randomized controlled trial of a standard 4-week protocol of repetitive transcranial magnetic stimulation in severe treatment resistant depression. J Affect Disord. 2020;274(January):444-49.

65 Zheng H, Zhang L, Li L, Liu P, Gao J, Liu X, et al. High-frequency rTMS treatment increases left prefrontal myo-inositol in young patients with treatment-resistant depression. Prog Neuropsychopharmacol Biol Psychiatry. 2010;34(7):1189-95.

66 Bennabi D, Nicolier M, Monnin J, Tio G, Pazart L, Vandel P, et al. Pilot study of feasibility of the effect of treatment with tDCS in patients suffering from treatment-resistant depression treated with escitalopram. Clin Neurophysiol. 2015;126(6):1185-89.

67 Blumberger DM, Tran LC, Fitzgerald PB, Hoy KE, Daskalakis ZJ. A randomized double-blind sham-controlled study of transcranial direct current stimulation for treatment-resistant major depression. Front Psychiatry. 2012;3(August):1-8.

68 Cole EJ, Phillips AL, Bentzley BS, Stimpson KH, Nejad R, Barmak F, et al. Stanford neuromodulation therapy (SNT): a double-blind randomized controlled trial. Am J Psychiatry. 2022;179(2):132-41.

69 Li CT, Chen MH, Juan CH, Huang HH, Chen LF, Hsieh JC, et al. Efficacy of prefrontal theta-burst stimulation in refractory depression: a randomized sham-controlled study. Brain. 2014;137(7):2088-98.

70 Blumberger DM, Vila-Rodriguez F, Thorpe KE, Feffer K, Noda Y, Giacobbe P, et al. Effectiveness of theta burst versus high-frequency repetitive transcranial magnetic stimulation in patients with depression (THREE-D): a randomised non-inferiority trial. Lancet. 2018;391(10131):1683-92.

71 Bulteau S, Laurin A, Pere M, Fayet G, Thomas-Ollivier V, Deschamps T, et al. Intermittent theta burst stimulation (iTBS) versus 10 Hz high-frequency repetitive transcranial magnetic stimulation (rTMS) to alleviate treatment-resistant unipolar depression: a randomized controlled trial (THETA-DEP). Brain Stimul. 2022;15(3):870-80.

72 Fang Y, Yuan C, Xu Y, Chen J, Wu Z, Cao L, et al. A pilot study of the efficacy and safety of paroxetine augmented with risperidone, valproate, buspirone, trazodone, or thyroid hormone in adult chinese patients with treatment-resistant major depression. J Clin Psychopharmacol. 2011;31(5):638-42.
